# The combined role of education and wealth in menstrual health: Evidence from Bangladesh

**DOI:** 10.1371/journal.pone.0346457

**Published:** 2026-10-05

**Authors:** Mohammad Hridoy Patwary, Md Tazvir Amin, Sumaiya Nusrat, Md Mahabubur Rahman

**Affiliations:** 1 Health Systems and Population Studies Division, icddr, b, Dhaka, Bangladesh; 2 Maternal and Child Health Division, icddr, b, Dhaka, Bangladesh; Cranfield University, UNITED KINGDOM OF GREAT BRITAIN AND NORTHERN IRELAND

## Abstract

**Background:**

Ensuring menstrual health remains a critical public health challenge, particularly in resource-limited settings. Both individual education and household wealth index are considered to play a crucial role in ensuring menstrual health. However, their interactive effect remains unclear, particularly in the context of Bangladesh. To address this gap, this study examines the interplay between education and wealth in determining the use of sanitary napkins in Bangladesh, with sanitary napkin use as a proxy for menstrual health. This choice reflects the predominant usage of sanitary napkins for menstrual management in Bangladesh.

**Methods:**

We used nationally representative data from the 2019 Bangladesh Multiple Indicator Cluster Survey (MICS). Sanitary napkin use by women during their last menstruation was used as the outcome variable. A generalized linear model with a logit link function was used to estimate adjusted odds ratios (AORs). Then using the fitted model, marginal probabilities of sanitary napkin use were estimated for subgroups defined by combinations of wealth index and educational attainment.

**Findings:**

The national prevalence of sanitary napkin use was 23.4%. Odds of sanitary napkin use was 4.5 times higher (AOR = 4.50, 95% CI: 3.28–6.16) among women with secondary or higher education compared to those with no formal education. Similarly, women from the upper three wealth quintiles had 2.3 times higher odds (AOR = 2.30, 95% CI: 1.58–3.34) of using sanitary napkins compared to those in the lowest two quintiles. Women with secondary or higher education from lower wealth quintiles were more likely to use sanitary napkins than with pre-primary or no education from wealthier households (marginal probability: 15.46% vs 9.25%).

**Conclusion:**

Both education and wealth play a critical role in determining menstrual health among Bangladeshi women. While financial constraints remain a key barrier, the findings suggest that improving individual education could significantly enhance hygienic menstrual practices, even among lower-socioeconomic groups. Policies should prioritize interventions for improving individual education for better menstrual health.

## 1 Introduction

Menstruation is a natural physiological process that generally indicates the onset of reproductive lifespan for a girl, characterized by the regular discharge of blood and tissue from the uterus through the vagina every month [1]. The first occurrence of menstruation (aka menarche) usually happens between the ages of 12–15 years and represents a transformative phase in the lives of adolescent girls, bringing along new challenges and vulnerabilities [2,3]. Despite being a natural and essential bodily process, menstruation has been surrounded by taboos, myths, and secrecy in many cultures for a long time, leading to stigma, shame, and silence around the world, especially in low- and middle-income countries (LMICs) [4–6]. This stigma and shame can have significant negative impacts on the emotional well-being and self-esteem of menstruating individuals, particularly young girls as they transition into adolescence [7,8].

Each month, approximately 1.8 billion girls worldwide experience menstruation, yet millions lack the resources to manage their menstrual health in a dignified and healthy way due to limited affordability, lack of awareness, stigma, taboos, and silence about menstrual hygiene management (MHM) [9,10]. Inadequate access to appropriate menstrual health resources and supportive environments has been associated with many adverse health outcomes such as lower reproductive tract infections (RTIs), secondary infertility, and abnormal vaginal discharge [11–14]. It also affects the quality of life and performance in schools and workplaces, a significant barrier to women’s empowerment and social inclusion [15–17].

Menstrual health has become a global public health concern, emphasizing individuals’ practices and actions to maintain cleanliness, dignity, and health during menstruation [18]. This is also closely linked with physical health, social justice, and human rights [18]. Menstrual health has also become a vital focus within the Sustainable Development Goals (SDGs) due to its importance in enhancing women’s physical and mental well-being and promoting social inclusion [19]. It involves using proper menstrual hygiene products (i.e., napkins, tampons, dry cloths, menstrual cups, etc), sanitation facilities (i.e., washing the cloths, drying them in sunlight, disposal of the menstrual product properly, etc.), and healthy behaviors (changing the napkins or cloths regularly at least 4 times a day) during menstruation [19].

Recognizing the impact of inadequate MHM on health, education, and gender equality, various efforts have been undertaken in LMICs to address these challenges. Governments, international organizations, and local NGOs have implemented programs to improve access to menstrual products, sanitation facilities, and menstrual health education. Yet, the pervasive stigma and silence surrounding menstruation in LMICs like Bangladesh obstruct access to rights-based information and services regarding MHM [20]. Around half of the adolescent girls in rural Bangladesh are not very confident about managing their menstrual bleeding, even in their own house, where this percentage in school is less than 10% [20]. Poor menstrual health also affects their performance in school and overall life [21,22]. National representative data shows that 30% of school girls experienced an average monthly absence of 2.5 days due to menstruation [23]. Though the average age of menarche in Bangladesh is higher (12.8) than the global average (12.4), only a quarter of adolescent girls know about menstruation before their menarche [23–25]. Only 24.3% of women and adolescent girls in Bangladesh use proper modern absorbents, and fewer than 20% of old cloth users adhere to the recommended cleaning and drying practices [23,26].

While traditional practices such as using old cloths are deeply rooted in cultural norms and have been passed down across generations, they often lack hygienic standards and are associated with a higher risk of infection and discomfort [27]. Other alternatives, including reusable cloth pads, menstrual cups, and tampons, have gained attention as potential menstrual hygiene products due to their affordability, reusability, and environmental benefits [28,29]. However, these alternatives are not yet widely accepted or adopted in Bangladesh because of limited awareness, concerns regarding usability, and restricted availability [30,31]. In contrast, sanitary napkins are widely accepted and commercially available modern menstrual product in Bangladesh. As a result, sanitary napkin use provides a relevant indicator for assessing progress in menstrual health.

While extensive research identifies education and household wealth as critical determinants of sanitary napkin use, the literature remains fragmented. Prior studies have typically examined the factors in isolation, suggesting that higher education and better economic status are individually associated with improved MHM [26,32]. However, there is a notable gap in the literature regarding their combined and potentially interactive influence. Specifically, current evidence fails to explain whether high education can compensate for low wealth, or if financial constraints completely nullify the benefits of education. Moreover, most existing studies provide limited insights into regional disparities, despite the fact that menstrual health behaviors and barriers can vary widely by geography, culture, and local infrastructure. Therefore, this study aims to examine how women’s education and household wealth jointly shape the use of sanitary napkins, offering evidence that can inform both national and region-specific strategies to improve menstrual health in Bangladesh.

## 2 Methods

### 2.1 Data overview

This study utilized data from the 2019 Bangladesh Multiple Indicator Cluster Survey (MICS), conducted by the Bangladesh Bureau of Statistics (BBS) with technical support from UNICEF. The survey employed a two-stage stratified sampling design to collect nationally representative information on the health and well-being of women and children across all districts of Bangladesh, covering both urban and rural areas.

A total of 3,220 primary sampling units (PSUs) and 64,400 households were included in the survey. In the first stage, enumeration areas (EAs) were selected using probability proportional to size. In the second stage, 20 households were randomly selected from each selected EA. Across all clusters, this yielded a final analytical sample of 46,677 women of reproductive age. Accounting for survey design effects (DEFF = 2.0; effective N = 23,338), this sample size provides >99% power to detect an odds ratio >1.33 and 85.4% power to detect an odds ratio down to 1.10 (α=0.05).

The survey collected detailed information from women of reproductive age (15–49 years). Sampling weights provided in the dataset were applied in all analyses to account for the complex survey design and ensure national representativeness. The dataset used in this study is publicly available (accessed in January, 2023) and de-identified, with no information that could be used to identify individual participants.

### 2.2 Outcome variable

The outcome variable for this study was whether the participant used a sanitary napkin during her last menstruation. The original variable, which included various materials (e.g., cloth, tissue, cotton pads), was recoded into a binary variable: sanitary napkin vs. others. In this study, “sanitary napkin use” refers to any reported use of purpose-made sanitary pads during the last menstrual period, regardless of whether they were used exclusively or in combination with other materials. Thus, the measure captures reported use rather than sufficiency of access or adequacy of product use throughout the full menstrual cycle. Sanitary napkin use was considered a proxy for menstrual health, reflecting access to safe, absorbent, and hygienic materials compared to traditional or improvised materials.

### 2.3 Explanatory variables

Individual educational attainment and household wealth index served as the key explanatory variable in this study. Education was chosen as higher education is generally associated with better knowledge of health practices [33]. Specifically, formal schooling enhances a woman’s health literacy and her ability to access, interpret, and adopt evidence-based health information, which are core components of menstrual health awareness. On the other hand, the household wealth index reflects the economic capacity of a household to purchase menstrual hygiene products and related resources. This measure indicates household’s disposable income and its ability to prioritize health-related expenditures over basic subsistence needs. Other variables considered in this study were identified from relevant literature from Bangladesh [26], India, Pakistan, and Nepal (Table 1) [34–36]. Individual level factors that can affect use of sanitary napkin included participants age, exposure to media, and status of disability. Household level factors consists of religious belief of household along with sex and educational attainment of household head. Community level prevalence of sanitary napkin usage, type of residence and division of the participants were included as community level factor. Community-level prevalence of sanitary napkin usage was calculated as the percentage of women in each survey cluster who reported using sanitary napkins during their last menstruation.

**Table 1:** Description of variables used in this study, Bangladesh MICS 2019.

Explanatory variable	Categories	Description
**Individual-level** **factors**		
Age	15-19	Respondent’s current age is between 15 and 19 years.
	20-24	Respondent’s current age is between 20 and 24 years.
	25-29	Respondent’s current age is between 25 and 29 years.
	30-34	Respondent’s current age is between 30 and 34 years.
	35-39	Respondent’s current age is between 35 and 39 years.
	40-44	Respondent’s current age is between 40 and 44 years.
	45-49	Respondent’s current age is 45 years or above.
Educational attainment	Primary or below	No formal education
	Secondary or above	6–10 or more than 10 years of formal schooling
Media exposure	No media exposure	Respondent does not get any exposure to mass media.
	At least once a week	Get exposure at least once a week from at least one media.
	Almost everyday	Get exposure almost every day from at least one media.
Disability	Yes	Respondent has at least one disability among difficulty seeing, hearing, walking, remembering or concentrating, self-care, and communicating.
	No	Respondent has no such disabilities.
**Household-level** **factors**		
Wealth index	Bottom two	Household belongs to the bottom two wealth quantile.
	Upper three	Household belongs to the upper three wealth quantile.
Educational attainment of household head	Pre-primary or none	No formal education
	Primary	1–5 years of formal schooling
	Secondary or above	6–10 or more than 10 years of formal schooling
Sex of household head	Male	Sex of the household head is male.
	Female	Sex of the household head is female.
Religious affiliation	Muslim	Respondent belongs to a Muslim household.
	Non-Muslim	Respondent belongs to a non-Muslim household.
**Community-level** **factors**		
Community prevalence of sanitary napkin usage	Low prevalence	The community prevalence of sanitary napkins use is below 30%.
	Moderate prevalence	The community prevalence of sanitary napkins use is between 30–60%.
	High prevalence	The community prevalence of sanitary napkins use is higher than 60%.
Area of Residence	Rural	Respondent’s type of place of residence is rural.
	Urban	Respondent’s type of place of residence is urban.
Division	Dhaka	Respondent lives in the Dhaka division
	Barisal	Respondent lives in the Barisal division
	Chattogram	Respondent lives in the Chattogram division
	Khulna	Respondent lives in the Khulna division
	Mymensingh	Respondent lives in the Mymensingh division.
	Rajhshahi	Respondent lives in the Rajshahi division
	Rangpur	Respondent lives in the Rangpur division.
	Sylhet	Respondent lives in the Sylhet division.

### 2.4 Statistical analyses

Univariate analysis was conducted to examine the sample characteristics. The chi-square (χ²) test was used to assess associations between the outcome variable and explanatory variables. A spatial map was constructed to examine the relationship between sanitary napkin usage and regional wealth. Districts with more than 60% individuals in the top three wealth quintiles were defined as high-wealth districts, while those at or below 60% were categorized as low-to-moderate wealth districts. These high-wealth areas were overlaid onto the prevalence map using a red dashed hatching pattern generated via the *ggpattern* package in R.

Additionally, to assess the bi-variate association between the outcome and subgroups created by education and wealth group, we have incorporated a binary logistic regression by using secondary or above education and bottom wealth as the reference group. The outcome of interest, use of sanitary napkins, was then modeled using a generalized linear model (GLM) with a logit link function to estimate adjusted odds ratios (AORs) for the study factors. After fitting the model, the marginal probabilities for six groups, defined by combinations of wealth index and individual education level, were estimated using the margins command in Stata (Stata SE V.14, StataCorp).

All analyses accounted for survey weights that were provided in the dataset to ensure representativeness. The spatial map was constructed using R (version 4.1.1). The district-level shapefile of Bangladesh was accessed from the *bangladesh* package in R [37], which provides open-source geographic boundary data*.* Maps were generated using the *ggplot2* package (version 4.3.2) without the use of proprietary basemap services such as Google Maps, Google Earth, or Google Street View.

### 2.5 Ethics approval and consent to participate

The Bangladesh MICS 2019 provides de-identified data, which cannot be used to identify any study participants. Regarding the ethics approval, the Bangladesh MICS 2019 protocol was approved by the technical committee of the Government of Bangladesh, led by the Bangladesh Bureau of Statistics (BBS). As the study utilized Bangladesh MICS 2019 as a secondary data source, it does not require additional ethical approval, nor does it have any information to identify study participants.

## 3 Results

Table 2 presents the demographic characteristics of 46,677 surveyed women aged 15–49 years. Notably, a predominant age group falls within the range of 20–39 years, representing 18.62% of the total sample. Educational attainment reveals a substantial majority, with 60.91% of respondents possessing secondary or higher qualifications. Regarding media exposure, a significant portion, accounting for 57.33% of the sample, reported being exposed to media almost daily. Approximately one-fifth (21.58%) of respondents acknowledged having a disability. Socioeconomic status demonstrates a notable disparity, with 35.41% of individuals falling within the bottom two wealth quantiles and 64.59% within the upper three. An examination of educational attainment within wealth indices reveals intriguing variations. Religious demographics indicate a Muslim majority, comprising 90.24% of the sample, while male-headed households constitute 88.23%. Notably, 72.81% of the surveyed women reported

**Table 2:** Socio-demographic characteristics of the study sample, Bangladesh MICS 2019.

	Distribution	
	n (unweighted)	% (weighted)
**Total**	46,677	100
**Age group**		
15-19	4,462	9.56
20-24	8,690	18.62
25-29	8,453	18.11
30-34	8,712	18.66
35-39	7,854	16.83
40-44	5,300	11.35
45-49	3,205	6.87
**Educational attainment**		
Pre-primary or none	7,209	15.44
Primary	11,039	23.65
Secondary or above	28,429	60.91
**Media exposure**		
Not at all	13,767	29.49
Less than once a week	2,233	4.78
At least once a week	3,914	8.39
Almost every day	26,762	57.33
**Disability**		
Not disable	36,593	78.40
Disable	10,075	21.58
**Wealth index**		
Bottom two	16,530	35.41
Upper three	30,147	64.59
**Educational attainment**		
Pre-primary or none	14,503	31.07
Primary	12,728	27.27
Secondary or above	19,445	41.66
**WI** **& Educational attainment**		
Pre-primary or none & Bottom two	4,278	9.17
Pre-primary or none & Upper Three	2,931	6.28
Primary & Bottom two	5,444	11.66
Primary & Upper Three	5,595	11.99
Secondary & Bottom two	6,808	14.59
Secondary & Upper three	21,621	46.32
**Religious affiliation**		
Other Religion	4,556	9.76
Muslim	42,121	90.24
**Sex of household head**		
Female	5,492	11.77
Male	41,184	88.23
**Community prevalence of sanitary napkin use**		
Low prevalence	33,821	72.81
Moderate prevalence	10,188	21.93
High prevalence	2,444	5.26
**Area of residence**		
Rural	35,245	75.51
Urban	11,432	24.49
**Division**		
Barisal	2,482	5.32
Chattogram	8,835	18.93
Dhaka	12,114	25.95
Khulna	5,526	11.84
Mymensingh	3,062	6.56
Rajshahi	6,262	13.42
Rangpur	4,966	10.64
Sylhet	3,429	7

using sanitary napkins, with the majority residing in rural areas (75.51%). Geographically, Dhaka emerges as the most represented division (25.95%), followed by Chattogram (18.93%).

The distribution of the proportion of women using sanitary napkins across districts in Bangladesh is illustrated in Fig 1. The spatial distribution reveals a generally poor level of sanitary napkin use throughout the country. In only five out of 64 districts does nearly one in three women use sanitary napkins, still a very low proportion. The northern region shows the lowest usage rates, with Lalmonirhat having the lowest proportion, where approximately 6% of women use sanitary napkin. Red dashed hatching highlights the 26 districts meeting the high-wealth threshold (>60% upper wealth quintiles). While elevated wealth does not consistently correlate to high adoption across all regions, low-usage districts mostly corresponded with poorer, non-high-wealth areas, particularly across the northern and northeastern belts.

**Fig 1:**
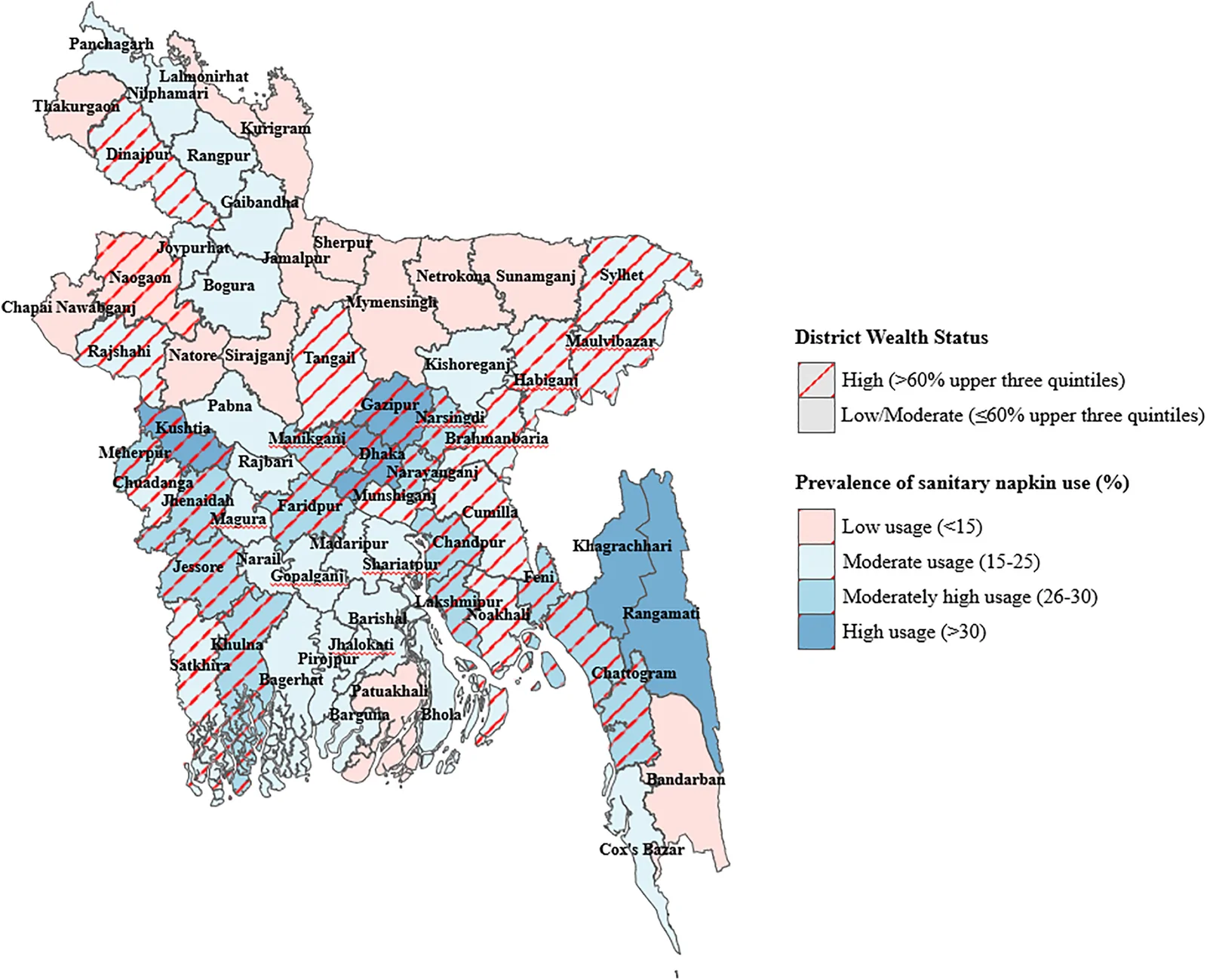
District-wise mapping on the distribution of sanitary napkin use and wealth status in Bangladesh. (Source of administrative boundaries: *bangladesh* R package (open-source shapefile data). Source of estimates: analysis from Bangladesh MICS data 2019.).

### 3.1 Sanitary napkin use by education and wealth

The overall prevalence of sanitary napkin usage among women aged 15–49 years was 23.4% (Supplementary table 1). Women with secondary or higher education from the bottom two wealth quintiles had a higher prevalence of sanitary napkin use (11.84%) than women from the upper three wealth quintiles who had pre-primary or no education (5.09%; p-value <0.05) or only primary education (10.82%; p-value = 0.14) (Fig 2).

**Fig 2:**
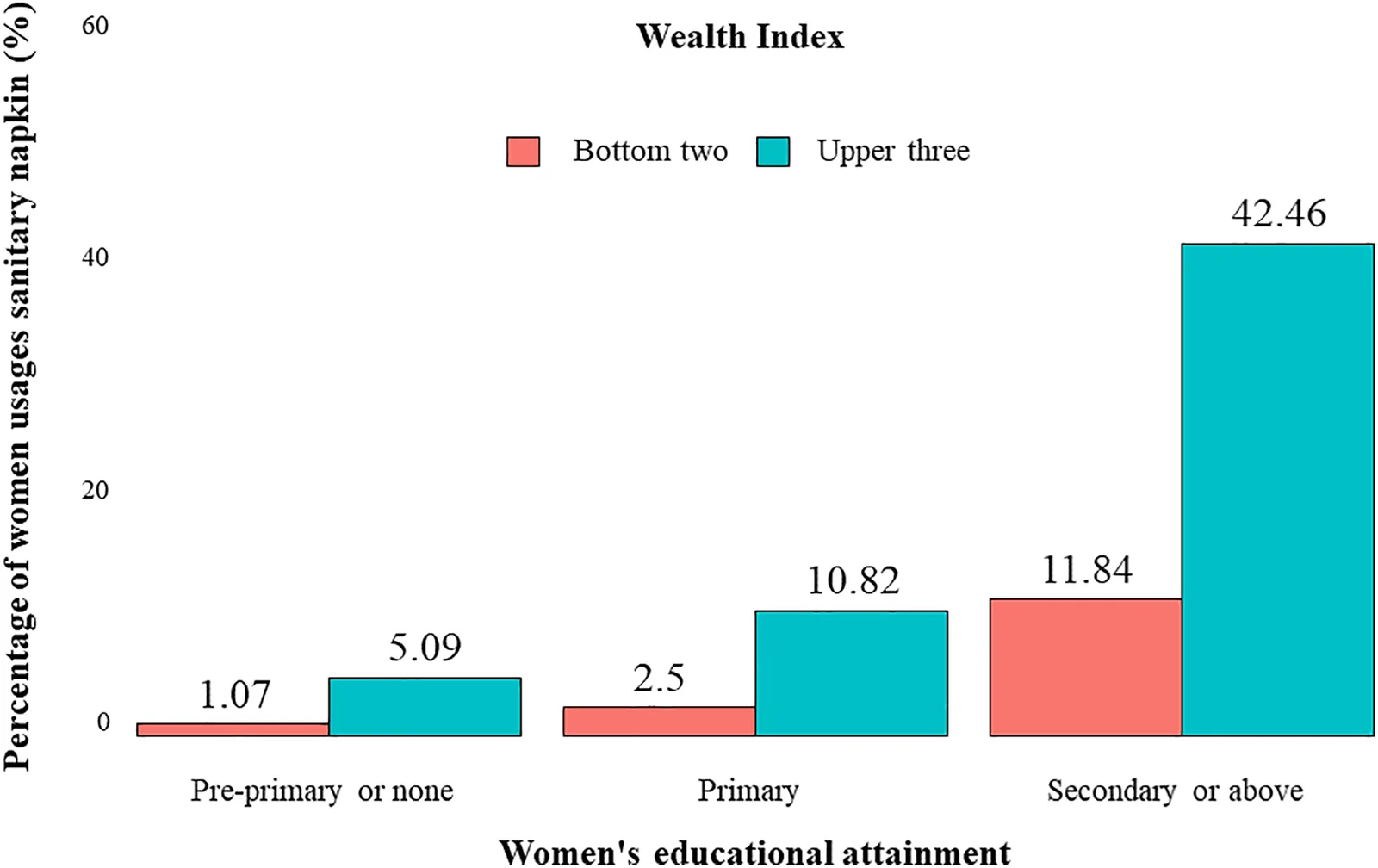
Percentage of women use sanitary napkins over educational attainment and wealth index among women age 15-49 years in Bangladesh, 2019.

Table 3 presents adjusted odds ratios (AOR) with associated p-values and 95% confidence intervals (CI) for the relationship between sanitary napking use and various demographic factors. Notable findings include a higher likelihood of using sanitary napkins in younger age groups, with substantial increases in odds ratios from 25–29 years (AOR = 2.57) to 15–19 years (AOR = 6.09). Education plays a significant role, with increasing AORs for primary (AOR = 1.44) and secondary or above (AOR = 4.5) compared to pre-primary or none. Media exposure shows a positive association, with higher odds for more frequent exposure. Individuals without disabilities have higher odds (AOR = 0.86) of using sanitary napkins. The wealth index indicates significantly higher odds (AOR = 2.3) in the upper three quantiles. Notable variations exist in the odds of sanitary napkin usage across different divisions and areas of residence, with urban residents, Dhaka division, and areas with high community prevalence having higher odds.

**Table 3:** Association of women’s education and wealth with sanitary napkin use among women aged 15-49 years in Bangladesh, 2019.

	AOR	p-value	95% CI	
			Lower	Upper
**Educational attainment**				
Pre-primary or none	1 (Ref.)			
Primary	1.44	0.05	1.0	2.05
Secondary or above	4.50	<0.01	3.28	6.16
**Wealth index**				
Bottom two	1 (Ref.)			
Upper three	2.30	<0.01	1.58	3.34
**Education &** **WI**				
Primary & Upper three	1.03	0.90	0.66	1.59
Secondary or above & Upper three	1.33	0.14	0.91	1.95
**Age**				
45-49	1 (Ref.)			
40-44	1.08	0.39	0.90	1.29
35-39	1.34	<0.01	1.14	1.58
30-34	1.54	<0.01	1.30	1.83
25-29	2.57	<0.01	2.18	3.03
20-24	4.17	<0.01	3.54	4.92
15-19	6.09	<0.01	5.13	7.23
**Media exposure**				
Not at all	1 (Ref.)			
Less than once a week	1.27	<0.01	1.08	1.51
At least once a week	1.42	<0.01	1.25	1.62
Almost every day	1.93	<0.01	1.76	2.11
**Disability**				
Not disable	1 (Ref.)			
Disable	0.86	<0.01	0.80	0.93
**Educational attainment of household head**				
Pre-primary or none	1 (Ref.)			
Primary	1.12	0.03	1.01	1.23
Secondary or above	2.11	<0.01	1.92	2.30
**Religious affiliation**				
Muslim	1 (Ref.)			
Other Religion	1.04	0.454	0.93	1.17
**Sex of Household head**				
Female	1 (Ref.)			
Male	0.84	<0.01	0.76	0.93
**Community prevalence of sanitary napkin usage**				
Low Use	1 (Ref.)			
Moderate Use	1.80	<0.01	1.59	2.03
High Use	3.75	<0.01	3.06	4.59
**Area of residence**				
Rural	1 (Ref.)			
Urban	1.50	<0.01	1.38	1.64
**Division**				
Dhaka	1 (Ref.)			
Barishal	1.15	0.02	1.02	1.29
Chattogram	0.74	<0.01	0.68	0.80
Khulna	0.92	0.06	0.84	1.01
Mymensingh	0.60	<0.01	0.50	0.71
Rajshahi	0.68	<0.01	0.62	0.76
Rangpur	0.67	<0.01	0.60	0.75
Sylhet	0.85	0.05	0.73	1.00
**Intercept**	0.01	<0.01	0.00	0.01

From the fitted regression model, we have calculated the marginal probabilities of sanitary napkin usage for every subgroup created by two variables: educational attainment and wealth quintiles. The national estimates from Fig 3 shows that, educational attainment significantly increases the likelihood of using sanitary napking among women aged 15–49 years in Bangladesh. Women with secondary or higher educational attainment from poorer households (bottom two quintiles) had a higher chance of using sanitary napkin compared to women with pre-primary or no educational attainment from a rich household (upper three). This behavior was consistent across all divisions.

**Fig 3:**
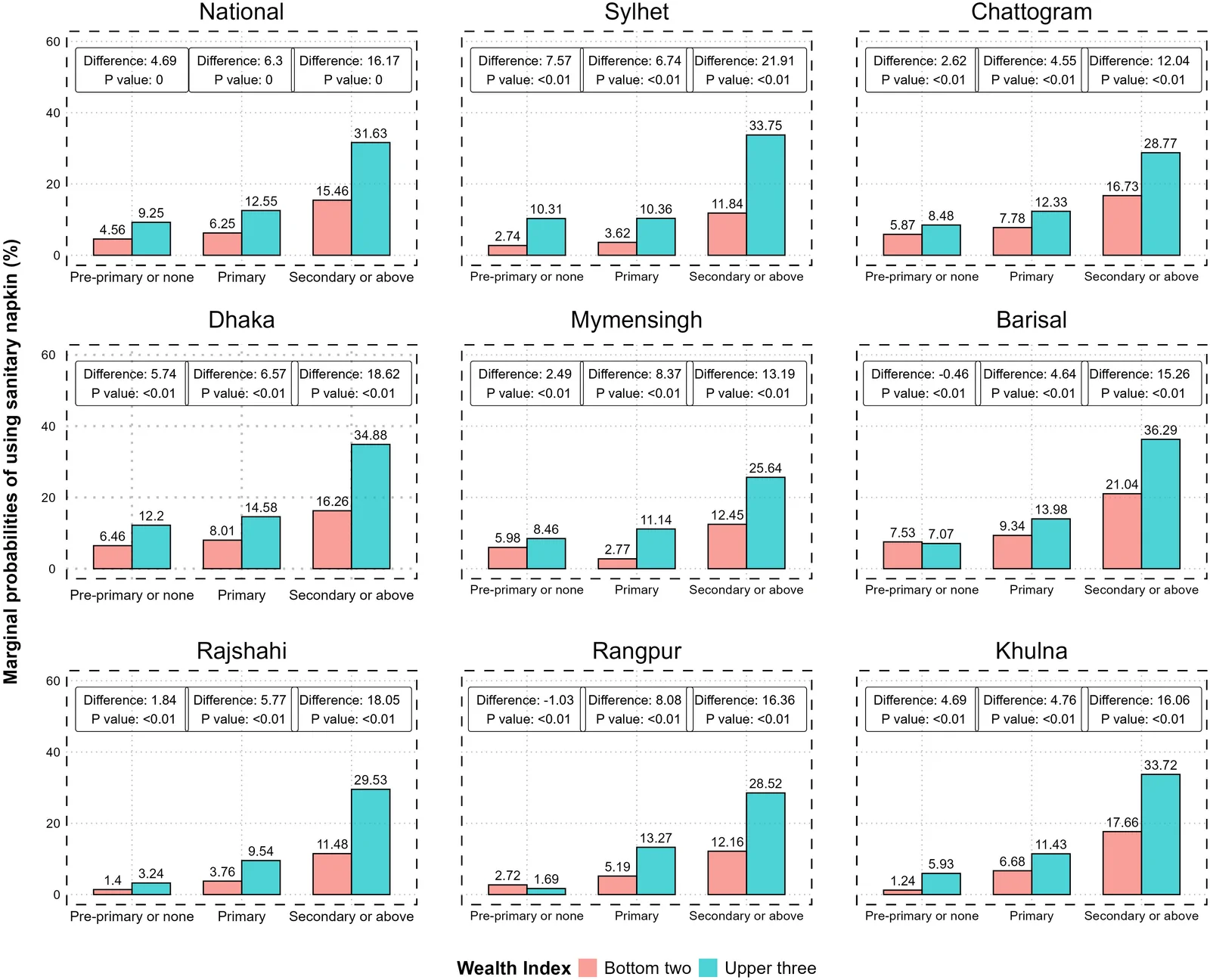
Marginal probabilities of sanitary napkin usage by women’s education and wealth among women aged 15-49 years in Bangladesh, 2019.

#### p-value from t-test.

## 4 Discussion

This study highlights the significant roles of both education and wealth in determining sanitary napkin use among women in Bangladesh. The results suggest that education has a particularly strong association with sanitary napkin use compared to wealth. Besides less educated, younger, disabled women, those living in male-headed or less educated households, with limited media exposure, residing in rural areas or in divisions like Mymensingh and Rangpur, and living in communities with low sanitary napkin use were less likely to use sanitary napkins.

### 4.1 Factors influencing sanitary napkin use

Consistent with prior research, this study identified significant variation in sanitary napkin use across geographic, household, and individual levels [38]. Findings indicates there are significant differences in sanitary napkin use across divisions, urban and rural areas, which are in line with previous studies [17]. This difference is might be due to some unobserved factors, which vary across regions.

At the community-level, prevalence of sanitary napkin use within a community was a significant predictor of individual use, reflecting the role of descriptive norms in shaping health behavior, which is consistent with Social Cognitive Theory [39]. In Bangladesh, where menstruation remain culturally stigmatized, communities with higher sanitary napkin use likely foster greater openness and awareness, reducing the social cost of adoption. Similar patterns have been observed elsewhere: in India, Sood et al. found that positive social norms significantly improved MHM [40]; in Nepal, Shrestha et al. reported that caste-based taboos limited awareness regarding MHM [41].

At the household-level, women in male-headed households were less likely to use sanitary napkins, consistent with evidence that household gender dynamics and educational level of the household head influence women’s health knowledge, autonomy, and access to resources [26]. From the perspective of the Andersen Behavioral Model [42], this finding indicates that household social structure may influence women’s access to and use of sanitary napkins.

At the individual-level mass media exposure seem to have a significant role in sanitary napkin use which serves as a core source of information on MHM [34]. Women who are exposed to media more frequently are more likely to have better knowledge and use of sanitary products, with those accessing media almost every day showing the highest likelihood.

The intersection of menstruation and disability endangers an intricate set of circumstances that can give rise to compounded discrimination and present distinct challenges for menstruating individuals with disabilities [24]. Our study supports this phenomenon, as women with disabilities were significantly less likely to use sanitary napkins. They face unique physical and structural obstacles, including inaccessible sanitation facilities, reliance on primary caregivers who may lack MHM awareness, and disability-related healthcare costs that lead families to deprioritize commercial hygiene products [43]. Addressing these barriers requires disability-inclusive WASH infrastructure, caregiver orientation, and targeted product assistance.

### 4.2 Interplay between education and wealth in napkin use

Our findings reveal that both education and wealth significantly influence the use of sanitary napkins, underscoring their interdependent roles in ensuring effective MHM. While higher levels of education are strongly associated with increased use, this effect is amplified in the presence of household wealth, indicating a synergistic relationship between the two factors.

Education enhances knowledge of menstruation as a biological process, reduces stigma, and improves attitudes towards the use of hygienic products [44]. Due to lack of awareness, menstrual health is often deprioritized and menstrual products are overlooked in basic WASH and sanitation planning [45]. However, even with increased education and wealth, many girls still refrain from using them due to embarrassment and stigma. A cross-sectional survey of adolescent girls in Rajshahi revealed that, factors like embarrassment purchasing pads from male shopkeepers and stigma surrounding menstruation were major barriers to sanitary napkin use [46]. This suggests that socio-cultural taboos pose further barriers to access that persist even when informational and economic constraints are addressed. This aligns with qualitative evidence on menstrual health equity, which demonstrates that overcoming such social barriers requires directly addressing underlying stigma and the lived experiences of young menstruators [47].

However, knowledge alone does not always translate into practice when economic barriers remain. This is particularly relevant in low-income households where affordability and availability are the key barriers [48]. In such settings, even when girls are aware of the benefits of sanitary napkins, they may be forced to rely on unsafe alternatives due to cost constraints.

### 4.3 Action taken to promote sanitary napkins

In Bangladesh, the government recognizes MHM as essential for women’s and girls’ health and has prioritized awareness and affordability in the National MHM Strategy 2021 [49]. To reduce costs and promote local manufacturing, the government has waived VAT on raw materials for sanitary napkin production. This move has decreased production costs but has not translated into lowered costs at the consumer level; a 15% VAT on finished sanitary napkin products still impedes uptake, especially in lower income households. The National MHM Coordination Committee has been established to oversee and align MHM efforts across health, education, and social sectors. Advocacy efforts can include eliminating the “pink tax” or VAT at the consumer level, improving distribution networks to ensure access in workplaces, schools, healthcare centers, and public spaces, alongside expanding MHM awareness sessions to marginalized and hard-to-reach communities. In other words, the first priority is reducing the cost across all populations, and second, enhancing acceptability and accessibility. This aligns with official position statements on eliminating period poverty [50] and clinical policy frameworks for advancing menstrual equity [51].

Notably, the current NCTB curriculum (after the recent update in 2025) does not adequately address menstruation and menstrual hygiene management, particularly in the subject “Physical Education and Health” for classes 6, 7, and 8 [52–54]. These textbooks lack essential information on menstruation, the use of sanitary napkins, and guidance on changing intervals, which are critical for maintaining good menstrual health. As a result, many adolescent girls remain uninformed or misinformed about safe and hygienic practices during menstruation. Revising the school curriculum in Bangladesh is imperative, as structured school-based educational initiatives are proven to enhance student knowledge [53]. Furthermore, broader evidence confirms that improving menstrual health literacy directly empowers young women to adopt better management strategies [55,56].

On the other hand, non-governmental research organizations have implemented programs to increase awareness about menstruation. One such initiative was the Ritu Programme, funded by the Embassy of the Kingdom of the Netherlands in Bangladesh and implemented from November 2015 to March 2020. The programme aimed to improve menstrual health among girls aged 10–15 in Netrokona, Bangladesh [57]. Through evidence-informed interventions, including school-based menstrual health education, community engagement with parents, and a national-level mass media campaign, the programme significantly boosted menstruation knowledge and management. It notably increased girls’ confidence and understanding of menstruation, improved hygiene practices (like changing materials more frequently and increased sanitary pad use).

Taken together, the findings underscore that MHM is shaped by a complex interplay of structural, social, and individual determinants. While individual education and household wealth are critical, they do not operate in isolation; rather, they are embedded within broader contextual factors such as community norms, media exposure, educational content, and policy frameworks. Therefore, effective menstrual health interventions must adopt a multi-level approach that simultaneously targets these interconnected domains.

### 4.4 Strengths and limitations of the study

A key strength of this study is the use of nationally representative MICS 2019 data, which ensures broad generalizability. It applies a generalized linear model to estimate adjusted associations and calculates marginal probabilities to clearly quantify the combined effects of education and wealth index on sanitary napkin use. By directly comparing subgroups (e.g., educated-poor vs. uneducated-rich), this study provides concrete evidence on the relative influence of education and wealth, offering actionable insights for targeted MHM interventions.

This study has several limitations. First, the cross-sectional nature of the data limits the scope of estimating the causal effects. Second, although we adjusted for major socio-demographic covariates, the potential for residual confounding remains from unmeasured factors such as cultural beliefs, familial traditions, or regional product availability. Third, self-reported data on sanitary napkin use may be subject to social desirability or recall bias. Lastly, certain contextual factors such as cultural norms, school-based education on menstruation, and access to WASH (Water, Sanitation, and Hygiene) facilities were not available in the dataset and could not be accounted for in the analysis. Another limitation is that the survey included only women aged 15–49 years, excluding younger adolescents who also menstruate and may have different menstrual health needs and product usage patterns.

## 5 Conclusion

This study demonstrates that menstrual health is influenced by interconnected factors at the individual, household, and community levels. Education and wealth showed significant interaction effects, with education emerging as a key driver of sanitary napkin use across wealth quintiles. However, education alone is unlikely to eliminate socioeconomic disparities in sanitary napkin use, as affordability remains an important barrier, particularly among women from poorer households. Therefore, standalone economic or informational campaigns may be incomplete. To be actionable, policymakers should implement integrated menstrual health interventions that combine comprehensive reproductive health literacy with measures to improve the affordability and accessibility of sanitary napkins, including reducing taxes and other costs that contribute to consumer prices. These strategies are vital for reducing sub-national disparities and ensuring sustainable, safe menstrual health practices across diverse socio-economic contexts in Bangladesh.

## Supporting information

S1 Table Distribution of sanitary napkin usage by sociodemographic variables among women aged 15-49 years in Bangladesh, 2019. (DOCX)
