# Supplementary material for: The combined role of education and wealth in menstrual health: Evidence from Bangladesh
Source: PLoS One. 2026 Oct 5;21(10):e0346457. doi: 10.1371/journal.pone.0346457 (PMC13637891; doi:10.1371/journal.pone.0346457)
Supplement: Supplementary file 6 [file pone.0346457.s008.pdf]

Rebuttal letter

Reviewer #2: I appreciate the title change, which is more consistent with the article's content.

I agree with a prior reviewer's use of "menstrual health" rather than "menstrual hygiene", with the latter being more stigmatizing and sending an implicit message of judgement or blame on the person menstruating.

Comment 1: Please go through the document and change "menstrual hygiene" to "menstrual health."

Response 1: Thank you for this suggestion. We respectfully retained the term "menstrual hygiene" because our outcome variable specifically measures sanitary napkin use, which reflects a menstrual hygiene practice rather than menstrual health itself. We therefore consider "menstrual hygiene" to be the more appropriate term for describing the outcome and its interpretation in this study.

Comment 2: In the opening paragraph, I might make this more explicit: "To address this gap, this study examines the interplay between education and wealth in determining the use of sanitary napkins in Bangladesh, with sanitary napkin use as a proxy for menstrual health. This choice reflects the predominant usage of sanitary napkins for menstrual management in Bangladesh."

Making it more clear that use of sanitary napkins may represent a measure of wealth rather than a measure of menstrual health literacy would be useful as an opening hypothesis worthy of analysis.

Response 2: We have added the suggested text to the opening paragraph (lines 18-20).

Comment 3: Figure 1 would be better if it also had markings (eg diagonal lines in a printed fashion) in areas of wealth, so that we can see at a glance if the high usage areas corresponded with top tier wealth.

Response 3: We respectfully chose not to add wealth overlays to Figure 1, as including additional layers could create visual clutter and reduce the clarity of the map. The relationship between wealth quintiles and sanitary napkin use is presented in greater detail in Figures 2 and 3.

Comment 4:

P9 line 42- would not capitalize menarche: "(aka Menarche)"

P9 line 45- "between the ages of 12 to 15 years"

P9 line 56: Spell out RTI and then put initials in parentheses

Response 4: All three minor corrections have been made as suggested.

Comment 5: P11 line 114. It would be useful to include a power analysis to ascertain that 20 households per group would represent potential significance.

Response 5: Thank you for the feedback. As this study used secondary data from the nationally representative 2019 Bangladesh MICS, the sample size was determined by the survey design. Also, the detail power analysis is presented in the 2019 Bangladesh MICS report.

Comment 6: P17 line 193-195: need p values here.

Response 6: We included p-values from a binary logistic regression model evaluating the combined wealth–education subgroups, treating the "Secondary or above & Bottom two wealth" group as the reference category.

Comment 7: P 20 line 220: correct "This behavibiour"- spellcheck please

Response 7: Thank you for pointing this out. We have corrected the spelling.

Comment 8: p 21 lines 246-250 in the discussion paragraph: Did this vary by income level? Did patriarchal high income households still have less sanitary napkin use than matriarchal high income households? How did educational level impact this finding?

Response 8: While an important query, evaluating multi-way interaction effects between household headship, wealth, and education was beyond the defined scope of this study. We have noted this as a key area for future research.

Comment 9: P 21 line 251- did you mean "seem to have a significant role" rather than "seen to have a significant role"?

Response 9: Thank you for pointing this out. We have corrected the spelling.

Comment 10: P 21 paragraph 251-257- the jump from mass media exposure to menstruation and disability is abrupt with no transition. Are women who are exposed to more mass media more or less likely to be of higher educational background? How does that relate to income? The sentences on disability should be in their own paragraph and have more discussion around menstrual health findings for those with disabilities.

Response 10: We have fixed the transition. Regarding the effect of mass media, our analysis was not focused on assessing its interaction effect with education, as a result current analysis can not answer that particular question.

Additionally, we have deepened the argument regarding the disability as suggest by the reviewer.

Comment 11: P22- line 273-273. Should be are, not is: “This is particularly relevant in low-income households where affordability and availability are the key barriers”

Response 11: Thank you for pointing this out. We have corrected it.

Comment 12: Line 279 and following paragraph. “To reduce costs and promote local manufacturing, the government has waived VAT on raw materials for sanitary napkin production. This move has decreased production costs but has not translated into lowered costs at the consumer level; a 15% VAT on finished sanitary napkin products still impedes uptake, especially in lower income households. The National MHM Coordination Committee has been established to oversee and align MHM efforts across health, education, and social sectors. Advocacy efforts can include eliminating the “pink tax” or VAT at the consumer level, improving distribution networks to ensure access in workplaces, schools, healthcare centers, and public spaces, alongside expanding MHM awareness sessions to marginalized and hard-to-reach communities. In other words, the first priority is reducing the cost across all populations, and second, enhancing acceptability and accessibility.”

Response 12: We have replaced the paragraph with the provided text.

Comment 13: P330- Conlusion: should be far more clear on the need to eliminate the “pink tax” on consumer products for menstrual health, specifically sanitary napkins in India but also other menstrual products. Education alone will not suffice; affordability appears to be a significant barrier. Reducing VAT on raw materials clearly does not do enough to lower consumer costs.

Response 13: We updated the conclusion to highlight that education alone is insufficient and that eliminating consumer-level taxes on finished menstrual products is essential for overcoming affordability barriers in Bangladesh.

Comment 14: It also appears that the authors did not incorporate any of this reviewer’s prior recommendations, including addition of any of these articles to the discussion:

Mittermeier, T · Hawkey, A · Bush, D et al. Menstrual health symptoms and literacy among young women in Aotearoa New Zealand: a nationwide cross-sectional survey. J Pediatr Adolesc Gynecol. 2025; 38:328-335

Bhoda, K · Burgoa, S · Zerrouki, Y ...Examining Menstrual Health and Hygiene Educational Initiatives for American Middle and High School Menstruating Students: A Scoping Review. J Pediatr Adolesc Gynec. 2024; 37(5):465-476

Harrison, ME · Davies, S · Tyson, N ...NASPAG Position Statement: Eliminating Period Poverty in Adolescents and Young Adults Living in North America. J Pediatr Adolesc Gynecol. 2022; 35(6):609-611

Davies, S · Darien, K · Bazyar, F ...A Multimodal Qualitative Approach to Understanding Menstrual Health Equity among Adolescents and Young Adults. Journal of Pediatric and Adolescent Gynecology. 2023;

36(6):511-517

Armour M, Hyman MK et al. Menstrual Health Literacy and Management Strategies in Young Women in Australia: A National Online Survey of Young Women Aged 13-25 Years. Journal of Pediatric and Adolescent GynecologyVol. 34Issue 2p135–143Published online: November 11, 2020

Rome ES, Tyson N. Menstrual Equity. Obstet Gynecol Clin North Am. 2024 Dec;51(4):731-744. doi: 10.1016/j.ogc.2024.08.008. Epub 2024 Sep 21

Response 14: We thank the reviewer for suggesting these important materials. We have carefully read and added these as reference where appropriate.
