# Supplementary material for: The combined role of education and wealth in menstrual health: Evidence from Bangladesh
Source: PLoS One. 2026 Oct 5;21(10):e0346457. doi: 10.1371/journal.pone.0346457 (PMC13637891; doi:10.1371/journal.pone.0346457)
Supplement: Supplementary file 7 [file pone.0346457.s002.pdf]

Manuscript ID: PONE-D-26-13387
Title: Awareness or Affordability: What matters the most in ensuring menstrual hygiene management?

Overall Evaluation

This manuscript addresses a highly relevant public health issue, menstrual hygiene management (MHM) by examining the relative and combined influence of awareness and affordability on sanitary napkin use in Bangladesh. The use of nationally representative MICS data and application of regression and marginal probability analyses are notable strengths. The study provides valuable insights for policy and program design in low-resource settings. However, despite its strengths, the manuscript requires major revision due to conceptual oversimplification, methodological limitations (particularly proxy measurement), insufficient critical interpretation, and language inconsistencies. Strengthening these areas will significantly enhance the rigor and contribution of the study.

Title and Abstract

The title is clear, engaging, and directly reflects the core research question, effectively highlighting the comparison between awareness and affordability. The abstract is well-structured and summarizes the background, methods, findings, and conclusions. However, it remains largely descriptive and lacks emphasis on novelty and theoretical contribution. The use of education and wealth index as proxies for awareness and affordability, respectively, is mentioned but not critically justified in the abstract. Additionally, the conclusion may slightly overstate the causal implication, given the cross-sectional design. The abstract would benefit from a clearer statement of the research gap and a more cautious interpretation of findings.

Introduction

The introduction provides a comprehensive overview of menstruation, MHM, and its global and national significance. It effectively contextualizes the issue within Bangladesh using relevant statistics and literature. The discussion of stigma, health outcomes, and social implications is appropriate and well-supported. Importantly, the authors identify a research gap regarding the interaction between awareness and affordability, which is a strong aspect of the manuscript.

However, the section could be improved by tightening its structure and reducing redundancy. Some paragraphs are overly descriptive and could be more concise. Additionally, while the research gap is mentioned, it could be articulated more sharply to emphasize the novelty of the interaction analysis. The conceptual distinction between awareness (education) and affordability (wealth index) should also be more critically justified, as these proxies may not fully capture the constructs.

Methods

The methods section is generally well-organized and provides sufficient detail about the data source, sampling design, and analytical approach. The use of nationally representative MICS data strengthens the external validity of the findings. The application of generalized linear models and marginal probability estimation is appropriate and enhances interpretability.

However, there are several important limitations. First, the study relies on proxy variables, education for awareness and wealth index for affordability which may not accurately reflect the intended constructs. This introduces potential measurement bias and should be more critically discussed. Second, the cross-sectional design limits causal inference, yet the manuscript occasionally implies causality. Third, although survey weights are applied, there is limited discussion on handling potential confounding or multicollinearity. Finally, the binary classification of sanitary napkin use as a proxy for "proper MHM" may oversimplify menstrual practices, as other safe alternatives (e.g., menstrual cups) are not considered.

Results

The results are clearly presented, with appropriate use of tables, figures, and statistical estimates. The descriptive statistics provide a comprehensive overview of the sample characteristics. The regression analysis and adjusted odds ratios are well reported, and the inclusion of marginal probabilities is a strong addition that improves interpretability.

The spatial analysis (district-level mapping) adds value by highlighting geographic disparities. Figures (e.g., Fig 1 and Fig 2) effectively illustrate differences in sanitary napkin use across regions and socioeconomic groups. The finding that education (awareness) may outweigh wealth (affordability) in certain contexts is particularly interesting.

However, the results section is somewhat descriptive and could benefit from clearer emphasis on key findings. Additionally, some interpretations are presented within the results rather than reserved for the discussion. Greater clarity in linking statistical findings to the research question would improve this section.

Discussion

The discussion appropriately interprets the findings and situates them within existing literature. The emphasis on the interaction between awareness and affordability is a key strength, and the discussion of community, household, and individual-level factors provides a multi-level perspective.

However, the discussion remains somewhat descriptive and lacks deeper critical analysis. The manuscript would benefit from stronger theoretical framing, for example by linking findings to behavior change theories or health access models. While policy implications are mentioned, they

are not sufficiently developed or specific. The claim that awareness has a stronger effect than affordability should be interpreted more cautiously, considering the limitations of proxy variables.

The section on policy and program interventions (e.g., VAT policy, curriculum gaps, and Ritu program) is informative but could be more directly linked to the study findings. Additionally, there is some repetition across paragraphs, and the overall structure could be more concise.

Strengths and Limitations

The authors acknowledge several strengths, including the use of nationally representative data and advanced statistical methods. The discussion of limitations is appropriate, particularly regarding the cross-sectional design and use of proxy variables.

However, this section could be expanded further. For instance, the potential for residual confounding, measurement error, and reporting bias should be more explicitly discussed. The limitation regarding the binary outcome variable and exclusion of other menstrual products should also be highlighted more clearly.

Conclusion

The conclusion effectively summarizes the main findings and reinforces the importance of addressing both awareness and affordability. The recommendation for integrated interventions is appropriate and aligns with the study results.

However, the conclusion could be strengthened by providing more specific and actionable policy recommendations. Additionally, the language should be slightly moderated to avoid implying causality, given the observational nature of the study.

References

The reference list is comprehensive and includes relevant and recent literature. The citations are generally appropriate and support the arguments made in the manuscript. However, minor inconsistencies in formatting should be corrected to ensure compliance with journal guidelines.

Language and Writing Quality

The manuscript is generally well-written and understandable. However, there are several grammatical errors, typographical issues, and inconsistencies in terminology. Some sentences are overly long or repetitive. Professional language editing is recommended to improve clarity and readability.
