# Supplementary material for: The combined role of education and wealth in menstrual health: Evidence from Bangladesh
Source: PLoS One. 2026 Oct 5;21(10):e0346457. doi: 10.1371/journal.pone.0346457 (PMC13637891; doi:10.1371/journal.pone.0346457)
Supplement: Supplementary file 10 [file pone.0346457.s006.pdf]

Awareness or Affordability: What matters the most in ensuring menstrual hygiene management?

Rebuttal Letter

Reviewer 03.

Comment 1 (*line 17: awareness regarding menstrual hygiene*)**:** This will require a more detailed definition in the paper -- awareness of menstrual hygiene from a health/biological standpoint? Management of menstrual hygiene/product use? Options for products? What do we mean by awareness here?

Response 1: We have now clear out what do we meant by awareness here (knowledge regarding the menstrual hygiene management) and also broaden the explanation in the explanatory variables part (lines 131).

Comment 2 (*line 23: individual educational attainment*)**:** I'd be curious to see more justifications around the selection of this proxy -- e.g. some sort of evidence/justification around school/educational facilities being the critical entry point to awareness around menstrual products.

Response 2: We thank the reviewer for this important comment. We have justified the use of this proxy in the explanatory variable part (lines 131-134).

Comment 3 (*line 31: sanitary napkins*)**:** Were only sanitary napkins assessed? Or was this meant to be inclusive of the full range of menstrual products?

Response 3: The term "sanitary napkins" refers to reported use of sanitary pads during the last menstrual period, rather than assessment of a full range of individual product types separately (please see the updated outcome variable section; lines 122-125).

Comment 4 (*line 58: This poor management of menstrual hygiene*)**:** Suggest re-framing to a way that doesn't place blame on the person who menstruates -- there has largely been a shift away from framing around 'menstrual hygiene' and towards 'menstrual health' for this reason. In this framing, it sounds like the person who menstruates is to blame for these health outcomes, rather than the system that has failed them.

Response 4: We thank the reviewer for this important suggestion. We have revised the text accordingly (lines 56-57).

Comment 5 (*line 81-82: only a quarter of adolescent girls know about menstruation before their menarche*)**:** This seems to suggest that menstrual health is not taught in schools, which feels counter to the proxy indicator of educational status to indicate awareness about menstrual health/menstrual products.

Response 5: We thank the reviewer for this sharp observation. We agree that the low rate of pre-menarche knowledge seems contradictory at first glance. This statistic reflects a well-documented reality in Bangladesh: reproductive health topics are often introduced too late in the curriculum (or skipped altogether in primary school), leaving young girls uninformed at the actual onset of menarche. However, our proxy of "educational attainment" for women aged 15–49 measures their cumulative exposure to schooling. As women progress into secondary and higher education, they gain delayed but crucial exposure to health curricula. Thus, while early school fails to prepare them before menarche, longer retention in the education system is still the primary driver of MHM awareness.

Comment 6 (*line 117: The survey collected detailed information from women of reproductive age (15–49 years)*)**:** Suggest acknowledging the gap on young adolescent data -- the average age of menarche is much younger than 15, which means that we aren't capturing the full menstruating population with these data. This is hard to address since MICS / survey data don't include this, but suggest highlighting this potential gap.

Response 6: We have added this as a limitation (lines 325-327).

Comment 7 (*line 125-127: In this study, sanitary napkin use was considered a proxy for proper menstrual hygiene, as they are typically cleaner, safer, and more absorbent than traditional or improvised materials*)**:** Better define what is meant by sanitary napkin and whether this includes both reusable and disposable options or just disposable/single use. Include context on whether cloth/non-purpose made materials were considered to 'count' as sanitary napkins in this definition.

Response 7: We thank the reviewer for this comment. Sanitary napkin use is defined as any reported use of purpose-made sanitary pads during the last menstrual period, regardless of exclusive or combined use (refer to the updated outcome variable section).

Comment 8 (*line 128*)**:** An additional variable that should be part of this analysis is actual availability of products -- availability itself is often a barrier and if sanitary napkins are not readily available in certain communities (most often, rural or vulnerable communities, areas with lower household wealth, etc), this creates a confounding variable that influences outcome. Suggest adding analysis on this that speaks to how readily available products are in these regions... beyond just actual use of products. Use of products and prevalence of community use still rely on products being available to purchase and use.

Response 8: We thank the reviewer for this point. Unfortunately, the 2019 Bangladesh MICS dataset does not contain variables on commercial market supply or physical product availability in communities.

Comment 9 (*line 135-137: Individual level factors that can affect use of sanitary napkin included participants age, educational attainment, exposure to media, and status of disability*)**:** Suggest acknowledging that you are missing a critical age group within these data, since the data begin at age 15 but average age of menarche is much lower. I would acknowledge this and contextualize a bit further.

Response 9: We have added this as a limitation (lines 325-327).

Comment 10 (*Table 1: The community prevalence of sanitary napkins use is below 30%*)**:** Contextualize what this includes. Can you also clarify how prevalence is defined here? Is it a simple yes/no on access to sanitary pads, or does it measure for *sufficient* access, e.g. sufficient product access for the full cycle, rather than supplementation with non-purpose made products (e.g. 1-4 sanitary pads per cycle, supplemented with cloth or other materials).

Response 10: We thank the reviewer for this comment. The community prevalence was built on to the binary outcome defined in the outcome variable section, where sanitary napkin use was measured as any reported use during the last menstrual period (yes/no), regardless of quantity or whether other materials were also used (lines 144-146).

Comment 11 (*lines 192-194: Notable findings include a higher likelihood of using sanitary napkins in younger age groups, with substantial increases in odds ratios from 25-29 years (AOR=2.57) to 15-19 years (AOR=6.09)*)**:** This seems counter to the findings that higher educational levels result in higher likelihood of use, as most women/girls with higher education are likely to be older than those with lower.

Response 11: We thank the reviewer for this important comment. The findings reflect two different effects estimated in the adjusted model. Age captures cohort differences in menstrual hygiene practices, where younger women are more likely to adopt sanitary napkins due to more recent exposure to menstrual health information and changing norms. Education, on the other hand, reflects individual awareness and knowledge, which increases the likelihood of sanitary napkin use across all age groups. Therefore, higher use among younger women and more educated women are not contradictory, but represent separate pathways influencing behavior.

Comment 12 (*lines 206-207: educational attainment significantly increases the likelihood of using sanitary napking among women aged 15-49 years in Bangladesh*)**:** I think that this is a more powerful finding than framing the findings around educational attainment as being a proxy for 'awareness.'

Response 12: We agree with the reviewer that formal educational attainment is a powerful determinant with broader societal implications beyond acting as a proxy for awareness. However, to keep the paper tightly aligned with our research question, our analysis and discussion focus specifically on its role in health awareness and information access.

Comment 13 (*lines 216-217: This study highlights the significant roles of both awareness and affordability in determining sanitary napkin use among women in Bangladesh*)**:** For this takeaway to be highlighted, I think that much more work is needed to do justify educational attainment as a proxy for awareness. I think that the stronger framing is that educational attainment is significant in determining sanitary napkin use -- and a potential policy takeaway/discussion lens could be unpacking how education might lead to increased awareness. I also think that we need much more contextual definition on what is meant by awareness here.

Response 13: We thank the reviewer for this observation. As detailed in our Explanatory Variables (lines 131-138) and Discussion sections (lines 256-259), we use formal education as an established proxy for health awareness because higher educational attainment is a primary structural pathway through which women access health literacy and information on menstrual hygiene.

Comment 14 (lines 225: Comparison with earlier studies)**:** Is this the right header here? This seems to be looking at other individual factors within the context of this study, rather than prior studies. Additional studies are woven in to this discussion, but suggest re-framing the header to focus more on the other individual factors as the focus of this section.

Response 14: We thank the reviewer for this helpful suggestion. We have revised the header to "Factors influencing sanitary napkin use".

Comment 15 (lines 232: proper hygiene practices.)**:** Avoid framing like 'proper' hygiene practices -- this shifts blame to the person menstruating rather than a system that has failed her.

Response 15: We thank the reviewer for this insightful comment. We agree that the term "proper" can inadvertently imply individual blame. We have revised the manuscript to replace "proper" with "recommended" to focus on the practices themselves rather than casting judgment on the individual.

Comment 16 (lines 255-258)**:** I think that this context needs to come much earlier in the paper to help readers understand what is meant by awareness -- and why education might be a proxy for it.

Response 16: We thank the reviewer for the suggestion. In the revised manuscript, we have broadened the rationale for using these proxies (lines 131-138).

Comment 17 (lines 258-261)**:** This next part seems to focus more on stigma/shame/taboo than on awareness. I would suggest either (1) expanding on why/how awareness reduces stigma/shame and therefore overcomes this; or (2) saying that even with increased awareness, stigma/shame pose further barriers to access.

Response 17: We thank the reviewer for this helpful suggestion. We have revised the Discussion (according to option 2) to clarify that (lines 262-264).

Comment 18 (lines 263: availability)**:** Did your statistical analysis take this into account at all? E.g. was there any analysis done on whether availability itself might have been why people weren't using sanitary napkins? These products tend to be less available in rural communities, lower-income areas, or less-educated communities -- could availability itself have been a confounding factor that wasn't explored?

Response 18: We thank the reviewer for this point. Unfortunately, the 2019 Bangladesh MICS dataset does not contain variables on commercial market supply or physical product availability in communities.

Comment 19 (lines 279-281: These textbooks lack essential information on menstruation, the use of sanitary napkins, and guidance on changing intervals, which are critical for maintaining good menstrual hygiene practices.)**:** This adds to my comment throughout questioning the proxy of education to awareness -- this seems to imply that education doesn't equate to awareness. Suggest addressing more directly this tension.

Response 19: We thank the reviewer for highlighting this apparent tension. There are two key points to this:

First, there is a chronological distinction. Our study utilizes 2019 MICS data, representing a period when the national curriculum **did** contain basic components of menstrual health. The omission of these critical components occurred during the recent 2025 national curriculum update. We have now clear out this in line 281.

Second, utilizing educational attainment as a proxy for MHM awareness does not strictly assume that MHM components must be present in the textbooks. Formal schooling builds general health literacy, provides the cognitive tools to process external health information, and fosters crucial peer-to-peer knowledge sharing among adolescent girls.

Comment 20 (lines 296-297: rather, they are embedded within broader contextual factors such as community norms, media exposure, educational content, and policy frameworks.)**:** And also market dynamics -- how available are products?

Response 20: We thank the reviewer for this important point. We acknowledge that market dynamics, including product availability and supply-side factors, are also relevant contextual determinants of menstrual hygiene management. However, as our dataset did not include information on product availability, we were unable to assess this aspect in the present study.

Comment 21 (lines 304: effects of education and wealth)**:** Suggest framing like this more consistently throughout -- rather than around 'awareness.'

Response 21: We thank the reviewer for pointing this out. We have now revised the terms to make them consistent.

Comment 22 (lines 309-311: Second, awareness and affordability were measured using proxy indicators—educational attainment and household wealth index—which may not fully capture the complexity of these constructs.): Suggest expansion on this one.

Response 22: We agree. We have expanded this limitation section to clarify that individual educational attainment does not directly capture specific menstrual health literacy, and the household wealth index does not necessarily reflect an individual woman's financial autonomy or direct purchasing power. Please refer to lines 315-321 in the revised manuscript.

Reviewer 01:

Comment 01: Overall Evaluation

This manuscript addresses a highly relevant public health issue, menstrual hygiene management (MHM) by examining the relative and combined influence of awareness and affordability on sanitary napkin use in Bangladesh. The use of nationally representative MICS data and application of regression and marginal probability analyses are notable strengths. The study provides valuable insights for policy and program design in low-resource settings. However, despite its strengths, the manuscript requires major revision due to conceptual oversimplification, methodological limitations (particularly proxy measurement), insufficient critical interpretation, and language inconsistencies. Strengthening these areas will significantly enhance the rigor and contribution of the study.

Response 01: We sincerely thank the reviewer for their encouraging assessment of our study's strengths and its relevance to public health. We have carefully considered this feedback and have addressed the concerns accordingly. Please find our detailed, point-by-point responses to each specific comment below.

Comment 02: Title and Abstract

The title is clear, engaging, and directly reflects the core research question, effectively highlighting the comparison between awareness and affordability. The abstract is well-structured and summarizes the background, methods, findings, and conclusions.

However, it remains largely descriptive and lacks emphasis on novelty and theoretical contribution. The use of education and wealth index as proxies for awareness and affordability, respectively, is mentioned but not critically justified in the abstract. Additionally, the conclusion may slightly overstate the causal implication, given the cross-sectional design. The abstract would benefit from a clearer statement of the research gap and a more cautious interpretation of findings.

Response 02: We thank the reviewer for this constructive feedback. We have revised the Abstract to explicitly state the research gap (the lack of studies exploring the interactive effects of awareness and affordability) and provided a brief justification for using education and wealth index as operational proxies.

Comment 03: Introduction

The introduction provides a comprehensive overview of menstruation, MHM, and its global and national significance. It effectively contextualizes the issue within Bangladesh using relevant statistics and literature. The discussion of stigma, health outcomes, and social implications is appropriate and well-supported. Importantly, the authors identify a research gap regarding the interaction between awareness and affordability, which is a strong aspect of the manuscript.

However, the section could be improved by tightening its structure and reducing redundancy. Some paragraphs are overly descriptive and could be more concise. Additionally, while the research gap is mentioned, it could be articulated more sharply to emphasize the novelty of the interaction analysis. The conceptual distinction between awareness (education) and affordability (wealth index) should also be more critically justified, as these proxies may not fully capture the constructs.

Response 03: We thank the reviewer for the positive evaluation. We have significantly tightened this section and sharpened the description of the research gap (lines 95-102). For the proxy, we have broaden the justification of using these indicators in the method section (lines 131-138).

Comment 04: Methods

The methods section is generally well-organized and provides sufficient detail about the data source, sampling design, and analytical approach. The use of nationally representative MICS data strengthens the external validity of the findings. The application of generalized linear models and marginal probability estimation is appropriate and enhances interpretability.

However, there are several important limitations. First, the study relies on proxy variables, education for awareness and wealth index for affordability which may not accurately reflect the intended constructs. This introduces potential measurement bias and should be more critically discussed. Second, the cross-sectional design limits causal inference, yet the manuscript occasionally implies causality. Third, although survey weights are applied, there is limited discussion on handling potential confounding or multicollinearity. Finally, the binary classification of sanitary napkin use as a proxy for "proper MHM" may oversimplify menstrual practices, as other safe alternatives (e.g., menstrual cups) are not considered.

Response 04: We thank the reviewer for confirming the appropriateness of our analytical approach. We completely agree with the identified limitations. As noted, because this study is a secondary analysis of survey data, these constraints are structural. We have fully acknowledged and critically discussed each of these points in the Strengths and Limitations section of the discussion.

Comment 05: Results

The results are clearly presented, with appropriate use of tables, figures, and statistical estimates. The descriptive statistics provide a comprehensive overview of the sample characteristics. The regression analysis and adjusted odds ratios are well reported, and the inclusion of marginal probabilities is a strong addition that improves interpretability. The spatial analysis (district-level mapping) adds value by highlighting geographic disparities. Figures (e.g., Fig 1 and Fig 2) effectively illustrate differences in sanitary napkin use across regions and socioeconomic groups. The finding that education (awareness) may outweigh wealth (affordability) in certain contexts is particularly interesting.

However, the results section is somewhat descriptive and could benefit from clearer emphasis on key findings. Additionally, some interpretations are presented within the results rather than reserved for the discussion. Greater clarity in linking statistical findings to the research question would improve this section.

Response 05: We thank the reviewer for the highly positive feedback on our presentation of the results. To address the concern regarding descriptive tone and interpretation, we have carefully re-reviewed the Results section to ensure that statistical findings are tied directly to our core research questions and that any explanatory interpretation is strictly reserved for the discussion section.

Comment 06: Discussion

The discussion appropriately interprets the findings and situates them within existing literature. The emphasis on the interaction between awareness and affordability is a key strength, and the discussion of community, household, and individual-level factors provides a multi-level perspective.

However, the discussion remains somewhat descriptive and lacks deeper critical analysis. The manuscript would benefit from stronger theoretical framing, for example by linking findings to behavior change theories or health access models. While policy implications are mentioned, they are not sufficiently developed or specific. The claim that awareness has a stronger effect than affordability should be interpreted more cautiously, considering the limitations of proxy variables.

The section on policy and program interventions (e.g., VAT policy, curriculum gaps, and Ritu program) is informative but could be more directly linked to the study findings. Additionally, there is some repetition across paragraphs, and the overall structure could be more concise.

Response 06: We have revised the Discussion to provide a more critical analysis. We integrated two theoretical frameworks, the Andersen Model of Health Services Use and Social Cognitive Theory, to better explain the multi-level factors shaping MHM behaviors. Per your suggestion, we now interpret the comparison between awareness and affordability more cautiously, acknowledging the limitations of our proxy variables. Finally, we directly linked policy interventions (VAT and curriculum gaps) to our empirical findings and streamlined the section to improve conciseness.

Comment 07: Strengths and Limitations

The authors acknowledge several strengths, including the use of nationally representative data and advanced statistical methods. The discussion of limitations is appropriate, particularly regarding the cross-sectional design and use of proxy variables.

However, this section could be expanded further. For instance, the potential for residual confounding, measurement error, and reporting bias should be more explicitly discussed. The limitation regarding the binary outcome variable and exclusion of other menstrual products should also be highlighted more clearly.

Response 07: We thank the reviewer for these excellent suggestions. We have expanded our study limitations section to explicitly discuss the potential impacts of residual confounding (lines 313-315) and proxy-related measurement error (315-321).

Comment 08: Conclusion

The conclusion effectively summarizes the main findings and reinforces the importance of addressing both awareness and affordability. The recommendation for integrated interventions is appropriate and aligns with the study results.

However, the conclusion could be strengthened by providing more specific and actionable policy recommendations. Additionally, the language should be slightly moderated to avoid implying causality, given the observational nature of the study.

Response 08: We thank the reviewer for this constructive feedback. We have revised the Conclusion to provide specific, actionable policy recommendations.

Comment 09: References

The reference list is comprehensive and includes relevant and recent literature. The citations are generally appropriate and support the arguments made in the manuscript. However, minor inconsistencies in formatting should be corrected to ensure compliance with journal guidelines.

Response 09: We appreciate the reviewer's comment. We have thoroughly reviewed the reference list and corrected all formatting inconsistencies.

Comment 10: Language and Writing Quality

The manuscript is generally well-written and understandable. However, there are several grammatical errors, typographical issues, and inconsistencies in terminology. Some sentences are overly long or repetitive. Professional language editing is recommended to improve clarity and readability.

Response 10: We thank the reviewer for this note. The entire manuscript has undergone comprehensive proofreading and professional language editing to eliminate grammatical errors, fix typographical issues, and break down overly long or repetitive sentences.
