# Supplementary material for: The combined role of education and wealth in menstrual health: Evidence from Bangladesh
Source: PLoS One. 2026 Oct 5;21(10):e0346457. doi: 10.1371/journal.pone.0346457 (PMC13637891; doi:10.1371/journal.pone.0346457)
Supplement: Supplementary file 11 [file pone.0346457.s007.pdf]

**The Combined Role of Education and Wealth in Menstrual Hygiene: Evidence from Bangladesh**

## Rebuttal letter

**Reviewer 03:**

**Comment 1:** In my initial review, I suggested that there was a need for a much stronger discussion on why education is a proxy for awareness – as well as a stronger explanation of what awareness refers to. While this has been significantly strengthened in the second iteration of the paper, I am still not left feeling sold on this approach. Both proxy indicators – educational attainment and household income – lack nuance in how they are cross-walked to awareness and affordability, respectively. My suggestion would be to either: re-focus the paper on educational attainment and household income, and the impacts of these variables on sanitary napkin use OR further develop the proxy analysis.

I suggest doing the first option. There is not a compelling reason to me that the paper needs to focus on awareness rather than educational attainment, or on household income rather than affordability. I think that re-focusing the paper would make a much clearer, much more compelling analysis on the factors that influence sanitary napkin use. You could then, in the discussion/conclusion sections, layer in how these variables contribute to overall awareness or affordability. You would be in a position of having fewer limitations/restrictions to the analysis that you offer.

If you go with the second option, I would suggest: (1) acknowledging that several variables you discuss contribute to awareness, for example media exposure in addition to educational attainment. It would be helpful to pull each of these together and use them as different proxies for awareness, but rolled up into a more cohesive analysis; (2) acknowledging that educational attainment is flawed as a proxy variable. Much of your paper focuses on how educational curriculum doesn't include menstrual health or how young adolescents are unaware of menstrual hygiene at the age of menarche. This is coupled with the reality that the most vulnerable populations are often the ones not in school. To use this proxy, I suggest being upfront that you recognize these contradictions and limitations, and posing why the analysis still holds. Then, I would suggest strengthening the affordability analysis. In order for household income to be a proxy for affordability, you need some analysis on how product prices are affordable within the context of these different income amounts. How does household income relate to disposable income, for example? And what is the relationship to product prices. Again, I think going with option #1 will feel much more doable and succinct in the context of this analysis – and then you can bring these types of issues into the limitations and discussion.

**Response 01:** Thank you for this thoughtful and constructive suggestion. We agree that educational attainment and household wealth are more appropriately interpreted as socioeconomic determinants rather than direct proxies for awareness and affordability. Accordingly, we have revised the manuscript

to adopt **your first suggestion** by refocusing the analysis on the combined roles of educational attainment and household wealth in sanitary napkin use. References to awareness and affordability as proxy measures have been removed from the main analysis and are now discussed only as potential mechanisms underlying the observed associations.

**Comment 02:** I would still love to see a stronger discussion of sanitary napkin use. The authors did a great job of better defining 'sanitary napkins' in this revision, but still don't include any discussion on why the analysis is specific to sanitary napkins and not a broader suite of menstrual products (e.g. inclusion of cups, reusable sanitary napkins, etc). The focus on sanitary napkins is fine, but should include a justification for why other products aren't included. Is this a limitation of the data available? Is it that sanitary napkins are a high enough percent of the products used in Bangladesh that there is not a reason to ask about other products? More discussion here would be valuable.

**Response 02:** Thank you for this suggestion. We have addressed this point in the Introduction (**Lines 83–92**) by explaining that, although other modern menstrual products are available, their use remains limited in Bangladesh. As sanitary napkins are the most widely accepted and readily available modern menstrual product, we justify their use as the study outcome.

**Comment 03:** Finally, I still think that a title modification might be valuable. Since your key takeaway is essentially that both awareness and affordability matter for menstrual health, positing them as potentially conflicting doesn't resonate as much to me. Given my comments under point #2, I also think that focusing more on educational attainment/household income makes the most sense in the title.

**Response 03:** Thank you for the helpful suggestion. We have revised the title to better reflect the study's main focus to "**The Combined Role of Education and Wealth in Menstrual Hygiene: Evidence from Bangladesh**".
