# Supplementary material for: The combined role of education and wealth in menstrual health: Evidence from Bangladesh
Source: PLoS One. 2026 Oct 5;21(10):e0346457. doi: 10.1371/journal.pone.0346457 (PMC13637891; doi:10.1371/journal.pone.0346457)
Supplement: Supplementary file 12 [file pone.0346457.s009.pdf]

# <u>Rebuttal letter</u>

**Comment 1:** Please go through the document and change “menstrual hygiene” to “menstrual health.”

**Response 1:** We thank the reviewer for this suggestion. The term "menstrual health" has been updated throughout the manuscript accordingly.

**Comment 3:** Figure 1 would be better if it also had markings (eg diagonal lines in a printed fashion) in areas of wealth, so that we can see at a glance if the high usage areas corresponded with top tier wealth.

**Response 3:** We thank the reviewer for this helpful suggestion. Figure 1 has been updated to include diagonal hatching across high-wealth districts (>60% upper wealth quintiles) to allow for immediate visual comparison with usage levels. The figure caption and Methods section have been updated accordingly.

**Comment 5:** P11 line 114. It would be useful to include a power analysis to ascertain that 20 households per group would represent potential significance.

**Response 5:** We thank the reviewer for this comment. We have now added the power analysis in **lines 118-122** of the Methods section.
