# Supplementary material for: The combined role of education and wealth in menstrual health: Evidence from Bangladesh
Source: PLoS One. 2026 Oct 5;21(10):e0346457. doi: 10.1371/journal.pone.0346457 (PMC13637891; doi:10.1371/journal.pone.0346457)
Supplement: Supplementary file 8 [file pone.0346457.s001.docx]

**The Combined Role of Education and Wealth in Menstrual Health: Evidence from Bangladesh**

Mohammad Hridoy Patwary^1^, Md Tazvir Amin^2*^, Sumaiya Nusrat Esha^1^, Md Mahabubur Rahman^2^

Maternal and Child Health Division, icddr,b., GPO Box 128, Dhaka 1000, Bangladesh^1^

Health Systems and Population Studies Division, icddr,b, GPO Box 128, Dhaka 1000, Bangladesh^2^

Corresponding author^*^

E-mail: tazvir.amin@icddrb.org; tamin@isrt.ac.bd

**Supplementary Table 1.** Distribution of sanitary napkin usage by sociodemographic variables among women aged 15-49 years in Bangladesh, 2019.

|  | **n** | **%** | **P-value** |
| --- | --- | --- | --- |
| **Total** | 10,922 | 23.4 |  |
| **Age group** |  |  |  |
| 45-49 | 307 | 9.6 |  |
| 40-44 | 631 | 11.9 |  |
| 35-39 | 1,180 | 15 |  |
| 30-34 | 1,592 | 18.3 | <0.001 |
| 25-29 | 2,248 | 26.6 |  |
| 20-24 | 3,107 | 35.7 |  |
| 15-19 | 1,857 | 41.6 |  |
| **Educational attainment** |  |  |  |
| Pre-primary or none | 195 | 2.7 |  |
| Primary | 741 | 6.7 | <0.001 |
| Secondary or above | 9,987 | 35.1 |  |
| **Media exposure** |  |  |  |
| Not at all | 1,226 | 8.9 |  |
| Less than once a week | 343 | 15.4 | <0.001 |
| At least once a week | 685 | 17.5 |  |
| Almost every day | 8,668 | 32.4 |  |
| **Disability** |  |  |  |
| Not disable | 9,255 | 25.3 | <0.001 |
| Disable | 1,667 | 16.5 |  |
| **Wealth index** |  |  |  |
| Bottom two | 987 | 6 | <0.001 |
| Upper three | 9,935 | 33 |  |
| **Education & WI** |  |  |  |
| Pre-primary or none & Bottom two | 45 | 1.06 |  |
| Pre-primary or none & Upper three | 149 | 5.09 |  |
| Primary & Bottom two | 136 | 2.50 | <0.001 |
| Primary & Upper three | 605 | 10.81 |  |
| Secondary or above & Bottom two | 806 | 11.84 |  |
| Secondary or above & Upper three | 9180 | 42.46 |  |
| **Educational attainment of HH** |  |  |  |
| Pre-primary or none | 1,572 | 10.8 | <0.001 |
| Primary | 1,992 | 15.7 |  |
| Secondary or above | 7,358 | 37.8 |  |
| **Religious affiliation** |  |  |  |
| Muslim | 9,871 | 23.4 | 0.642 |
| Other Religion | 1,051 | 23.1 |  |
| **Sex of HH** |  |  |  |
| Female | 1,523 | 27.7 |  |
| Male | 9,399 | 22.8 |  |
| **Area of residence** |  |  | <0.001 |
| Rural | 6,176 | 17.5 |  |
| Urban | 4,746 | 41.5 |  |
| **Division** |  |  |  |
| Dhaka | 3,997 | 33 |  |
| Barishal | 508 | 20.5 |  |
| Chattogram | 2,107 | 23.8 |  |
| Khulna | 1,363 | 24.7 | <0.001 |
| Mymensingh | 393 | 12.8 |  |
| Rajshahi | 1,125 | 18 |  |
| Rangpur | 747 | 15 |  |
| Sylhet | 683 | 19.9 |  |
