# Supplementary material for: The combined role of education and wealth in menstrual health: Evidence from Bangladesh
Source: PLoS One. 2026 Oct 5;21(10):e0346457. doi: 10.1371/journal.pone.0346457 (PMC13637891; doi:10.1371/journal.pone.0346457)
Supplement: Supplementary file 10003 [file pone.0346457.s003.pdf]

PLOS One

Awareness or Affordability: What matters the most in ensuring menstrual hygiene management?

--Manuscript Draft--

**Manuscript Number:**	PONE-D-26-13387
**Article Type:**	Research Article
**Full Title:**	Awareness or Affordability: What matters the most in ensuring menstrual hygiene management?
**Short Title:**	Awareness vs Affordability in Menstrual Hygiene
**Corresponding Author:**	Mohammad Hridoy Patwary<br>ICDDRB: International Centre for Diarrhoeal Disease Research Bangladesh<br>Dhaka, BANGLADESH
**Keywords:**	Menstrual hygiene management<br>Sanitary napkin use<br>MICS<br>Causal Inference<br>Interaction effect
**Abstract:**	Background<br><br>Ensuring menstrual hygiene management (MHM) remains a critical public health challenge, particularly in resource-limited settings. While affordability is often cited as a primary barrier to sanitary napkin use, awareness regarding menstrual hygiene may also play a crucial role. This study examines the interplay between awareness and affordability in determining the use of sanitary napkins in Bangladesh.<br><br>Methods<br><br>We used nationally representative data from the 2019 Bangladesh Multiple Indicator Cluster Survey (MICS). Type of material used by women during their last menstruation was used as the outcome variable where individual educational attainment and household wealth index were used as proxies for awareness and affordability, respectively. A generalized linear model (GLM) with a logit link function was used to estimate adjusted odds ratios (AORs). Then using the fitted model, marginal probabilities of sanitary napkin use were calculated for subgroups defined by combinations of wealth index and educational attainment.<br><br>Findings<br><br>The likelihood of sanitary napkin use was significantly associated with both awareness and affordability. Women with secondary or higher education had 4.5 times more odds (AOR=4.50, 95% CI: 3.28–6.16) of using sanitary napkins compared to those with no formal education. Similarly, women from the upper three wealth quintiles had 2.3 times higher odds (AOR=2.30, 95% CI: 1.58–3.34) of using sanitary napkins compared to those in the lowest two quintiles. Women with secondary or higher education from lower wealth quintiles were more likely to use sanitary napkins than their counterparts with pre-primary or no education from wealthier households (marginal probability: 15.46% vs 9.25%).<br><br>Conclusion<br><br>Both awareness and affordability play a critical role in determining menstrual hygiene practices among Bangladeshi women. While financial constraints remain a key barrier, the findings suggest that improving awareness could significantly enhance MHM outcomes, even among lower-income groups. Policies should prioritize integrated interventions that address both economic and informational barriers to ensure equitable access to sanitary products.
**Order of Authors:**	Mohammad Hridoy Patwary

	Md Tazvir Amin
	Sumaiya Nusrat
	Md Mahabubur Rahman
**Additional Information:**	
**Question**	**Response**
**Financial Disclosure**<br><br>Enter a financial disclosure statement that describes the sources of funding for the work included in this submission. Review the submission guidelines for detailed requirements. View published research articles from *PLOS ONE* for specific examples.<br><br>This statement is required for submission and **will appear in the published article** if the submission is accepted. Please make sure it is accurate.<br><br>**Funded studies**<br>Enter a statement with the following details:<br>• Initials of the authors who received each award<br>• Grant numbers awarded to each author<br>• The full name of each funder<br>• URL of each funder website<br>• Did the sponsors or funders play any role in the study design, data collection and analysis, decision to publish, or preparation of the manuscript?<br><br>Did you receive funding for this work?	The author(s) received no specific funding for this work.
**Competing Interests**<br><br>Use the instructions below to enter a competing interest statement for this submission. On behalf of all authors, disclose any competing interests that could be perceived to bias this work—acknowledging all financial support and any other relevant financial or non-financial competing interests.<br><br>This statement is required for submission	None declared

and **will appear in the published article** if the submission is accepted. Please make sure it is accurate and that any funding sources listed in your Funding Information later in the submission form are also declared in your Financial Disclosure statement.<br><br>View published research articles from *PLOS ONE* for specific examples.<br><br>**NO authors have competing interests**<br><br>Enter: *The authors have declared that no competing interests exist.*<br><br>**Authors with competing interests**<br><br>Enter competing interest details beginning with this statement:<br><br>*I have read the journal's policy and the authors of this manuscript have the following competing interests: [insert competing interests here]*<br><br>* typeset	
**Ethics Statement**<br><br>Enter an ethics statement for this submission. This statement is required if the study involved:<br><br>• Human participants<br>• Human specimens or tissue<br>• Vertebrate animals or cephalopods<br>• Vertebrate embryos or tissues<br>• Field research<br><br>Write "N/A" if the submission does not require an ethics statement.<br><br>General guidance is provided below. Consult the submission guidelines for detailed instructions. **Make sure that all information entered here is included in the Methods section of the manuscript.**	Not applicable. As the study utilized publicly available and anonymized secondary data, it does not require additional ethical approval.

**Format for specific study types**<br><br>**Human Subject Research (involving human participants and/or tissue)**<br>• Give the name of the institutional review board or ethics committee that approved the study<br>• Include the approval number and/or a statement indicating approval of this research<br>• Indicate the form of consent obtained (written/oral) or the reason that consent was not obtained (e.g. the data were analyzed anonymously)<br><br>**Animal Research (involving vertebrate animals, embryos or tissues)**<br>• Provide the name of the Institutional Animal Care and Use Committee (IACUC) or other relevant ethics board that reviewed the study protocol, and indicate whether they approved this research or granted a formal waiver of ethical approval<br>• Include an approval number if one was obtained<br>• If the study involved *non-human primates*, add *additional details* about animal welfare and steps taken to ameliorate suffering<br>• If anesthesia, euthanasia, or any kind of animal sacrifice is part of the study, include briefly which substances and/or methods were applied<br><br>**Field Research**<br><br>Include the following details if this study involves the collection of plant, animal, or other materials from a natural setting:<br>• Field permit number<br>• Name of the institution or relevant body that granted permission	
**Data Availability**<br><br>Authors are required to make all data underlying the findings described fully available, without restriction, and from the time of publication. PLOS allows rare exceptions to address legal and ethical concerns. See the PLOS Data Policy and FAQ for detailed information.	Yes - all data are fully available without restriction

A Data Availability Statement describing where the data can be found is required at submission. Your answers to this question constitute the Data Availability Statement and **will be published in the article**, if accepted.<br><br>**Important:** Stating 'data available on request from the author' is not sufficient. If your data are only available upon request, select 'No' for the first question and explain your exceptional situation in the text box.<br><br>Do the authors confirm that all data underlying the findings described in their manuscript are fully available without restriction?	
**Describe where the data may be found in full sentences. If you are copying our sample text, replace any instances of XXX with the appropriate details.**<br><br>• If the data are **held or will be held in a public repository**, include URLs, accession numbers or DOIs. If this information will only be available after acceptance, indicate this by ticking the box below. For example: *All XXX files are available from the XXX database (accession number(s) XXX, XXX.).*<br>• If the data are all contained **within the manuscript and/or Supporting Information files**, enter the following: *All relevant data are within the manuscript and its Supporting Information files.*<br>• If neither of these applies but you are able to provide **details of access elsewhere**, with or without limitations, please do so. For example:<br><br>*Data cannot be shared publicly because of [XXX]. Data are available from the XXX Institutional Data Access / Ethics Committee (contact via XXX) for researchers who meet the criteria for access to confidential data.*<br><br>*The data underlying the results presented in the study are available from (include the name of the third party*	The datasets analyzed during the current study are available on the MICS program website, https://microdata.worldbank.org/index.php/catalog/MICS/

and contact information or URL).<br>• This text is appropriate if the data are owned by a third party and authors do not have permission to share the data.<br><br>* typeset	
Additional data availability information:	

Powered by Editorial Manager® and ProduXion Manager® from Aries Systems Corporation

Manuscript
Click here to access/download;Manuscript;MHM_Manuscript_17March2026.d

Awareness or Affordability: What matters the most in ensuring menstrual hygiene management?

Mohammad Hridoy Patwary[1]; Md Tazvir Amin[2*]; Sumaiya Nusrat Esha[1]; Md Mahabubur Rahman[2]

Maternal and Child Health Division, icddr,b., GPO Box 128, Dhaka 1000, Bangladesh[1]

Health Systems and Population Studies Division, icddr,b, GPO Box 128, Dhaka 1000, Bangladesh[2]

Corresponding author*

E-mail: tazvir.amin@icddrb.org; tamin@isrt.ac.bd

Abstract

Background

Ensuring menstrual hygiene management (MHM) remains a critical public health challenge, particularly in resource-limited settings. While affordability is often cited as a primary barrier to sanitary napkin use, awareness regarding menstrual hygiene may also play a crucial role. This study examines the interplay between awareness and affordability in determining the use of sanitary napkins in Bangladesh.

Methods

We used nationally representative data from the 2019 Bangladesh Multiple Indicator Cluster Survey (MICS). Type of material used by women during their last menstruation was used as the outcome variable where individual educational attainment and household wealth index were used as proxies for awareness and affordability, respectively. A generalized linear model (GLM) with a logit link function was used to estimate adjusted odds ratios (AORs). Then using the fitted model, marginal probabilities of sanitary napkin use were calculated for subgroups defined by combinations of wealth index and educational attainment.

Findings

The likelihood of sanitary napkin use was significantly associated with both awareness and affordability. Women with secondary or higher education had 4.5 times more odds (AOR=4.50, 95% CI: 3.28–6.16) of using sanitary napkins compared to those with no formal education. Similarly, women from the upper three wealth quintiles had 2.3 times higher odds (AOR=2.30, 95% CI: 1.58–3.34) of using sanitary napkins compared to those in the lowest two quintiles. Women with secondary or higher education from lower wealth quintiles were more likely to use sanitary napkins than their counterparts with pre-primary or no education from wealthier households (marginal probability: 15.46% vs 9.25%).

Conclusion

Both awareness and affordability play a critical role in determining menstrual hygiene practices among Bangladeshi women. While financial constraints remain a key barrier, the findings suggest that improving awareness could significantly enhance MHM outcomes, even among lower-income groups. Policies should prioritize integrated interventions that address both economic and informational barriers to ensure equitable access to sanitary products.

Introduction

Menstruation is a natural physiological process that generally indicates the onset of reproductive lifespan for a girl, characterized by the regular discharge of blood and tissue from the uterus through the vagina every month [1]. The first occurrence of menstruation (aka Menarche) usually happens between the ages of 12 to 15 and represents a trans-formative phase in the lives of adolescent girls, bringing along new challenges and vulnerabilities [2, 3]. Despite being a natural and essential bodily process, menstruation has been surrounded by taboos, myths, and secrecy in many cultures for a long time, leading to stigma, shame, and silence around the world, especially in low- and middle-income countries (LMICs) [4-6]. This stigma and shame can have significant negative impacts on the emotional well-being and self-esteem of menstruating individuals, particularly young girls as they transition into adolescence [7, 8].

Each month, approximately 1.8 billion girls worldwide experience menstruation, yet millions lack the resources to manage their menstrual hygiene in a dignified and healthy way due to limited affordability, lack of awareness, stigma, taboos, and silence about menstrual hygiene management (MHM) [9, 10]. This poor management of menstrual hygiene is associated with many adverse health outcomes such as lower RTIs, secondary infertility, and abnormal vaginal discharge [11-14]. It also affects the quality of life and performance in schools and workplaces, a significant barrier to women's empowerment and social inclusion [15-17].

Menstrual hygiene management (MHM) has become a global public health concern, emphasizing individuals' practices and actions to maintain cleanliness, dignity, and health during menstruation [18]. MHM is also closely linked with health, social justice, and human rights [18]. MHM has also become a vital focus within the Sustainable Development Goals (SDGs) due to its importance in enhancing women's physical and mental well-being and promoting social inclusion [19]. It involves using proper menstrual hygiene products (i.e., napkins, tampons, dry clothes, menstrual

cups, etc), sanitation facilities (i.e. washing the clothes, drying them in sunlight, disposal of the menstrual product properly, etc.), and healthy behaviors (changing the napkins or clothes regularly at least 4 times a day) during menstruation [19].

Recognizing the impact of inadequate MHM on health, education, and gender equality, various efforts have been undertaken in LMICs to address these challenges. Governments, international organizations, and local NGOs have implemented programs to improve access to menstrual products, sanitation facilities, and menstrual health education. Yet, the pervasive stigma and silence surrounding menstruation in LMICs like Bangladesh obstruct access to rights-based information and services regarding MHM [20]. Around half of the adolescent girls in rural Bangladesh are not very confident about managing their menstrual bleeding, even in their own house, where this percentage in school is less than 10% [20]. Poor menstrual hygiene management also affects their performance in school and overall life [21, 22]. National representative data shows that 30% of school girls experienced an average monthly absence of 2.5 days due to menstruation [23]. Though the average age of menarche in Bangladesh is higher (12.8) than the global average (12.4), only a quarter of adolescent girls know about menstruation before their menarche [23-25]. Only 24.3% of women and adolescent girls in Bangladesh use proper modern absorbents, and fewer than 20% of old cloth users adhere to the recommended cleaning and drying practices [23, 26].

While traditional practices such as using old cloths are deeply rooted in cultural norms and have been passed down across generations, they often lack hygienic standards and are associated with a higher risk of infection and discomfort [27]. Introducing sanitary napkins challenges these long-standing customs and requires not only behavioral change but also sustained access to affordable and acceptable alternatives. This dual requirement of behavioral shift and financial investment highlights the importance of both awareness and affordability in adopting improved menstrual hygiene practices.

While a growing body of research has acknowledged these two factors as critical determinants of sanitary napkin use, the literature remains fragmented. Previous studies have typically examined the effects of either awareness or affordability in isolation, suggesting that higher education and better economic status are individually associated with improved MHM [26, 28]. However, there is a notable gap in the literature regarding their combined and potentially interactive influence. Specifically, little is known about how awareness and affordability intersect to influence behavior

in diverse socioeconomic contexts across Bangladesh. Addressing this gap is crucial for designing effective interventions. Without understanding how these two dimensions reinforce or offset each other, policy efforts risk being incomplete or misdirected. Moreover, most existing studies provide limited insights into regional disparities, despite the fact that menstrual health behaviors and barriers can vary widely by geography, culture, and local infrastructure. Therefore, this study aims to examine how women's awareness and affordability jointly shape the use of sanitary napkins, offering evidence that can inform both national and region-specific strategies to enhance menstrual hygiene management in Bangladesh.

Methods

Data overview

This study utilized data from the 2019 Bangladesh Multiple Indicator Cluster Survey (MICS), conducted by the Bangladesh Bureau of Statistics (BBS) with technical support from UNICEF. The survey employed a two-stage stratified sampling design to collect nationally representative information on the health and well-being of women and children across all districts of Bangladesh, covering both urban and rural areas.

A total of 3,220 primary sampling units (PSUs) and 64,400 households were included in the survey. In the first stage, enumeration areas (EAs) were selected using probability proportional to size. In the second stage, 20 households were randomly selected from each selected EA.

The survey collected detailed information from women of reproductive age (15–49 years). Sampling weights provided in the dataset were applied in all analyses to account for the complex survey design and ensure national representativeness. The dataset used in this study is publicly available (accessed in January, 2023) and de-identified, with no information that could be used to identify individual participants.

Outcome variable

The outcome variable for this study was whether the participant used a sanitary napkin during her last menstruation. The original variable, which included various materials (e.g., cloth, tissue, cotton pads), was recoded into a binary variable: sanitary napkin vs. others. In this study, sanitary napkin use was considered a proxy for proper menstrual hygiene, as they are typically cleaner, safer, and more absorbent than traditional or improvised materials.

Explanatory variables

As individual-level indicators of health awareness and affordability were not available in the dataset, proxies were used to represent these constructs. Educational attainment was employed as a proxy for awareness, as higher education is generally associated with better knowledge of health

practices [29]. Similarly, the household wealth index was used as a proxy for affordability, as it reflects the economic capacity of a household to purchase menstrual hygiene products and related resources. Other variables considered in this study were identified from relevant literature from Bangladesh [26], India, Pakistan, and Nepal (Table 1) [30-32]. Individual level factors that can affect use of sanitary napkin included participants age, educational attainment, exposure to media, and status of disability. Household level factors consists of wealth quintile, religious belief of household along with sex and educational attainment of household head. Community level prevalence of sanitary napkin usage, type of residence and division of the participants were included as community level factor.

Table 1. Description of variables used in the study

Explanatory variable	Categories	Description
**Individual-level factors**		
Age	15-19	Respondent's current age is between 15 and 19 years.
	20-24	Respondent's current age is between 20 and 24 years.
	25-29	Respondent's current age is between 25 and 29 years.
	30-34	Respondent's current age is between 30 and 34 years.
	35-39	Respondent's current age is between 35 and 39 years.
	40-45	Respondent's current age is between 40 and 44 years.
	45+	Respondent's current age is above 45 years.
Educational attainment	Pre-primary or none	No formal education
	Primary	1–5 years of formal schooling
	Secondary or above	6–10 or more than 10 years of formal schooling
Media Exposure	No media exposure	Respondent does not get any exposure to mass media.
	At least once a week	Get exposure at least once a week from at least one media.
	Almost everyday	Get exposure almost every day from at least one media.
Disability	Yes	Respondent has at least one disability among difficulty seeing, hearing, walking, remembering or concentrating, self-care, and communicating.
	No	Respondent has no such disabilities.
**Household-level factors**		
Wealth Index	Bottom two	Household belongs to the bottom two wealth quantile.
	Upper three	Household belongs to the upper three wealth quantile.

Educational attainment of household head	Pre-primary or none	No formal education
	Primary	1–5 years of formal schooling
	Secondary or above	6–10 or more than 10 years of formal schooling
Sex of household head	Male	Sex of the household head is male.
	Female	Sex of the household head is female.
Religious affiliation	Muslim	Respondent belongs to a Muslim household.
	Non-Muslim	Respondent belongs to a non-Muslim household.
**Community-level factors**		
Community prevalence of sanitary napkin usage	Low prevalence	The community prevalence of sanitary napkins use is below 30%.
	Moderate prevalence	The community prevalence of sanitary napkins use is between 30 to 60%.
	High prevalence	The community prevalence of sanitary napkins use is higher than 60%.
Area of Residence	Rural	Respondent's type of place of residence is rural.
	Urban	Respondent's type of place of residence is urban.
Division	Dhaka	Respondent lives in the Dhaka division
	Barisal	Respondent lives in the Barisal division
	Chattogram	Respondent lives in the Chattogram division
	Khulna	Respondent lives in the Khulna division
	Mymensingh	Respondent lives in the Mymensingh division.
	Rajhshahi	Respondent lives in the Rajshahi division
	Rangpur	Respondent lives in the Rangpur division.
	Sylhet	Respondent lives in the Sylhet division.

Statistical analyses

Univariate analysis was conducted to examine the sample characteristics. The chi-square (χ^2) test was used to assess associations between the outcome variable and explanatory variables. The outcome of interest, use of sanitary napkins, was then modeled using a generalized linear model (GLM) with a logit link function to estimate adjusted odds ratios (AORs) for the study factors. After fitting the model, the marginal probabilities for six groups, defined by combinations of wealth index and individual education level, were estimated using the margins command in Stata (Stata SE V.14, StataCorp). Additionally, the spatial distribution of sanitary napkin usage across districts was illustrated using the R packages *sf* and *ggplot2* (V 4.3.2). All analyses accounted for survey weights that were provided in the dataset, to ensure representativeness.

Results

Table 2 presents the demographic characteristics of 46,677 surveyed women aged 15-49 years. Notably, a predominant age group falls within the range of 20-39 years, representing 18.62% of the total sample. Educational attainment reveals a substantial majority, with 60.91% of respondents

possessing secondary or higher qualifications. Regarding media exposure, a significant portion, accounting for 57.33% of the sample, reported being exposed to media almost daily. Approximately one-fifth (21.58%) of respondents acknowledged having a disability. Socioeconomic status demonstrates a notable disparity, with 35.41% of individuals falling within the bottom two wealth quantiles and 64.59% within the upper three. An examination of educational attainment within wealth indices reveals intriguing variations. Religious demographics indicate a Muslim majority, comprising 90.24% of the sample, while male-headed households constitute 88.23%. Notably, 72.81% of the surveyed women reported

Table 2. Sample characteristics of the study participants.

	Distribution	
	**n (unweighted)**	**% (weighted)**
**Total**	46,677	100
**Age group**		
15-19	4,462	9.56
20-24	8,690	18.62
25-29	8,453	18.11
30-34	8,712	18.66
35-39	7,854	16.83
40-44	5,300	11.35
45-49	3,205	6.87
**Educational attainment**		
Pre-primary or none	7,209	15.44
Primary	11,039	23.65
Secondary or above	28,429	60.91
**Media exposure**		
Not at all	13,767	29.49
Less than once a week	2,233	4.78
At least once a week	3,914	8.39
Almost every day	26,762	57.33
**Disability**		
Not disable	36,593	78.4
Disable	10,075	21.58
**Wealth Index**		
Bottom two	16,530	35.41
Upper three	30,147	64.59
**Educational attainment**		
Pre-primary or none	14,503	31.07
Primary	12,728	27.27
Secondary or above	19,445	41.66
**WI & Educational attainment**		

	Distribution	
	**n (unweighted)**	**% (weighted)**
Pre-primary or none & Bottom two	4,278	9.17
Pre-primary or none & Upper Three	2,931	6.28
Primary & Bottom two	5,444	11.66
Primary & Upper Three	5,595	11.99
Secondary & Bottom two	6,808	14.59
Secondary & Upper three	21,621	46.32
**Religious affiliation**		
Other Religion	4,556	9.76
Muslim	42,121	90.24
**Sex of household head**		
Female	5,492	11.77
Male	41,184	88.23
**Community prevalence of sanitary napkin usage**		
Low prevalence	33,821	72.81
Moderate prevalence	10,188	21.93
High prevalence	2,444	5.26
**Area of residence**		
Rural	35,245	75.51
Urban	11,432	24.49
**Division**		
Barisal	2,482	5.32
Chattogram	8,835	18.93
Dhaka	12,114	25.95
Khulna	5,526	11.84
Mymensingh	3,062	6.56
Rajshahi	6,262	13.42
Rangpur	4,966	10.64
Sylhet	3,429	7.35

using sanitary napkins, with the majority residing in rural areas (75.51%). Geographically, Dhaka emerges as the most represented division (25.95%), followed by Chattogram (18.93%).

The distribution of the proportion of women using sanitary napkins across districts in Bangladesh is illustrated in Figure 1. The spatial distribution reveals a generally poor level of sanitary napkin use throughout the country. In only five out of 64 districts does nearly one in three women use sanitary napkins—still a very low proportion. The northern region shows the lowest usage rates, with Lalmonirhat having the lowest proportion, where approximately 6% of women use sanitary napkin.

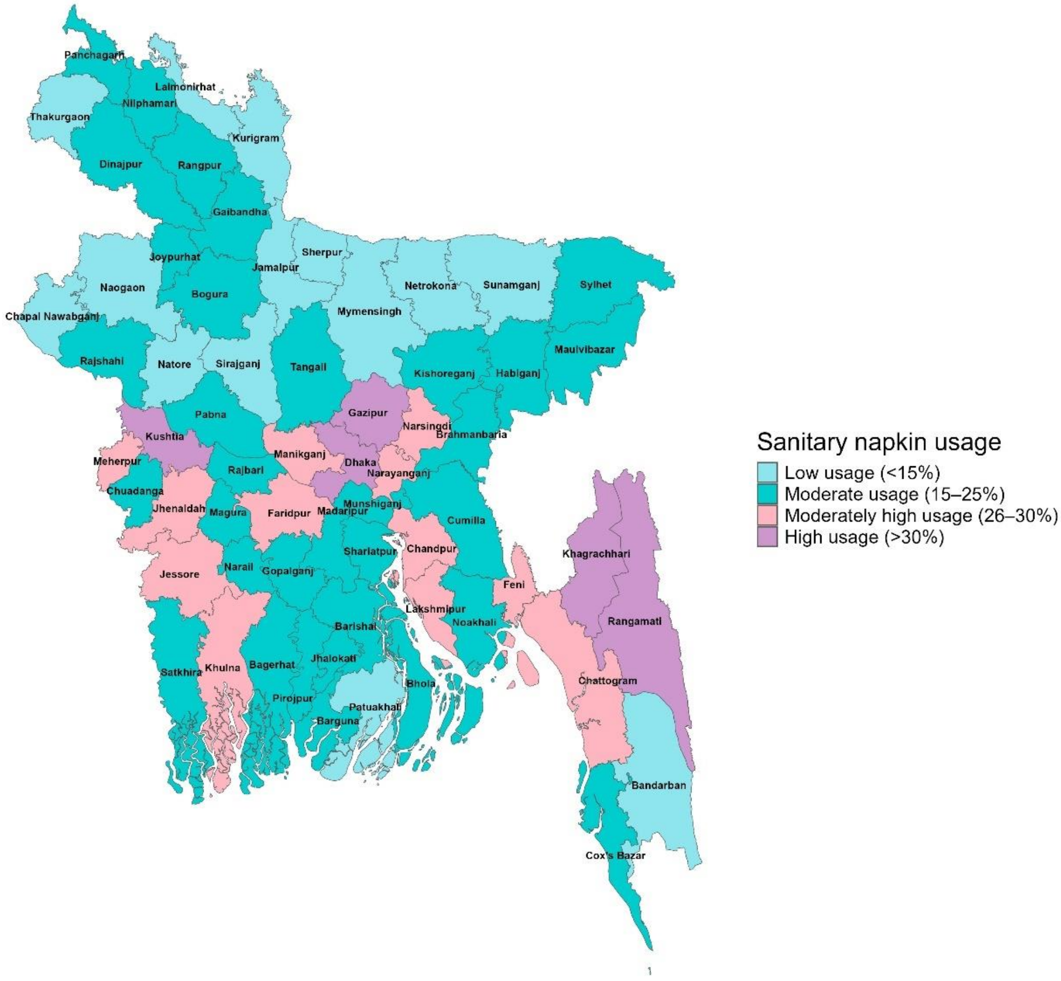

Fig 1. District-wise mapping on the distribution of sanitary napkin usage in Bangladesh

Sanitary napkin usage by education and wealth

The overall prevalence of sanitary napkin usage among women aged 15–49 years was 23.4% (Supplementary table 1). Women with secondary or higher education from the bottom two wealth quintiles had a higher prevalence of sanitary napkin use (11.84%) than women from the upper three wealth quintiles who had pre-primary or no education (5.09%) or only primary education (10.82%).

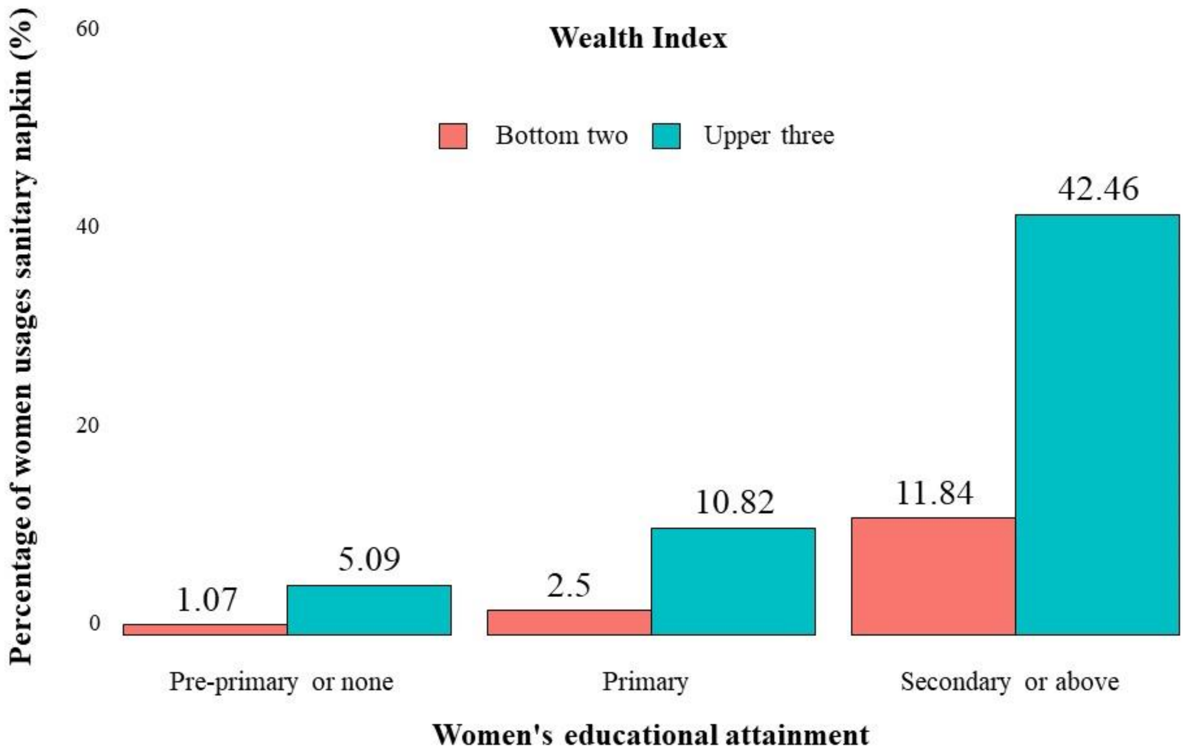

Fig 2. Percentage of women usage sanitary napkins over educational attainment and wealth index

Table 3 presents adjusted odds ratios (AOR) with associated p-values and 95% confidence intervals (CI) for the relationship between the type of material used during menstruation and various demographic factors. Notable findings include a higher likelihood of using sanitary napkins in younger age groups, with substantial increases in odds ratios from 25-29 years (AOR=2.57) to 15-19 years (AOR=6.09). Education plays a significant role, with increasing AORs for primary (AOR=1.44) and Secondary or above (AOR=4.5) compared to pre-primary or none. Media exposure shows a positive association, with higher odds for more frequent exposure. Individuals without disabilities have higher odds (AOR=0.86) of using Sanitary Napkins. The wealth index indicates significantly higher odds (AOR=2.3) in the upper three quantiles. Notable variations exist in the odds of sanitary napkin usage across different divisions and areas of residence, with urban residents, Dhaka division, and areas with high community prevalence having higher odds.

Table 3. Association of women's education and wealth with sanitary napkin usage

	AOR	p-value	95% CI	
			Lower	Upper
**Educational attainment**				
Pre-primary or none	1 (Ref.)			
Primary	1.44	0.048	1.0	2.05
Secondary or above	4.50	<0.01	3.28	6.16
**Wealth index**				
Bottom two	1 (Ref.)			
Upper three	2.30	<0.01	1.58	3.34
**Education & WI**				
Primary & Upper three	1.03	0.90	0.66	1.59
Secondary or above & Upper three	1.33	0.144	0.91	1.95
**Age**				
45-49	1 (Ref.)			
40-44	1.08	0.389	0.90	1.29
35-39	1.34	<0.01	1.14	1.58
30-34	1.54	<0.01	1.30	1.83
25-29	2.57	<0.01	2.18	3.03
20-24	4.17	<0.01	3.54	4.92
15-19	6.09	<0.01	5.13	7.23
**Media exposure**				
Not at all	1 (Ref.)			
Less than once a week	1.27	<0.01	1.08	1.51
At least once a week	1.42	<0.01	1.25	1.62
Almost every day	1.93	<0.01	1.76	2.11
**Disability**				
Not disable	1 (Ref.)			
Disable	0.86	<0.01	0.80	0.93
**Educational attainment of HH**				
pre-primary or none	1 (Ref.)			
primary	1.12	0.03	1.01	1.23
secondary or above	2.11	<0.01	1.92	2.30
**Religious affiliation**				
Muslim	1 (Ref.)			
Other Religion	1.04	0.454	0.93	1.17
**Sex of Household head**				
Female	1 (Ref.)			
Male	0.84	<0.01	0.76	0.93
**Community prevalence of sanitary napkin usage**				
Low Use	1 (Ref.)			
Moderate Use	1.80	<0.01	1.59	2.03
High Use	3.75	<0.01	3.06	4.59
**Area of residence**				

	AOR	p-value	95% CI	
			Lower	Upper
Rural	1 (Ref.)			
Urban	1.50	<0.01	1.38	1.64
**Division**				
Dhaka	1 (Ref.)			
Barishal	1.15	0.02	1.02	1.29
Chattogram	0.74	<0.01	0.68	0.80
Khulna	0.92	0.06	0.84	1.01
Mymensingh	0.60	<0.01	0.50	0.71
Rajshahi	0.68	<0.01	0.62	0.76
Rangpur	0.67	<0.01	0.60	0.75
Sylhet	0.85	0.051	0.73	1.00
**Intercept**	0.01	<0.01	0.00	0.01

From the fitted regression model, we have calculated the marginal probabilities of sanitary napkin usage for every subgroup created by two variables: educational attainment and wealth quintiles. The national estimates from figure 3 shows that, educational attainment significantly increases the likelihood of using sanitary napking among women aged 15-49 years in Bangladesh. Women with secondary or higher educational attainment from a poor household (bottom two) had higher chance of use sanitary napkin compared to women with pre-primary or no educational attainment from a rich household (upper three). This behavibiour was consistent across all divisions.

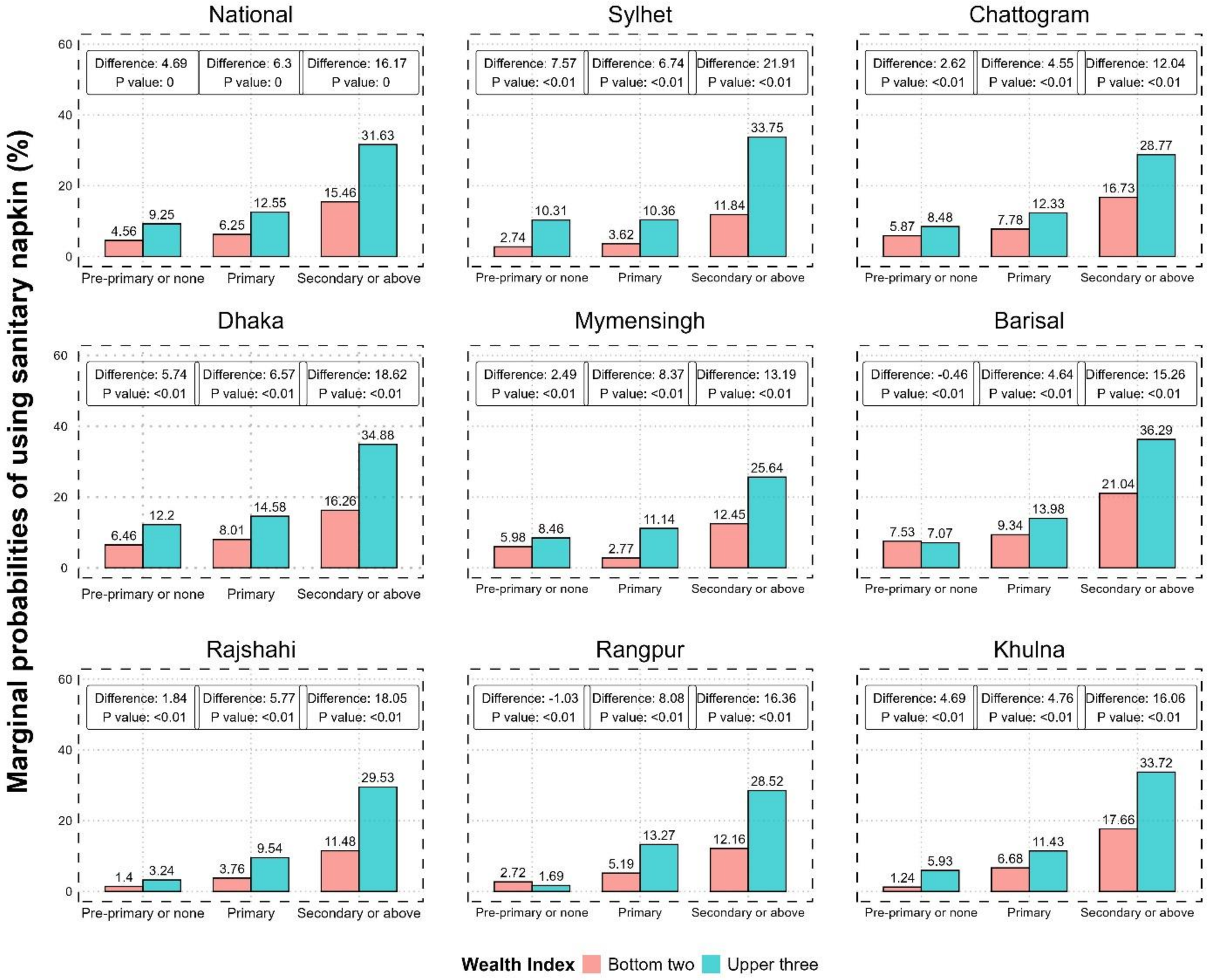

p-value from t-test

Fig 3. Marginal probabilities of sanitary napkin usage by women's education and wealth.

Discussion

This study highlights the significant roles of both awareness and affordability in determining sanitary napkin use among women in Bangladesh. The results suggest that awareness has a particularly strong association with sanitary napkin use compared to affordability. Besides less educated, younger, and disabled women, those living in male-headed or less educated households, with limited media exposure, residing in rural areas or in divisions like Mymensingh and Rangpur, and living in communities with low sanitary napkin use were less likely to use sanitary napkins.

Comparison with earlier studies

The study observed significant differences in menstrual hygiene practices across divisions, urban and rural areas which are in line with previous studies [17]. This difference is might be due to some unobserved factors, which vary across regions. Another areal-level factor influencing sanitary napkin uses is the community-level prevalence of sanitary napkin use, which reflects shared norms, awareness, and accessibility. In Bangladesh, where cultural stigmas around menstruation remain strong, women and girls in communities with higher sanitary napkin use are more likely to adopt proper hygiene practices. This is because such communities often foster greater awareness and openness. In contrast, lower-prevalence areas tend to be more restrictive and less informed. Similar patterns have been observed elsewhere: in India, Sood et al. found that positive social norms significantly improved MHM [33]; in Nepal, Shrestha et al. reported that caste-based taboos limited awareness regarding MHM [34].

Apart from area-level factors, household-level factors also significantly influence the menstrual practices of women and adolescent girls. Women in male-headed households are less likely to have adequate knowledge of menstrual hygiene management compared to those in female-headed or households with an educated head [26]. which, in turn, negatively affect menstrual hygiene practices.

Lastly, individual-level factors have a significant role in explaining the variation of sanitary napkin uses. Mass media exposure seen to have a significant role in menstrual hygiene practices which serves as a core source of information on MHM [30]. Women who are exposed to media more frequently are more likely to have better knowledge and use of sanitary products, with those accessing media almost every day showing the highest likelihood. The intersection of menstruation and disability endangers an intricate set of circumstances that can give rise to compounded discrimination and present distinct challenges for menstruating individuals with disabilities [24]. This study also found evidence supporting this phenomenon.

Interplay between awareness and affordability in napkin use

Our findings reveal that both awareness and affordability significantly influence the use of sanitary napkins, underscoring their interdependent roles in ensuring effective MHM. While higher levels of awareness are strongly associated with increased use, this effect is amplified in the presence of affordability, indicating a synergistic relationship between the two factors.

Awareness enhances knowledge of menstruation as a biological process, reduces stigma, and improves attitudes towards the use of hygienic products [35]. Due to lack of awareness, menstrual hygiene is often deprioritized and menstrual products are overlooked in basic WASH and sanitation planning [36]. Regardless of affordability, many girls still refrain from using them due to embarrassment and stigma. A cross-sectional survey of adolescent girls in Rajshahi revealed that, factors like embarrassment purchasing pads from male shopkeepers and stigma surrounding menstruation were major barriers to sanitary napkin use [37].

However, knowledge alone does not always translate into practice when economic barriers remain. This is particularly relevant in low-income households where affordability and availability is the key barriers [38]. In such settings, even when girls are aware of the benefits of sanitary napkins, they may be forced to rely on unsafe alternatives due to cost constraints.

Action taken to promote sanitary napkins

In Bangladesh, the government recognizes MHM as essential for women's and girls' health and has prioritized awareness and affordability in the National MHM Strategy 2021 [39]. To reduce costs and promote local manufacturing, the government has waived VAT on raw materials for sanitary napkin production. Additionally, the National MHM Coordination Committee has been established to oversee and align MHM efforts across health, education, and social sectors.

Despite these efforts, a 15% VAT on finished sanitary napkin products remains a significant barrier to affordability. To address this, the strategy calls for revisiting the VAT policy to make napkins more affordable. It also emphasizes improving distribution networks to ensure access in workplaces, schools, healthcare centers, and public spaces, alongside expanding MHM awareness sessions to marginalized and hard-to-reach communities.

Notably, the current NCTB curriculum does not adequately address menstruation and menstrual hygiene management, particularly in the subject "Physical Education and Health" for classes 6, 7, and 8[40-42]. These textbooks lack essential information on menstruation, the use of sanitary napkins, and guidance on changing intervals, which are critical for maintaining good menstrual hygiene practices. As a result, many adolescent girls remain uninformed or misinformed about safe and hygienic practices during menstruation. Revising the curriculum to include these crucial topics is imperative for equipping adolescents with the accurate knowledge and practical skills they need to manage menstruation.

On the other hand, non-governmental research organizations have implemented programs to increase awareness about menstruation. One such initiative was the Ritu Programme, funded by the Embassy of the Kingdom of the Netherlands in Bangladesh and implemented from November 2015 to March 2020. The programme aimed to improve menstrual health among girls aged 10–15 in Netrokona, Bangladesh[43]. Through evidence-informed interventions—including school-based menstrual health education, community engagement with parents, and a national-level mass media campaign—the programme significantly boosted menstruation awareness and management. It notably increased girls' confidence and understanding of menstruation, improved hygiene practices (like changing materials more frequently and increased sanitary pad use).

Taken together, the findings underscore that MHM is shaped by a complex interplay of structural, social, and individual determinants. While individual awareness and household affordability are critical, they do not operate in isolation; rather, they are embedded within broader contextual factors such as community norms, media exposure, educational content, and policy frameworks. Therefore, effective menstrual health interventions must adopt a multi-level approach that simultaneously targets these interconnected domains.

Strengths and Limitations of the study

A key strength of this study is the use of nationally representative MICS 2019 data, which ensures broad generalizability. It applies a generalized linear model to estimate adjusted associations and calculates marginal probabilities to clearly quantify the combined effects of education and wealth

on sanitary napkin use. By directly comparing subgroups (e.g., educated-poor vs. uneducated-rich), the study provides concrete evidence on the relative influence of awareness and affordability, offering actionable insights for targeted MHM interventions.

This study has several limitations. First, the cross-sectional nature of the data limits the scope of estimating the causal effects. Second, awareness and affordability were measured using proxy indicators—educational attainment and household wealth index—which may not fully capture the complexity of these constructs. Third, self-reported data on menstrual hygiene practices may be subject to social desirability or recall bias. Lastly, certain contextual factors such as cultural norms, school-based education on menstruation, and access to WASH (Water, Sanitation, and Hygiene) facilities were not available in the dataset and could not be accounted for in the analysis.

Conclusion

This study demonstrates that menstrual hygiene management is influenced by interconnected factors at the individual, household, and community levels. Awareness and affordability show significant interaction effects, with awareness emerging as a key driver of sanitary napkin use across wealth quintiles. However, awareness alone cannot overcome the barriers posed by limited economic resources.

To ensure equitable and effective menstrual hygiene management, a dual approach is essential—one that combines widespread menstrual health education with access to affordable sanitary products. Only by addressing both informational and economic barriers can we promote safe, dignified, and sustainable menstrual practices for all women and girls in Bangladesh.

Declarations

Ethics approval and consent to participate

Not applicable. As the study utilized publicly available and anonymized secondary data, it does not require additional ethical approval.

Consent for publication

Not applicable.

Availability of data and materials

The datasets analyzed during the current study are available on the MICS program website, https://microdata.worldbank.org/index.php/catalog/MICS/

Competing interests

None declared.

Funding

The authors received no specific grant funding for this research.

Authors' contributions

MHP, MTA, and MMR conceptualized the study. MHP and MMR performed data curation and conducted the formal statistical analysis. MHP, MTA, SNE, and MMR contributed to the writing of the original draft. SNE contributed to the literature review. All authors reviewed, edited, and provided critical feedback on the manuscript. All authors read and approved the final version of the manuscript.

Acknowledgments

The authors would like to thank UNICEF and the Bangladesh Bureau of Statistics (BBS) for providing access to the 2019 Bangladesh Multiple Indicator Cluster Survey (MICS) dataset. icddr,b acknowledges the Government of the People's Republic of Bangladesh and Canada for providing core/unrestricted support.

References

1. Habeeb, S., *Menstruation*, in *Encyclopedia of Sexual Psychology and Behavior*, T.K. Shackelford, Editor. 2023, Springer International Publishing: Cham. p. 1-11.
2. Critchley, H.O.D., et al., *Physiology of the Endometrium and Regulation of Menstruation.* Physiol Rev, 2020. **100**(3): p. 1149-1179.
3. Likis, K.D.S.a.F.E., *Women's Gynecologic Health*. Vol. Second Edition. 2013.
4. Dutta, D., et al., *Supporting the rights of girls and women through Menstrual Hygiene Management (MHM) in the East Asia and Pacific Region: Realities, progress and opportunities.* Bangkok, Thailand: UNICEF East Asia and Pacific Regional Office (EAPRO), 2016.
5. Sommer, M. and M. Sahin, *Overcoming the taboo: advancing the global agenda for menstrual hygiene management for schoolgirls.* American journal of public health, 2013. **103**(9): p. 1556-1559.
6. Korir, E., F.N. Okwara, and G. Okumbe, *Menstrual hygiene management practices among primary school girls from a pastoralist community in Kenya: a cross sectional survey.* Pan African Medical Journal, 2018. **31**(1).
7. Olson, M.M., et al., *The persistent power of stigma: A critical review of policy initiatives to break the menstrual silence and advance menstrual literacy.* PLOS Global Public Health, 2022. **2**(7): p. e0000070.
8. Maulingin-Gumbaketi, E., et al., *Socio-cultural implications for women's menstrual health in the Pacific Island Countries and Territories (PICTs): a scoping review.* Reproductive health, 2022. **19**(1): p. 128.
9. UNICEF, *Menstrual hygiene | UNICEF. Avilable: https://www.unicef.org/wash/menstrual-hygiene.* 2023.
10. UNICEF. *Menstrual hygiene: Gender inequality, cultural taboos and poverty can cause menstrual health needs to go unmet.* . Available from: https://www.unicef.org/wash/menstrual-hygiene.
11. Ali, T.S., N. Sami, and A.K. Khuwaja, *Are unhygienic practices during the menstrual, partum and postpartum periods risk factors for secondary infertility?* J Health Popul Nutr, 2007. **25**(2): p. 189-94.
12. Anand, E., J. Singh, and S. Unisa, *Menstrual hygiene practices and its association with reproductive tract infections and abnormal vaginal discharge among women in India.* Sex Reprod Healthc, 2015. **6**(4): p. 249-54.
13. Das, P., et al., *Menstrual Hygiene Practices, WASH Access and the Risk of Urogenital Infection in Women from Odisha, India.* PLoS One, 2015. **10**(6): p. e0130777.
14. Torondel, B., et al., *Association between unhygienic menstrual management practices and prevalence of lower reproductive tract infections: a hospital-based cross-sectional study in Odisha, India.* BMC Infect Dis, 2018. **18**(1): p. 473.
15. Hennegan, J. and P. Montgomery, *Do Menstrual Hygiene Management Interventions Improve Education and Psychosocial Outcomes for Women and Girls in Low and Middle Income Countries? A Systematic Review.* PLoS One, 2016. **11**(2): p. e0146985.
16. Krenz, A. and H. Strulik, *The impact of menstruation hygiene management on work absenteeism of women in Burkina Faso.* Econ Hum Biol, 2021. **43**: p. 101067.

17. Tegegne, T.K. and M.M. Sisay, *Menstrual hygiene management and school absenteeism among female adolescent students in Northeast Ethiopia.* BMC Public Health, 2014. **14**: p. 1118.
18. Sommer, M., et al., *A Time for Global Action: Addressing Girls' Menstrual Hygiene Management Needs in Schools.* PLoS Med, 2016. **13**(2): p. e1001962.
19. Sommer, M., et al., *How addressing menstrual health and hygiene may enable progress across the Sustainable Development Goals.* Glob Health Action, 2021. **14**(1): p. 1920315.
20. Hennegan, J. and L. Sol, *Confidence to manage menstruation at home and at school: findings from a cross-sectional survey of schoolgirls in rural Bangladesh.* Culture, health & sexuality, 2020. **22**(2): p. 146-165.
21. Ahmed, M.S., et al., *Association between menstrual hygiene management and school performance among the school-going girls in rural Bangladesh.* Adolescents, 2021. **1**(3): p. 335-347.
22. Hasan, M., et al., *Menstrual hygiene practices and school absenteeism among adolescent girls in Bangladesh: A cross-sectional study. Popul Med 2021; 3: 8*. 2021.
23. Khan, S., et al., *Taking measures before opportunities bleed away: Current knowledge and policy gaps in menstrual hygiene management among adolescent girls in Bangladesh.* United States Agency for International Development (USAID), 2022.
24. A. Lacroix, H.G., K. Shumway, and M. Langaker. *Physiology, Starting*. Available from: https://gifsided.com/female-reproductive-system-questions-and-answers-pdf.
25. Sibli, M.S., et al., *Analysis of the average menopause age of Bangladeshi women: A public health perspective.* World Journal of Advanced Research and Reviews, 2022. **16**(3): p. 231-235.
26. Afiaz, A. and R.K. Biswas, *Awareness on menstrual hygiene management in Bangladesh and the possibilities of media interventions: using a nationwide cross-sectional survey.* BMJ Open, 2021. **11**(4): p. e042134.
27. Sumpter, C. and B. Torondel, *A systematic review of the health and social effects of menstrual hygiene management.* PLoS One, 2013. **8**(4): p. e62004.
28. Mondal, B.K., et al., *Practices and effects of menstrual hygiene management in rural Bangladesh.* 2017.
29. Jejeebhoy, S.J., *Women's education, autonomy, and reproductive behaviour: Experience from developing countries*. 1995: Oxford University Press.
30. Garg, S., et al., *Menstrual hygiene management and its determinants among adolescent girls in low-income urban areas of Delhi, India: a community-based study.* Osong Public Health Res Perspect, 2022. **13**(4): p. 273-281.
31. Wasan, Y., et al., *Practices and predictors of menstrual hygiene management material use among adolescent and young women in rural Pakistan: A cross-sectional assessment.* J Glob Health, 2022. **12**: p. 04059.
32. Bhusal, C.K., *Practice of Menstrual Hygiene and Associated Factors among Adolescent School Girls in Dang District, Nepal.* Adv Prev Med, 2020. **2020**: p. 1292070.
33. Sood, S., et al., *Measuring the effectiveness of communication programming on menstrual health and hygiene management (MHM) social norms among adolescent girls in India.* Glob Public Health, 2021. **16**(4): p. 578-589.
34. Khanal, G., et al., *Menstruation hygiene management among secondary school students of Chitwan, Nepal:a cross-sectional study.* BMC Women's Health, 2023. **23**(1): p. 395.

35. Sommer, M., et al., *Comfortably, Safely, and Without Shame: Defining Menstrual Hygiene Management as a Public Health Issue.* Am J Public Health, 2015. **105**(7): p. 1302-11.
36. Mahon, T. and M. Fernandes, *Menstrual hygiene in South Asia: a neglected issue for WASH (water, sanitation and hygiene) programmes.* Gender & Development, 2010. **18**(1): p. 99-113.
37. Ha, M.A.T. and M.Z. Alam, *Menstrual hygiene management practice among adolescent girls: an urban-rural comparative study in Rajshahi division, Bangladesh.* BMC Womens Health, 2022. **22**(1): p. 86.
38. Dasgupta, A. and M. Sarkar, *Menstrual Hygiene: How Hygienic is the Adolescent Girl?* Indian J Community Med, 2008. **33**(2): p. 77-80.
39. *Government of Bangladesh. (2021). National Menstrual Hygiene Management (MHM) Strategy 2021. Ministry of Women and Children Affairs. Available at: https://psb.gov.bd/policies/nmhmsen.pdf.*
40. *National Curriculum and Textbook Board (NCTB). (2024). Physical Education & Health, Class 6. Dhaka: National Curriculum and Textbook Board.*
41. *National Curriculum and Textbook Board (NCTB). (2024). Physical Education & Health, Class 7. Dhaka: National Curriculum and Textbook Board.*
42. *National Curriculum and Textbook Board (NCTB). (2024). Physical Education & Health, Class 8. Dhaka: National Curriculum and Textbook Board.*
43. *Simavi, RedOrange, TNO, BNPS, & DORP. (2020). Ritu programme – Improving menstrual health of girls in Bangladesh: Final report 2016-2020.*

Click here to access/download
Supporting Information
MHM_Supplementary.pdf
