# Supplementary material for: The combined role of education and wealth in menstrual health: Evidence from Bangladesh
Source: PLoS One. 2026 Oct 5;21(10):e0346457. doi: 10.1371/journal.pone.0346457 (PMC13637891; doi:10.1371/journal.pone.0346457)
Supplement: Supplementary file 10004 [file pone.0346457.s005.pdf]

PLOS One

Awareness or Affordability: What matters the most in ensuring menstrual hygiene?

--Manuscript Draft--

**Manuscript Number:**	PONE-D-26-13387R1
**Article Type:**	Research Article
**Full Title:**	Awareness or Affordability: What matters the most in ensuring menstrual hygiene?
**Short Title:**	Awareness vs Affordability in Menstrual Hygiene
**Corresponding Author:**	Md Tazvir Amin<br>ICDDRB: International Centre for Diarrhoeal Disease Research Bangladesh<br>Rangamati, BANGLADESH
**Keywords:**	Menstrual hygiene management<br>Sanitary napkin use<br>MICS<br>Causal Inference<br>Interaction effect
**Abstract:**	Background<br>Ensuring menstrual hygiene remains a critical public health challenge, particularly in resource-limited settings. Both affordability and awareness are considered to play a crucial role in ensuring menstrual hygiene. However, the interactive effect of awareness and affordability remain unclear, particularly in the context of Bangladesh. To address this gap, this study examines the interplay between awareness and affordability in determining the use of sanitary napkins in Bangladesh.<br>Methods<br>We used nationally representative data from the 2019 Bangladesh Multiple Indicator Cluster Survey (MICS). Type of material used by women during their last menstruation was used as the outcome variable. Based on literature, individual educational attainment and household wealth index were used as sociodemographic proxies for awareness regarding menstrual hygiene management and affordability to buy sanitary napkins, respectively. A generalized linear model (GLM) with a logit link function was used to estimate adjusted odds ratios (AORs). Then using the fitted model, marginal probabilities of sanitary napkin use were estimated for subgroups defined by combinations of wealth index and educational attainment.<br>Findings<br>The national prevalence of sanitary napkin use was 23.4%. Odds of sanitary napkin use was 4.5 times higher (AOR=4.50, 95% CI: 3.28–6.16) among women with secondary or higher education compared to those with no formal education. Similarly, women from the upper three wealth quintiles had 2.3 times higher odds (AOR=2.30, 95% CI: 1.58–3.34) of using sanitary napkins compared to those in the lowest two quintiles. Women with secondary or higher education from lower wealth quintiles were more likely to use sanitary napkins than with pre-primary or no education from wealthier households (marginal probability: 15.46% vs 9.25%).<br>Conclusion<br>Both awareness and affordability play a critical role in determining menstrual hygiene practices among Bangladeshi women. While financial constraints remain a key barrier, the findings suggest that improving awareness could significantly enhance hygienic menstrual practices, even among lower-socioeconomic groups. Policies should prioritize interventions for improving awareness of menstrual hygiene management.
**Order of Authors:**	Mohammad Hridoy Patwary<br>Md Tazvir Amin<br>Sumaiya Nusrat<br>Md Mahabubur Rahman
**Response to Reviewers:**	Awareness or Affordability: What matters the most in ensuring menstrual hygiene management?<br>Rebuttal Letter<br>Reviewer 03.

Powered by Editorial Manager® and ProduXion Manager® from Aries Systems Corporation

Comment 1 (line 17: awareness regarding menstrual hygiene): This will require a more detailed definition in the paper -- awareness of menstrual hygiene from a health/biological standpoint? Management of menstrual hygiene/product use? Options for products? What do we mean by awareness here?
Response 1: We have now clear out what do we meant by awareness here (knowledge regarding the menstrual hygiene management) and also broaden the explanation in the explanatory variables part (lines 131).
Comment 2 (line 23: individual educational attainment): I'd be curious to see more justifications around the selection of this proxy -- e.g. some sort of evidence/justification around school/educational facilities being the critical entry point to awareness around menstrual products.
Response 2: We thank the reviewer for this important comment. We have justified the use of this proxy in the explanatory variable part (lines 131-134).
Comment 3 (line 31: sanitary napkins): Were only sanitary napkins assessed? Or was this meant to be inclusive of the full range of menstrual products?
Response 3: The term “sanitary napkins” refers to reported use of sanitary pads during the last menstrual period, rather than assessment of a full range of individual product types separately (please see the updated outcome variable section; lines 122-125).
Comment 4 (line 58: This poor management of menstrual hygiene): Suggest re-framing to a way that doesn't place blame on the person who menstruates -- there has largely been a shift away from framing around 'menstrual hygiene' and towards 'menstrual health' for this reason. In this framing, it sounds like the person who menstruates is to blame for these health outcomes, rather than the system that has failed them.
Response 4: We thank the reviewer for this important suggestion. We have revised the text accordingly (lines 56-57).
Comment 5 (line 81-82: only a quarter of adolescent girls know about menstruation before their menarche): This seems to suggest that menstrual health is not taught in schools, which feels counter to the proxy indicator of educational status to indicate awareness about menstrual health/menstrual products.
Response 5: We thank the reviewer for this sharp observation. We agree that the low rate of pre-menarche knowledge seems contradictory at first glance. This statistic reflects a well-documented reality in Bangladesh: reproductive health topics are often introduced too late in the curriculum (or skipped altogether in primary school), leaving young girls uninformed at the actual onset of menarche. However, our proxy of "educational attainment" for women aged 15–49 measures their cumulative exposure to schooling. As women progress into secondary and higher education, they gain delayed but crucial exposure to health curricula. Thus, while early school fails to prepare them before menarche, longer retention in the education system is still the primary driver of MHM awareness.

Comment 6 (line 117: The survey collected detailed information from women of reproductive age (15–49 years)): Suggest acknowledging the gap on young adolescent data -- the average age of menarche is much younger than 15, which means that we aren't capturing the full menstruating population with these data. This is hard to address since MICS / survey data don't include this, but suggest highlighting this potential gap.
Response 6: We have added this as a limitation (lines 325-327).
Comment 7 (line 125-127: In this study, sanitary napkin use was considered a proxy for proper menstrual hygiene, as they are typically cleaner, safer, and more absorbent than traditional or improvised materials): Better define what is meant by sanitary napkin and whether this includes both reusable and disposable options or just disposable/single use. Include context on whether cloth/non-purpose made materials were considered to 'count' as sanitary napkins in this definition.
Response 7: We thank the reviewer for this comment. Sanitary napkin use is defined as any reported use of purpose-made sanitary pads during the last menstrual period, regardless of exclusive or combined use (refer to the updated outcome variable section).
Comment 8 (line 128): An additional variable that should be part of this analysis is actual availability of products -- availability itself is often a barrier and if sanitary napkins are not readily available in certain communities (most often, rural or vulnerable communities, areas with lower household wealth, etc), this creates a confounding variable that influences outcome. Suggest adding analysis on this that speaks to how readily available products are in these regions... beyond just actual use of products.

Powered by Editorial Manager® and ProduXion Manager® from Aries Systems Corporation

Use of products and prevalence of community use still rely on products being available to purchase and use.
Response 8: We thank the reviewer for this point. Unfortunately, the 2019 Bangladesh MICS dataset does not contain variables on commercial market supply or physical product availability in communities.
Comment 9 (line 135-137: Individual level factors that can affect use of sanitary napkin included participants age, educational attainment, exposure to media, and status of disability): Suggest acknowledging that you are missing a critical age group within these data, since the data begin at age 15 but average age of menarche is much lower. I would acknowledge this and contextualize a bit further.
Response 9: We have added this as a limitation (lines 325-327).
Comment 10 (Table 1: The community prevalence of sanitary napkins use is below 30%): Contextualize what this includes. Can you also clarify how prevalence is defined here? Is it a simple yes/no on access to sanitary pads, or does it measure for *sufficient* access, e.g. sufficient product access for the full cycle, rather than supplementation with non-purpose made products (e.g. 1-4 sanitary pads per cycle, supplemented with cloth or other materials).
Response 10: We thank the reviewer for this comment. The community prevalence was built on to the binary outcome defined in the outcome variable section, where sanitary napkin use was measured as any reported use during the last menstrual period (yes/no), regardless of quantity or whether other materials were also used (lines 144-146).
Comment 11 (lines 192-194: Notable findings include a higher likelihood of using sanitary napkins in younger age groups, with substantial increases in odds ratios from 25-29 years (AOR=2.57) to 15-19 years (AOR=6.09)): This seems counter to the findings that higher educational levels result in higher likelihood of use, as most women/girls with higher education are likely to be older than those with lower.
Response 11: We thank the reviewer for this important comment. The findings reflect two different effects estimated in the adjusted model. Age captures cohort differences in menstrual hygiene practices, where younger women are more likely to adopt sanitary napkins due to more recent exposure to menstrual health information and changing norms. Education, on the other hand, reflects individual awareness and knowledge, which increases the likelihood of sanitary napkin use across all age groups. Therefore, higher use among younger women and more educated women are not contradictory, but represent separate pathways influencing behavior.
Comment 12 (lines 206-207: educational attainment significantly increases the likelihood of using sanitary napking among women aged 15-49 years in Bangladesh): I think that this is a more powerful finding than framing the findings around educational attainment as being a proxy for 'awareness.'
Response 12: We agree with the reviewer that formal educational attainment is a powerful determinant with broader societal implications beyond acting as a proxy for awareness. However, to keep the paper tightly aligned with our research question, our analysis and discussion focus specifically on its role in health awareness and information access.
Comment 13 (lines 216-217: This study highlights the significant roles of both awareness and affordability in determining sanitary napkin use among women in Bangladesh): For this takeaway to be highlighted, I think that much more work is needed to do justify educational attainment as a proxy for awareness. I think that the stronger framing is that educational attainment is significant in determining sanitary napkin use -- and a potential policy takeaway/discussion lens could be unpacking how education might lead to increased awareness. I also think that we need much more contextual definition on what is meant by awareness here.
Response 13: We thank the reviewer for this observation. As detailed in our Explanatory Variables (lines 131-138) and Discussion sections (lines 256-259), we use formal education as an established proxy for health awareness because higher educational attainment is a primary structural pathway through which women access health literacy and information on menstrual hygiene.
Comment 14 (lines 225: Comparison with earlier studies): Is this the right header here? This seems to be looking at other individual factors within the context of this study, rather than prior studies. Additional studies are woven in to this discussion, but suggest re-framing the header to focus more on the other individual factors as the focus of this section.
Response 14: We thank the reviewer for this helpful suggestion. We have revised the header to “Factors influencing sanitary napkin use”.

Comment 15 (lines 232: proper hygiene practices.): Avoid framing like 'proper' hygiene practices -- this shifts blame to the person menstruating rather than a system that has failed her.
Response 15: We thank the reviewer for this insightful comment. We agree that the term "proper" can inadvertently imply individual blame. We have revised the manuscript to replace "proper" with "recommended" to focus on the practices themselves rather than casting judgment on the individual.
Comment 16 (lines 255-258): I think that this context needs to come much earlier in the paper to help readers understand what is meant by awareness -- and why education might be a proxy for it.
Response 16: We thank the reviewer for the suggestion. In the revised manuscript, we have broadened the rationale for using these proxies (lines 131-138).

Comment 17 (lines 258-261): This next part seems to focus more on stigma/shame/taboo than on awareness. I would suggest either (1) expanding on why/how awareness reduces stigma/shame and therefore overcomes this; or (2) saying that even with increased awareness, stigma/shame pose further barriers to access.
Response 17: We thank the reviewer for this helpful suggestion. We have revised the Discussion (according to option 2) to clarify that (lines 262-264).
Comment 18 (lines 263: availability): Did your statistical analysis take this into account at all? E.g. was there any analysis done on whether availability itself might have been why people weren't using sanitary napkins? These products tend to be less available in rural communities, lower-income areas, or less-educated communities -- could availability itself have been a confounding factor that wasn't explored?
Response 18: We thank the reviewer for this point. Unfortunately, the 2019 Bangladesh MICS dataset does not contain variables on commercial market supply or physical product availability in communities.
Comment 19 (lines 279-281: These textbooks lack essential information on menstruation, the use of sanitary napkins, and guidance on changing intervals, which are critical for maintaining good menstrual hygiene practices.): This adds to my comment throughout questioning the proxy of education to awareness -- this seems to imply that education doesn't equate to awareness. Suggest addressing more directly this tension.
Response 19: We thank the reviewer for highlighting this apparent tension. There are two key points to this:
First, there is a chronological distinction. Our study utilizes 2019 MICS data, representing a period when the national curriculum did contain basic components of menstrual health. The omission of these critical components occurred during the recent 2025 national curriculum update. We have now clear out this in line 281.
Second, utilizing educational attainment as a proxy for MHM awareness does not strictly assume that MHM components must be present in the textbooks. Formal schooling builds general health literacy, provides the cognitive tools to process external health information, and fosters crucial peer-to-peer knowledge sharing among adolescent girls.
Comment 20 (lines 296-297: rather, they are embedded within broader contextual factors such as community norms, media exposure, educational content, and policy frameworks.): And also market dynamics -- how available are products?
Response 20: We thank the reviewer for this important point. We acknowledge that market dynamics, including product availability and supply-side factors, are also relevant contextual determinants of menstrual hygiene management. However, as our dataset did not include information on product availability, we were unable to assess this aspect in the present study.
Comment 21 (lines 304: effects of education and wealth): Suggest framing like this more consistently throughout -- rather than around 'awareness.'
Response 21: We thank the reviewer for pointing this out. We have now revised the terms to make them consistent.
Comment 22 (lines 309-311: Second, awareness and affordability were measured using proxy indicators—educational attainment and household wealth index—which may not fully capture the complexity of these constructs.): Suggest expansion on this one.
Response 22: We agree. We have expanded this limitation section to clarify that individual educational attainment does not directly capture specific menstrual health literacy, and the household wealth index does not necessarily reflect an individual

woman's financial autonomy or direct purchasing power. Please refer to lines 315-321 in the revised manuscript.

Reviewer 01:

Comment 01: Overall Evaluation
This manuscript addresses a highly relevant public health issue, menstrual hygiene management (MHM) by examining the relative and combined influence of awareness and affordability on sanitary napkin use in Bangladesh. The use of nationally representative MICS data and application of regression and marginal probability analyses are notable strengths. The study provides valuable insights for policy and program design in low-resource settings. However, despite its strengths, the manuscript requires major revision due to conceptual oversimplification, methodological limitations (particularly proxy measurement), insufficient critical interpretation, and language inconsistencies. Strengthening these areas will significantly enhance the rigor and contribution of the study.
Response 01: We sincerely thank the reviewer for their encouraging assessment of our study's strengths and its relevance to public health. We have carefully considered this feedback and have addressed the concerns accordingly. Please find our detailed, point-by-point responses to each specific comment below.
Comment 02: Title and Abstract
The title is clear, engaging, and directly reflects the core research question, effectively highlighting the comparison between awareness and affordability. The abstract is well-structured and summarizes the background, methods, findings, and conclusions. However, it remains largely descriptive and lacks emphasis on novelty and theoretical contribution. The use of education and wealth index as proxies for awareness and affordability, respectively, is mentioned but not critically justified in the abstract. Additionally, the conclusion may slightly overstate the causal implication, given the cross-sectional design. The abstract would benefit from a clearer statement of the research gap and a more cautious interpretation of findings.
Response 02: We thank the reviewer for this constructive feedback. We have revised the Abstract to explicitly state the research gap (the lack of studies exploring the interactive effects of awareness and affordability) and provided a brief justification for using education and wealth index as operational proxies.
Comment 03: Introduction
The introduction provides a comprehensive overview of menstruation, MHM, and its global and national significance. It effectively contextualizes the issue within Bangladesh using relevant statistics and literature. The discussion of stigma, health outcomes, and social implications is appropriate and well-supported. Importantly, the authors identify a research gap regarding the interaction between awareness and affordability, which is a strong aspect of the manuscript.
However, the section could be improved by tightening its structure and reducing redundancy. Some paragraphs are overly descriptive and could be more concise. Additionally, while the research gap is mentioned, it could be articulated more sharply to emphasize the novelty of the interaction analysis. The conceptual distinction between awareness (education) and affordability (wealth index) should also be more critically justified, as these proxies may not fully capture the constructs.
Response 03: We thank the reviewer for the positive evaluation. We have significantly tightened this section and sharpened the description of the research gap (lines 95-102). For the proxy, we have broaden the justification of using these indicators in the method section (lines 131-138).
Comment 04: Methods
The methods section is generally well-organized and provides sufficient detail about the data source, sampling design, and analytical approach. The use of nationally representative MICS data strengthens the external validity of the findings. The application of generalized linear models and marginal probability estimation is appropriate and enhances interpretability.
However, there are several important limitations. First, the study relies on proxy variables, education for awareness and wealth index for affordability which may not accurately reflect the intended constructs. This introduces potential measurement bias and should be more critically discussed. Second, the cross-sectional design limits causal inference, yet the manuscript occasionally implies causality. Third, although survey weights are applied, there is limited discussion on handling potential confounding or multicollinearity. Finally, the binary classification of sanitary napkin use

as a proxy for “proper MHM” may oversimplify menstrual practices, as other safe alternatives (e.g., menstrual cups) are not considered.
Response 04: We thank the reviewer for confirming the appropriateness of our analytical approach. We completely agree with the identified limitations. As noted, because this study is a secondary analysis of survey data, these constraints are structural. We have fully acknowledged and critically discussed each of these points in the Strengths and Limitations section of the discussion.
Comment 05: Results
The results are clearly presented, with appropriate use of tables, figures, and statistical estimates. The descriptive statistics provide a comprehensive overview of the sample characteristics. The regression analysis and adjusted odds ratios are well reported, and the inclusion of marginal probabilities is a strong addition that improves interpretability. The spatial analysis (district-level mapping) adds value by highlighting geographic disparities. Figures (e.g., Fig 1 and Fig 2) effectively illustrate differences in sanitary napkin use across regions and socioeconomic groups. The finding that education (awareness) may outweigh wealth (affordability) in certain contexts is particularly interesting.
However, the results section is somewhat descriptive and could benefit from clearer emphasis on key findings. Additionally, some interpretations are presented within the results rather than reserved for the discussion. Greater clarity in linking statistical findings to the research question would improve this section.
Response 05: We thank the reviewer for the highly positive feedback on our presentation of the results. To address the concern regarding descriptive tone and interpretation, we have carefully re-reviewed the Results section to ensure that statistical findings are tied directly to our core research questions and that any explanatory interpretation is strictly reserved for the discussion section.
Comment 06: Discussion
The discussion appropriately interprets the findings and situates them within existing literature. The emphasis on the interaction between awareness and affordability is a key strength, and the discussion of community, household, and individual-level factors provides a multi-level perspective.
However, the discussion remains somewhat descriptive and lacks deeper critical analysis. The manuscript would benefit from stronger theoretical framing, for example by linking findings to behavior change theories or health access models. While policy implications are mentioned, they are not sufficiently developed or specific. The claim that awareness has a stronger effect than affordability should be interpreted more cautiously, considering the limitations of proxy variables.
The section on policy and program interventions (e.g., VAT policy, curriculum gaps, and Ritu program) is informative but could be more directly linked to the study findings. Additionally, there is some repetition across paragraphs, and the overall structure could be more concise.
Response 06: We have revised the Discussion to provide a more critical analysis. We integrated two theoretical frameworks, the Andersen Model of Health Services Use and Social Cognitive Theory, to better explain the multi-level factors shaping MHM behaviors. Per your suggestion, we now interpret the comparison between awareness and affordability more cautiously, acknowledging the limitations of our proxy variables. Finally, we directly linked policy interventions (VAT and curriculum gaps) to our empirical findings and streamlined the section to improve conciseness.
Comment 07: Strengths and Limitations
The authors acknowledge several strengths, including the use of nationally representative data and advanced statistical methods. The discussion of limitations is appropriate, particularly regarding the cross-sectional design and use of proxy variables.
However, this section could be expanded further. For instance, the potential for residual confounding, measurement error, and reporting bias should be more explicitly discussed. The limitation regarding the binary outcome variable and exclusion of other menstrual products should also be highlighted more clearly.
Response 07: We thank the reviewer for these excellent suggestions. We have expanded our study limitations section to explicitly discuss the potential impacts of residual confounding (lines 313-315) and proxy-related measurement error (315-321).
Comment 08: Conclusion
The conclusion effectively summarizes the main findings and reinforces the importance of addressing both awareness and affordability. The recommendation for integrated interventions is appropriate and aligns with the study results.

	However, the conclusion could be strengthened by providing more specific and actionable policy recommendations. Additionally, the language should be slightly moderated to avoid implying causality, given the observational nature of the study.<br>Response 08: We thank the reviewer for this constructive feedback. We have revised the Conclusion to provide specific, actionable policy recommendations.<br>Comment 09: References<br>The reference list is comprehensive and includes relevant and recent literature. The citations are generally appropriate and support the arguments made in the manuscript. However, minor inconsistencies in formatting should be corrected to ensure compliance with journal guidelines.<br>Response 09: We appreciate the reviewer's comment. We have thoroughly reviewed the reference list and corrected all formatting inconsistencies.<br>Comment 10: Language and Writing Quality<br>The manuscript is generally well-written and understandable. However, there are several grammatical errors, typographical issues, and inconsistencies in terminology. Some sentences are overly long or repetitive. Professional language editing is recommended to improve clarity and readability.<br>Response 10: We thank the reviewer for this note. The entire manuscript has undergone comprehensive proofreading and professional language editing to eliminate grammatical errors, fix typographical issues, and break down overly long or repetitive sentences.
**Additional Information:**	
**Question**	**Response**
**Financial Disclosure**<br><br>Enter a financial disclosure statement that describes the sources of funding for the work included in this submission. Review the submission guidelines for detailed requirements. View published research articles from *PLOS ONE* for specific examples.<br><br>This statement is required for submission and **will appear in the published article** if the submission is accepted. Please make sure it is accurate.<br><br>**Funded studies**<br>Enter a statement with the following details:<br>• Initials of the authors who received each award<br>• Grant numbers awarded to each author<br>• The full name of each funder<br>• URL of each funder website<br>• Did the sponsors or funders play any role in the study design, data collection and analysis, decision to publish, or preparation of the manuscript?<br><br>Did you receive funding for this work?	The author(s) received no specific funding for this work.

Powered by Editorial Manager® and ProduXion Manager® from Aries Systems Corporation

**Competing Interests**<br><br>Use the instructions below to enter a competing interest statement for this submission. On behalf of all authors, disclose any competing interests that could be perceived to bias this work—acknowledging all financial support and any other relevant financial or non-financial competing interests.<br><br>This statement is required for submission and **will appear in the published article** if the submission is accepted. Please make sure it is accurate and that any funding sources listed in your Funding Information later in the submission form are also declared in your Financial Disclosure statement.<br><br>View published research articles from *PLOS ONE* for specific examples.<br><br>**NO authors have competing interests**<br><br>Enter: *The authors have declared that no competing interests exist.*<br><br>**Authors with competing interests**<br><br>Enter competing interest details beginning with this statement:<br><br>*I have read the journal's policy and the authors of this manuscript have the following competing interests: [insert competing interests here]*<br><br>* typeset	None declared
**Data Availability**<br><br>Authors are required to make all data underlying the findings described fully available, without restriction, and from the time of publication. PLOS allows rare exceptions to address legal and ethical concerns. See the PLOS Data Policy and FAQ for detailed information.	Yes - all data are fully available without restriction

A Data Availability Statement describing where the data can be found is required at submission. Your answers to this question constitute the Data Availability Statement and **will be published in the article**, if accepted.<br><br>**Important:** Stating 'data available on request from the author' is not sufficient. If your data are only available upon request, select 'No' for the first question and explain your exceptional situation in the text box.<br><br>Do the authors confirm that all data underlying the findings described in their manuscript are fully available without restriction?	
Additional data availability information:	
**Ethics Statement**<br><br>Enter an ethics statement for this submission. This statement is required if the study involved:<br><br>• Human participants<br>• Human specimens or tissue<br>• Vertebrate animals or cephalopods<br>• Vertebrate embryos or tissues<br>• Field research<br><br>Write "N/A" if the submission does not require an ethics statement.<br><br>General guidance is provided below. Consult the submission guidelines for detailed instructions. **Make sure that all information entered here is included in the Methods section of the manuscript.**	Not applicable. As the study utilized publicly available and anonymized secondary data, it does not require additional ethical approval.

**Format for specific study types**<br><br>**Human Subject Research (involving human participants and/or tissue)**<br>• Give the name of the institutional review board or ethics committee that approved the study<br>• Include the approval number and/or a statement indicating approval of this research<br>• Indicate the form of consent obtained (written/oral) or the reason that consent was not obtained (e.g. the data were analyzed anonymously)<br><br>**Animal Research (involving vertebrate animals, embryos or tissues)**<br>• Provide the name of the Institutional Animal Care and Use Committee (IACUC) or other relevant ethics board that reviewed the study protocol, and indicate whether they approved this research or granted a formal waiver of ethical approval<br>• Include an approval number if one was obtained<br>• If the study involved *non-human primates*, add *additional details* about animal welfare and steps taken to ameliorate suffering<br>• If anesthesia, euthanasia, or any kind of animal sacrifice is part of the study, include briefly which substances and/or methods were applied<br><br>**Field Research**<br><br>Include the following details if this study involves the collection of plant, animal, or other materials from a natural setting:<br>• Field permit number<br>• Name of the institution or relevant body that granted permission	
Data Availability<br><br>Before publication, Authors are required to make fully available and without restriction all data underlying their findings. Sharing the data underlying your findings increases the visibility, credibility, and impact of your work, while supporting transparency and future scientific discovery. Please see our PLOS Data Policy page for detailed information on this policy.</p><br>A Data Availability	The datasets analyzed during the current study are available on the MICS program website, https://microdata.worldbank.org/index.php/catalog/MICS/

Statement, detailing where the data can be accessed, is required at first submission. Insert your Data Availability Statement in the box below.</p><br><br>Please see the data reporting section of our submission guidelines for instructions on what you need to include in your Data Availability Statement. Examples of acceptable Data Availability Statements are included here for reference:</p><br> <br> - The minimal data set [and accompanying code, where generated] is available at [repository name] via [DOI hyperlink] <br> - No data was generated by this study. The following existing data sources were used: [dataset name] from [dataset location] available via [web address or DOI hyperlink]. <br> - For Study Protocols: No datasets were generated or analysed during the current study. All relevant data from this study will be made available upon study completion. <br> <br><br><i>PLOS allows rare exemptions to address legal and ethical concerns. If you have legal or ethical restrictions, please use the box below to detail these in full sentences for the Journal team to consider.</i></p><br><br>This statement will be typeset if the manuscript is accepted for publication.</p>	

Manuscript

Click here to access/download;Manuscript;MHM_Manuscript_11June2026_Cl

Awareness or Affordability: What matters the most in ensuring menstrual hygiene?

Mohammad Hridoy Patwary[1]; Md Tazvir Amin[1*]; Sumaiya Nusrat[2]; Md Mahabubur Rahman[1]

Health Systems and Population Studies Division, icddr,b, GPO Box 128, Dhaka 1212, Bangladesh[1]

Maternal and Child Health Division, icddr,b., GPO Box 128, Dhaka 1212, Bangladesh[2]

Corresponding author[*]

E-mail: tazvir.amin@icddrb.org; tamin@isrt.ac.bd

Abstract

Background

Ensuring menstrual hygiene remains a critical public health challenge, particularly in resource-limited settings. Both affordability and awareness are considered to play a crucial role in ensuring menstrual hygiene. However, the interactive effect of awareness and affordability remain unclear, particularly in the context of Bangladesh. To address this gap, this study examines the interplay between awareness and affordability in determining the use of sanitary napkins in Bangladesh.

Methods

We used nationally representative data from the 2019 Bangladesh Multiple Indicator Cluster Survey (MICS). Type of material used by women during their last menstruation was used as the outcome variable. Based on literature, individual educational attainment and household wealth index were used as sociodemographic proxies for awareness regarding menstrual hygiene management and affordability to buy sanitary napkins, respectively. A generalized linear model (GLM) with a logit link function was used to estimate adjusted odds ratios (AORs). Then using the fitted model, marginal probabilities of sanitary napkin use were estimated for subgroups defined by combinations of wealth index and educational attainment.

Findings

The national prevalence of sanitary napkin use was 23.4%. Odds of sanitary napkin use was 4.5 times higher (AOR=4.50, 95% CI: 3.28–6.16) among women with secondary or higher education compared to those with no formal education. Similarly, women from the upper three wealth quintiles had 2.3 times higher odds (AOR=2.30, 95% CI: 1.58–3.34) of using sanitary napkins compared to those in the lowest two quintiles. Women with secondary or higher education from lower wealth quintiles were more likely to use sanitary napkins than with pre-primary or no education from wealthier households (marginal probability: 15.46% vs 9.25%).

Conclusion

Both awareness and affordability play a critical role in determining menstrual hygiene practices among Bangladeshi women. While financial constraints remain a key barrier, the findings suggest that improving awareness could significantly enhance hygienic menstrual practices, even among lower-socioeconomic groups. Policies should prioritize interventions for improving awareness of menstrual hygiene management.

Introduction

Menstruation is a natural physiological process that generally indicates the onset of reproductive lifespan for a girl, characterized by the regular discharge of blood and tissue from the uterus through the vagina every month [1]. The first occurrence of menstruation (aka Menarche) usually happens between the ages of 12 to 15 and represents a transformative phase in the lives of adolescent girls, bringing along new challenges and vulnerabilities [2, 3]. Despite being a natural and essential bodily process, menstruation has been surrounded by taboos, myths, and secrecy in many cultures for a long time, leading to stigma, shame, and silence around the world, especially in low- and middle-income countries (LMICs) [4-6]. This stigma and shame can have significant negative impacts on the emotional well-being and self-esteem of menstruating individuals, particularly young girls as they transition into adolescence [7, 8].

Each month, approximately 1.8 billion girls worldwide experience menstruation, yet millions lack the resources to manage their menstrual hygiene in a dignified and healthy way due to limited affordability, lack of awareness, stigma, taboos, and silence about menstrual hygiene management (MHM) [9, 10]. Inadequate access to appropriate menstrual health resources and supportive environments has been associated with many adverse health outcomes such as lower RTIs, secondary infertility, and abnormal vaginal discharge [11-14]. It also affects the quality of life and performance in schools and workplaces, a significant barrier to women's empowerment and social inclusion [15-17].

Menstrual hygiene management (MHM) has become a global public health concern, emphasizing individuals' practices and actions to maintain cleanliness, dignity, and health during menstruation [18]. MHM is also closely linked with health, social justice, and human rights [18]. MHM has also become a vital focus within the Sustainable Development Goals (SDGs) due to its importance in enhancing women's physical and mental well-being and promoting social inclusion [19]. It involves using proper menstrual hygiene products (i.e., napkins, tampons, dry cloths, menstrual

cups, etc), sanitation facilities (i.e. washing the cloths, drying them in sunlight, disposal of the menstrual product properly, etc.), and healthy behaviors (changing the napkins or cloths regularly at least 4 times a day) during menstruation [19].

Recognizing the impact of inadequate MHM on health, education, and gender equality, various efforts have been undertaken in LMICs to address these challenges. Governments, international organizations, and local NGOs have implemented programs to improve access to menstrual products, sanitation facilities, and menstrual health education. Yet, the pervasive stigma and silence surrounding menstruation in LMICs like Bangladesh obstruct access to rights-based information and services regarding MHM [20]. Around half of the adolescent girls in rural Bangladesh are not very confident about managing their menstrual bleeding, even in their own house, where this percentage in school is less than 10% [20]. Poor menstrual hygiene management also affects their performance in school and overall life [21, 22]. National representative data shows that 30% of school girls experienced an average monthly absence of 2.5 days due to menstruation [23]. Though the average age of menarche in Bangladesh is higher (12.8) than the global average (12.4), only a quarter of adolescent girls know about menstruation before their menarche [23-25]. Only 24.3% of women and adolescent girls in Bangladesh use proper modern absorbents, and fewer than 20% of old cloth users adhere to the recommended cleaning and drying practices [23, 26].

While traditional practices such as using old cloths are deeply rooted in cultural norms and have been passed down across generations, they often lack hygienic standards and are associated with a higher risk of infection and discomfort [27]. Introducing sanitary napkins challenges these long-standing customs and requires not only behavioral change but also sustained access to affordable and acceptable alternatives. This dual requirement of behavioral shift and financial investment highlights the importance of both awareness and affordability in adopting improved menstrual hygiene practices.

While extensive research identifies awareness and affordability as critical determinants of sanitary napkin use, the literature remains fragmented. Prior studies have typically examined the factors in isolation, suggesting that higher education and better economic status are individually associated with improved MHM [26, 28]. However, there is a notable gap in the literature regarding their combined and potentially interactive influence. Specifically, current evidence fails to explain whether high awareness can compensate for low affordability, or if financial constraints

completely nullify the benefits of health literacy. Moreover, most existing studies provide limited insights into regional disparities, despite the fact that menstrual health behaviors and barriers can vary widely by geography, culture, and local infrastructure. Therefore, this study aims to examine how women's awareness and affordability jointly shape the use of sanitary napkins, offering evidence that can inform both national and region-specific strategies to enhance menstrual hygiene management in Bangladesh.

Methods

Data overview

This study utilized data from the 2019 Bangladesh Multiple Indicator Cluster Survey (MICS), conducted by the Bangladesh Bureau of Statistics (BBS) with technical support from UNICEF. The survey employed a two-stage stratified sampling design to collect nationally representative information on the health and well-being of women and children across all districts of Bangladesh, covering both urban and rural areas.

A total of 3,220 primary sampling units (PSUs) and 64,400 households were included in the survey. In the first stage, enumeration areas (EAs) were selected using probability proportional to size. In the second stage, 20 households were randomly selected from each selected EA.

The survey collected detailed information from women of reproductive age (15–49 years). Sampling weights provided in the dataset were applied in all analyses to account for the complex survey design and ensure national representativeness. The dataset used in this study is publicly available (accessed in January, 2023) and de-identified, with no information that could be used to identify individual participants.

Outcome variable

The outcome variable for this study was whether the participant used a sanitary napkin during her last menstruation. The original variable, which included various materials (e.g., cloth, tissue, cotton pads), was recoded into a binary variable: sanitary napkin vs. others. In this study, sanitary "sanitary napkin use" refers to any reported use of purpose-made sanitary pads during the last menstrual period, regardless of whether they were used exclusively or in combination with other materials. Thus, the measure captures reported use rather than sufficiency of access or adequacy of product use throughout the full menstrual cycle. Sanitary napkin use was considered a proxy for proper menstrual hygiene, as they are typically cleaner, safer, and more absorbent than traditional or improvised materials.

Explanatory variables

As individual-level indicators of health awareness and affordability were not available in the dataset, proxies were used to represent these constructs. Educational attainment was employed as a proxy for awareness (knowledge regarding menstrual hygiene management), as higher education is generally associated with better knowledge of health practices [29]. Specifically, formal

schooling enhances a woman's health literacy and her ability to access, interpret, and adopt evidence-based health information, which are core components of menstrual hygiene awareness. Similarly, the household wealth index was used as a proxy for affordability, as it reflects the economic capacity of a household to purchase menstrual hygiene products and related resources. This measure serves as an indicator of a household's disposable income and its ability to prioritize health-related expenditures over basic subsistence needs. Other variables considered in this study were identified from relevant literature from Bangladesh [26], India, Pakistan, and Nepal (Table 1) [30-32]. Individual level factors that can affect use of sanitary napkin included participants age, educational attainment, exposure to media, and status of disability. Household level factors consists of wealth quintile, religious belief of household along with sex and educational attainment of household head. Community level prevalence of sanitary napkin usage, type of residence and division of the participants were included as community level factor. Community-level prevalence of sanitary napkin usage was calculated as the percentage of women in each survey cluster who reported using sanitary napkins during their last menstruation.

Table 1. Description of variables used in the study

Explanatory variable	Categories	Description
**Individual-level factors**		
Age	15-19	Respondent's current age is between 15 and 19 years.
	20-24	Respondent's current age is between 20 and 24 years.
	25-29	Respondent's current age is between 25 and 29 years.
	30-34	Respondent's current age is between 30 and 34 years.
	35-39	Respondent's current age is between 35 and 39 years.
	40-45	Respondent's current age is between 40 and 44 years.
	45+	Respondent's current age is above 45 years.
Educational attainment	Primary or below	No formal education
	Secondary or above	6–10 or more than 10 years of formal schooling
Media Exposure	No media exposure	Respondent does not get any exposure to mass media.
	At least once a week	Get exposure at least once a week from at least one media.
	Almost everyday	Get exposure almost every day from at least one media.
Disability	Yes	Respondent has at least one disability among difficulty seeing, hearing, walking, remembering or concentrating, self-care, and communicating.
	No	Respondent has no such disabilities.
**Household-level factors**		

Wealth Index	Bottom two	Household belongs to the bottom two wealth quantile.
	Upper three	Household belongs to the upper three wealth quantile.
Educational attainment of household head	Pre-primary or none	No formal education
	Primary	1–5 years of formal schooling
	Secondary or above	6–10 or more than 10 years of formal schooling
Sex of household head	Male	Sex of the household head is male.
	Female	Sex of the household head is female.
Religious affiliation	Muslim	Respondent belongs to a Muslim household.
	Non-Muslim	Respondent belongs to a non-Muslim household.
**Community-level factors**		
Community prevalence of sanitary napkin usage	Low prevalence	The community prevalence of sanitary napkins use is below 30%.
	Moderate prevalence	The community prevalence of sanitary napkins use is between 30 to 60%.
	High prevalence	The community prevalence of sanitary napkins use is higher than 60%.
Area of Residence	Rural	Respondent's type of place of residence is rural.
	Urban	Respondent's type of place of residence is urban.
Division	Dhaka	Respondent lives in the Dhaka division
	Barisal	Respondent lives in the Barisal division
	Chattogram	Respondent lives in the Chattogram division
	Khulna	Respondent lives in the Khulna division
	Mymensingh	Respondent lives in the Mymensingh division.
	Rajhshahi	Respondent lives in the Rajshahi division
	Rangpur	Respondent lives in the Rangpur division.
	Sylhet	Respondent lives in the Sylhet division.

Statistical analyses

Univariate analysis was conducted to examine the sample characteristics. The chi-square (χ^2) test was used to assess associations between the outcome variable and explanatory variables. The outcome of interest, use of sanitary napkins, was then modeled using a generalized linear model (GLM) with a logit link function to estimate adjusted odds ratios (AORs) for the study factors. After fitting the model, the marginal probabilities for six groups, defined by combinations of wealth index and individual education level, were estimated using the margins command in Stata (Stata SE V.14, StataCorp).

All analyses accounted for survey weights that were provided in the dataset to ensure representativeness. The spatial map was constructed using R (version 4.1.1). The district-level Shapefile of Bangladesh was accessed from the *bangladesh* package in R [33], which provides open-source geographic boundary data. Maps were generated using the *ggplot2* package (version

4.3.2) without the use of proprietary basemap services such as Google Maps, Google Earth, or Google Street View.

Results

Table 2 presents the demographic characteristics of 46,677 surveyed women aged 15-49 years. Notably, a predominant age group falls within the range of 20-39 years, representing 18.62% of the total sample. Educational attainment reveals a substantial majority, with 60.91% of respondents possessing secondary or higher qualifications. Regarding media exposure, a significant portion, accounting for 57.33% of the sample, reported being exposed to media almost daily. Approximately one-fifth (21.58%) of respondents acknowledged having a disability. Socioeconomic status demonstrates a notable disparity, with 35.41% of individuals falling within the bottom two wealth quantiles and 64.59% within the upper three. An examination of educational attainment within wealth indices reveals intriguing variations. Religious demographics indicate a Muslim majority, comprising 90.24% of the sample, while male-headed households constitute 88.23%. Notably, 72.81% of the surveyed women reported

Table 2. Sample characteristics of the study participants.

	**Distribution**	
	**n (unweighted)**	**% (weighted)**
**Total**	46,677	100
**Age group**		
15-19	4,462	9.56
20-24	8,690	18.62
25-29	8,453	18.11
30-34	8,712	18.66
35-39	7,854	16.83
40-44	5,300	11.35
45-49	3,205	6.87
**Educational attainment**		
Pre-primary or none	7,209	15.44
Primary	11,039	23.65
Secondary or above	28,429	60.91
**Media exposure**		
Not at all	13,767	29.49
Less than once a week	2,233	4.78
At least once a week	3,914	8.39
Almost every day	26,762	57.33
**Disability**		
Not disable	36,593	78.4
Disable	10,075	21.58
**Wealth Index**		

	Distribution	
	**n (unweighted)**	**% (weighted)**
Bottom two	16,530	35.41
Upper three	30,147	64.59
**Educational attainment**		
Pre-primary or none	14,503	31.07
Primary	12,728	27.27
Secondary or above	19,445	41.66
**WI & Educational attainment**		
Pre-primary or none & Bottom two	4,278	9.17
Pre-primary or none & Upper Three	2,931	6.28
Primary & Bottom two	5,444	11.66
Primary & Upper Three	5,595	11.99
Secondary & Bottom two	6,808	14.59
Secondary & Upper three	21,621	46.32
**Religious affiliation**		
Other Religion	4,556	9.76
Muslim	42,121	90.24
**Sex of household head**		
Female	5,492	11.77
Male	41,184	88.23
**Community prevalence of sanitary napkin use**		
Low prevalence	33,821	72.81
Moderate prevalence	10,188	21.93
High prevalence	2,444	5.26
**Area of residence**		
Rural	35,245	75.51
Urban	11,432	24.49
**Division**		
Barisal	2,482	5.32
Chattogram	8,835	18.93
Dhaka	12,114	25.95
Khulna	5,526	11.84
Mymensingh	3,062	6.56
Rajshahi	6,262	13.42
Rangpur	4,966	10.64
Sylhet	3,429	7.35

using sanitary napkins, with the majority residing in rural areas (75.51%). Geographically, Dhaka emerges as the most represented division (25.95%), followed by Chattogram (18.93%).

The distribution of the proportion of women using sanitary napkins across districts in Bangladesh is illustrated in Figure 1. The spatial distribution reveals a generally poor level of sanitary napkin use throughout the country. In only five out of 64 districts does nearly one in three women use sanitary napkins, still a very low proportion. The northern region shows the lowest usage rates, with Lalmonirhat having the lowest proportion, where approximately 6% of women use sanitary napkin.

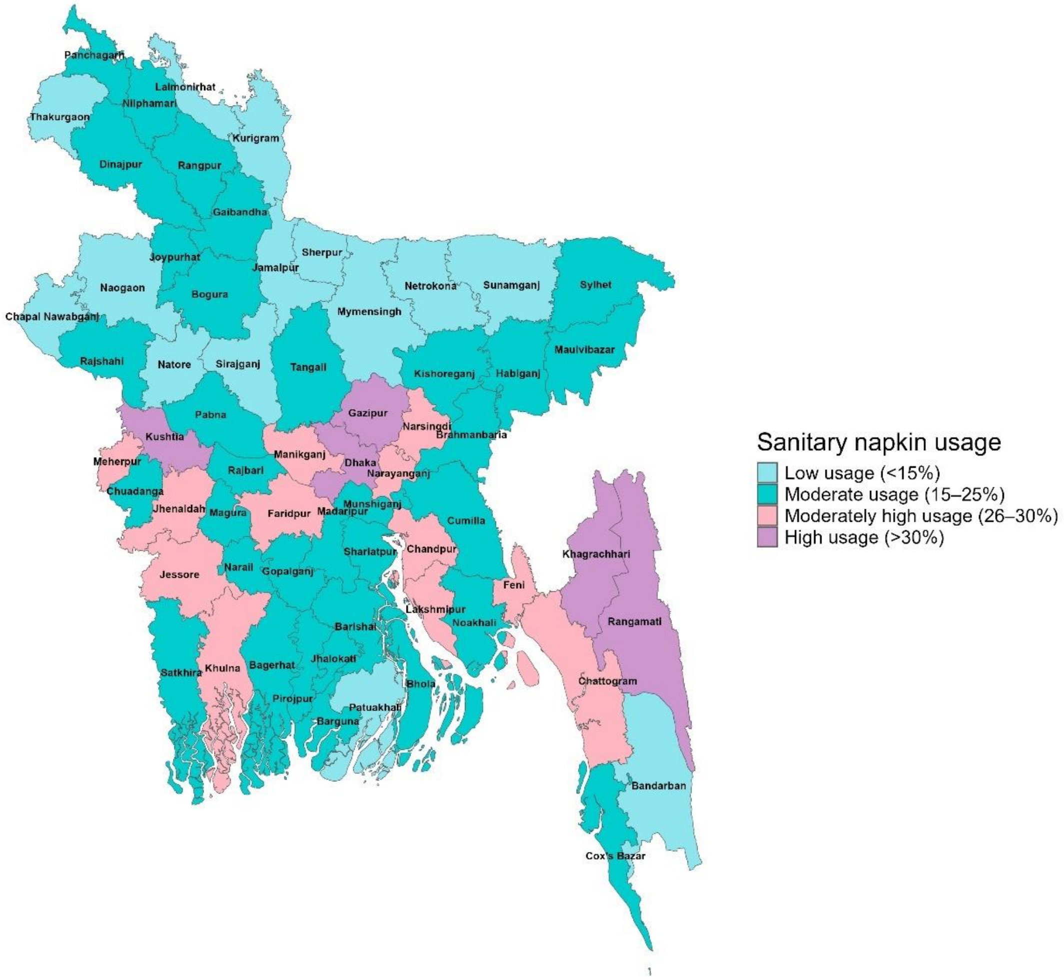

Fig 1. District-wise mapping on the distribution of sanitary napkin use in Bangladesh. (Source of administrative boundaries: *bangladesh* R package (open-source shapefile data). Source of estimates: analysis from BMICS data 2019.)

Sanitary napkin use by education and wealth

The overall prevalence of sanitary napkin usage among women aged 15–49 years was 23.4% (Supplementary table 1). Women with secondary or higher education from the bottom two wealth quintiles had a higher prevalence of sanitary napkin use (11.84%) than women from the upper three wealth quintiles who had pre-primary or no education (5.09%) or only primary education (10.82%) (Figure 2).

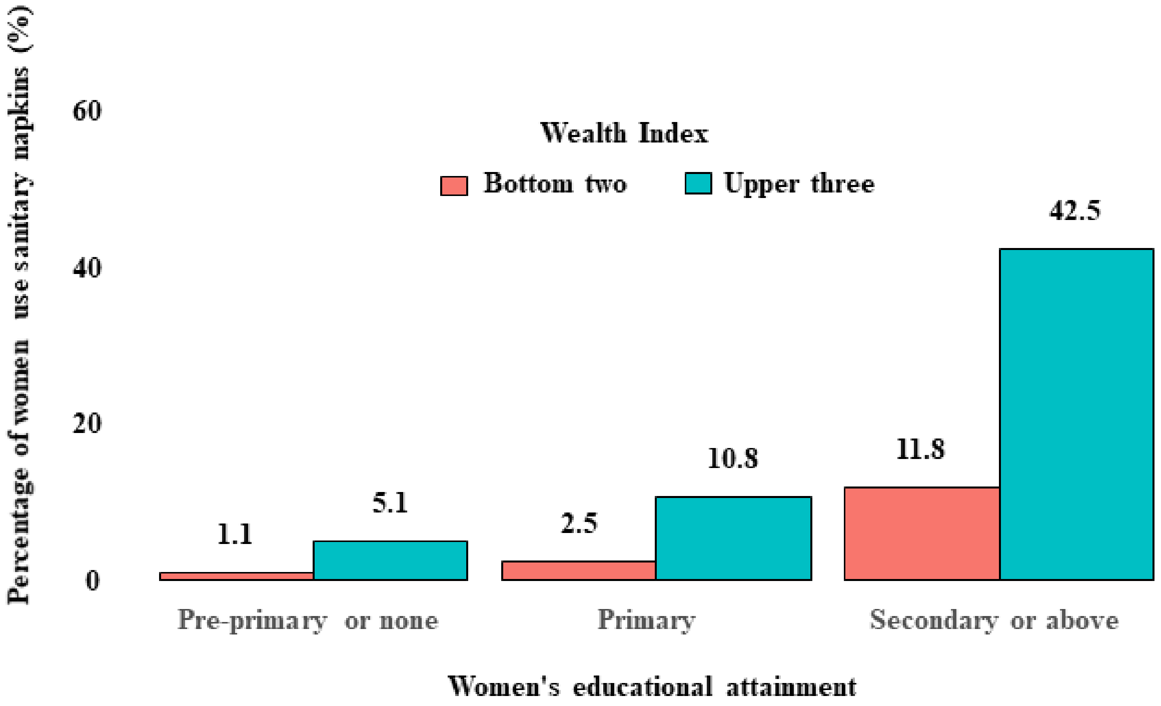

Fig 2. Percentage of women use sanitary napkins over educational attainment and wealth index

Table 3 presents adjusted odds ratios (AOR) with associated p-values and 95% confidence intervals (CI) for the relationship between the type of material used during menstruation and various demographic factors. Notable findings include a higher likelihood of using sanitary napkins in younger age groups, with substantial increases in odds ratios from 25-29 years (AOR=2.57) to 15-19 years (AOR=6.09). Education plays a significant role, with increasing AORs for primary (AOR=1.44) and Secondary or above (AOR=4.5) compared to pre-primary or none. Media exposure shows a positive association, with higher odds for more frequent exposure. Individuals without disabilities have higher odds (AOR=0.86) of using Sanitary Napkins. The wealth index indicates significantly higher odds (AOR=2.3) in the upper three quantiles. Notable variations exist in the odds of sanitary napkin usage across different divisions and areas of residence, with urban residents, Dhaka division, and areas with high community prevalence having higher odds.

Table 3. Association of women's education and wealth with sanitary napkin use

	**AOR**	**p-value**	**95% CI**	
			Lower	Upper
**Educational attainment**				
Pre-primary or none	1 (Ref.)			
Primary	1.44	0.048	1.0	2.05
Secondary or above	4.50	<0.01	3.28	6.16
**Wealth index**				
Bottom two	1 (Ref.)			
Upper three	2.30	<0.01	1.58	3.34
**Education & WI**				
Primary & Upper three	1.03	0.90	0.66	1.59
Secondary or above & Upper three	1.33	0.144	0.91	1.95
**Age**				
45-49	1 (Ref.)			
40-44	1.08	0.389	0.90	1.29
35-39	1.34	<0.01	1.14	1.58
30-34	1.54	<0.01	1.30	1.83
25-29	2.57	<0.01	2.18	3.03
20-24	4.17	<0.01	3.54	4.92
15-19	6.09	<0.01	5.13	7.23
**Media exposure**				
Not at all	1 (Ref.)			
Less than once a week	1.27	<0.01	1.08	1.51
At least once a week	1.42	<0.01	1.25	1.62
Almost every day	1.93	<0.01	1.76	2.11
**Disability**				
Not disable	1 (Ref.)			
Disable	0.86	<0.01	0.80	0.93
**Educational attainment of household head**				
Pre-primary or none	1 (Ref.)			
Primary	1.12	0.03	1.01	1.23
Secondary or above	2.11	<0.01	1.92	2.30
**Religious affiliation**				
Muslim	1 (Ref.)			
Other Religion	1.04	0.454	0.93	1.17
**Sex of Household head**				
Female	1 (Ref.)			
Male	0.84	<0.01	0.76	0.93
**Community prevalence of sanitary napkin usage**				
Low Use	1 (Ref.)			

	AOR	p-value	95% CI	
			Lower	Upper
Moderate Use	1.80	<0.01	1.59	2.03
High Use	3.75	<0.01	3.06	4.59
**Area of residence**				
Rural	1 (Ref.)			
Urban	1.50	<0.01	1.38	1.64
**Division**				
Dhaka	1 (Ref.)			
Barishal	1.15	0.02	1.02	1.29
Chattogram	0.74	<0.01	0.68	0.80
Khulna	0.92	0.06	0.84	1.01
Mymensingh	0.60	<0.01	0.50	0.71
Rajshahi	0.68	<0.01	0.62	0.76
Rangpur	0.67	<0.01	0.60	0.75
Sylhet	0.85	0.051	0.73	1.00
**Intercept**	0.01	<0.01	0.00	0.01

From the fitted regression model, we have calculated the marginal probabilities of sanitary napkin usage for every subgroup created by two variables: educational attainment and wealth quintiles. The national estimates from figure 3 shows that, educational attainment significantly increases the likelihood of using sanitary napking among women aged 15-49 years in Bangladesh. Women with secondary or higher educational attainment from poorer households (bottom two quintiles) had a higher chance of using sanitary napkin compared to women with pre-primary or no educational attainment from a rich household (upper three). This behavibiour was consistent across all divisions.

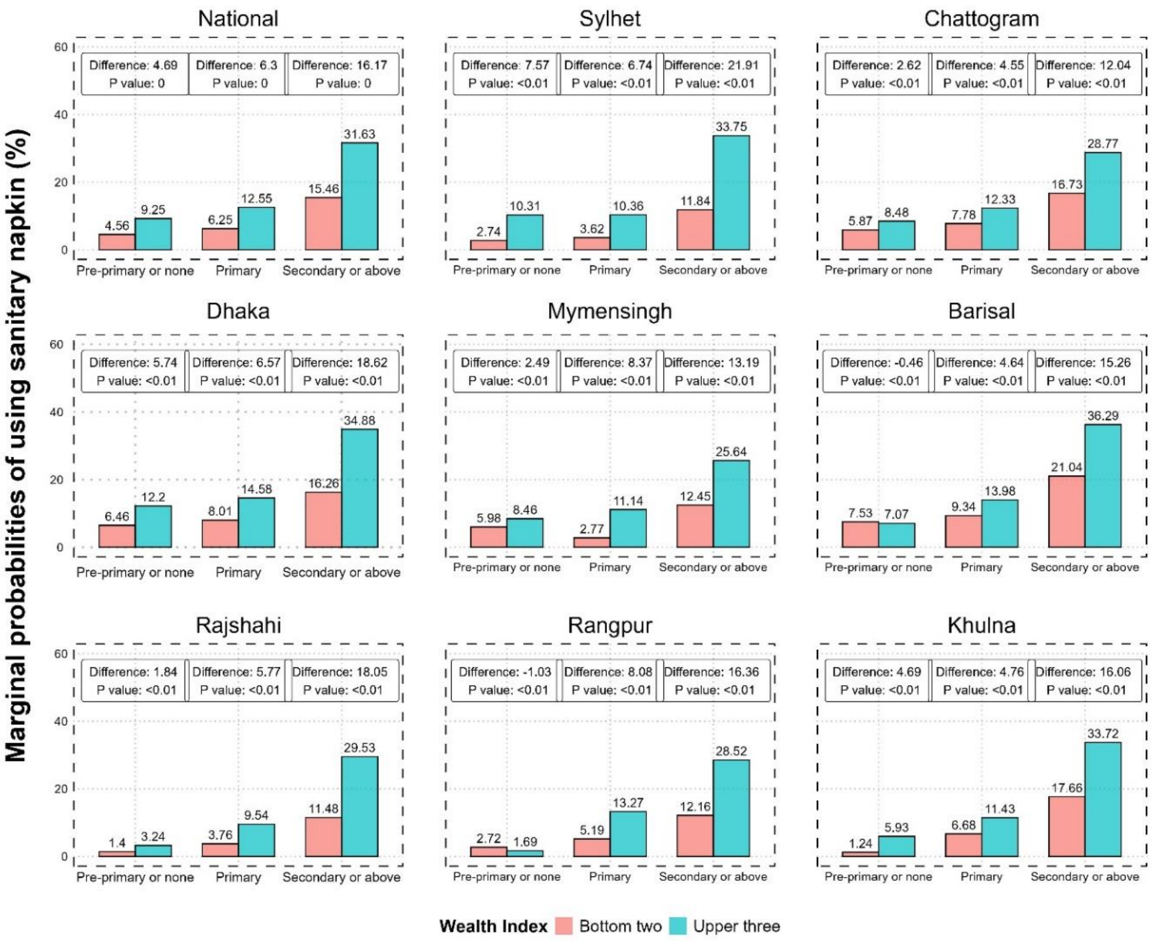

p-value from t-test

Fig 3. Marginal probabilities of sanitary napkin usage by women's education and wealth.

Discussion

This study highlights the significant roles of both awareness and affordability in determining sanitary napkin use among women in Bangladesh. The results suggest that awareness has a particularly strong association with sanitary napkin use compared to affordability. Besides less educated, younger, and disabled women, those living in male-headed or less educated households, with limited media exposure, residing in rural areas or in divisions like Mymensingh and Rangpur, and living in communities with low sanitary napkin use were less likely to use sanitary napkins.

Factors influencing sanitary napkin use

Consistent with prior research, this study identified significant variation in sanitary napkin use across geographic, household, and individual levels [34]. Findings indicates there are significant differences in menstrual hygiene practices across divisions, urban and rural areas, which are in line

with previous studies [17]. This difference is might be due to some unobserved factors, which vary across regions.

At the community-level, prevalence of sanitary napkin use within a community was a significant predictor of individual use, reflecting the role of descriptive norms in shaping health behavior which consistent with Social Cognitive Theory [35]. In Bangladesh, where menstruation remain culturally stigmatized, communities with higher sanitary napkin use likely foster greater openness and awareness, reducing the social cost of adoption. Similar patterns have been observed elsewhere: in India, Sood et al. found that positive social norms significantly improved MHM [36]; in Nepal, Shrestha et al. reported that caste-based taboos limited awareness regarding MHM [37].

At the household-level, women in male-headed households are less likely to use sanitary napkins, consistent with evidence that household gender dynamics and educational level of the household head influence women's health knowledge, autonomy, and access to resources [26]. From the perspective of the Andersen Behavioral Model [38], this finding indicates that household social structure may influence women's access to and use of sanitary napkins.

At the individual-level mass media exposure seen to have a significant role in menstrual hygiene practices which serves as a core source of information on MHM [30]. Women who are exposed to media more frequently are more likely to have better knowledge and use of sanitary products, with those accessing media almost every day showing the highest likelihood. The intersection of menstruation and disability endangers an intricate set of circumstances that can give rise to compounded discrimination and present distinct challenges for menstruating individuals with disabilities [24]. This study also found evidence supporting this phenomenon.

Interplay between awareness and affordability in napkin use

Our findings reveal that both awareness and affordability significantly influence the use of sanitary napkins, underscoring their interdependent roles in ensuring effective MHM. While higher levels of awareness are strongly associated with increased use, this effect is amplified in the presence of affordability, indicating a synergistic relationship between the two factors.

Awareness enhances knowledge of menstruation as a biological process, reduces stigma, and improves attitudes towards the use of hygienic products [39]. Due to lack of awareness, menstrual hygiene is often deprioritized and menstrual products are overlooked in basic WASH and sanitation planning [40]. However, even with increased awareness and affordability, many girls still refrain from using them due to embarrassment and stigma. A cross-sectional survey of adolescent girls in Rajshahi revealed that, factors like embarrassment purchasing pads from male shopkeepers and stigma surrounding menstruation were major barriers to sanitary napkin use [41]. This suggests that socio-cultural taboos pose further barriers to access that persist even when informational and economic constraints are addressed.

However, knowledge alone does not always translate into practice when economic barriers remain. This is particularly relevant in low-income households where affordability and availability is the key barriers [42]. In such settings, even when girls are aware of the benefits of sanitary napkins, they may be forced to rely on unsafe alternatives due to cost constraints.

Action taken to promote sanitary napkins

In Bangladesh, the government recognizes MHM as essential for women's and girls' health and has prioritized awareness and affordability in the National MHM Strategy 2021 [43]. To reduce costs and promote local manufacturing, the government has waived VAT on raw materials for sanitary napkin production. Additionally, the National MHM Coordination Committee has been established to oversee and align MHM efforts across health, education, and social sectors.

Despite these efforts, a 15% VAT on finished sanitary napkin products remains a significant barrier to affordability, which was also reflected by our findings that women from poorer households were less likely to use sanitary napkins. To address this, the strategy calls for revisiting the VAT policy to make napkins more affordable. It also emphasizes improving distribution networks to ensure access in workplaces, schools, healthcare centers, and public spaces, alongside expanding MHM awareness sessions to marginalized and hard-to-reach communities.

Notably, the current NCTB curriculum (after the recent update in 2025) does not adequately address menstruation and menstrual hygiene management, particularly in the subject "Physical Education and Health" for classes 6, 7, and 8[44-46]. These textbooks lack essential information on menstruation, the use of sanitary napkins, and guidance on changing intervals, which are critical for maintaining good menstrual hygiene practices. As a result, many adolescent girls remain uninformed or misinformed about safe and hygienic practices during menstruation. Revising the curriculum to include these crucial topics is imperative for equipping adolescents with the accurate knowledge and practical skills they need to manage menstruation.

On the other hand, non-governmental research organizations have implemented programs to increase awareness about menstruation. One such initiative was the Ritu Programme, funded by the Embassy of the Kingdom of the Netherlands in Bangladesh and implemented from November 2015 to March 2020. The programme aimed to improve menstrual health among girls aged 10–15 in Netrokona, Bangladesh[47]. Through evidence-informed interventions, including school-based menstrual health education, community engagement with parents, and a national-level mass media campaign, the programme significantly boosted menstruation awareness and management. It notably increased girls' confidence and understanding of menstruation, improved hygiene practices (like changing materials more frequently and increased sanitary pad use).

Taken together, the findings underscore that MHM is shaped by a complex interplay of structural, social, and individual determinants. While individual awareness and household affordability are critical, they do not operate in isolation; rather, they are embedded within broader contextual factors such as community norms, media exposure, educational content, and policy frameworks. Therefore, effective menstrual health interventions must adopt a multi-level approach that simultaneously targets these interconnected domains.

Strengths and Limitations of the study

A key strength of this study is the use of nationally representative MICS 2019 data, which ensures broad generalizability. It applies a generalized linear model to estimate adjusted associations and

calculates marginal probabilities to clearly quantify the combined effects of awareness and affordability on sanitary napkin use. By directly comparing subgroups (e.g., educated-poor vs. uneducated-rich), the study provides concrete evidence on the relative influence of awareness and affordability, offering actionable insights for targeted MHM interventions.

This study has several limitations. First, the cross-sectional nature of the data limits the scope of estimating the causal effects. Second, although we adjusted for major socio-demographic covariates, the potential for residual confounding remains from unmeasured factors such as cultural beliefs, familial traditions, or regional product availability. Third, awareness and affordability were measured using proxy indicators, educational attainment and household wealth index, which may not fully capture the complexity of these constructs. Specifically, formal education serves as a proxy for health literacy but does not directly measure specific knowledge about menstrual physiology or hygienic practices. Similarly, the household wealth index reflects overall socio-economic status and asset ownership rather than a woman's individual financial autonomy or her direct purchasing power to buy sanitary products. Forth, self-reported data on menstrual hygiene practices may be subject to social desirability or recall bias. Lastly, certain contextual factors such as cultural norms, school-based education on menstruation, and access to WASH (Water, Sanitation, and Hygiene) facilities were not available in the dataset and could not be accounted for in the analysis. Another limitation is that the survey included only women aged 15–49 years, excluding younger adolescents who also menstruate and may have different hygiene practices.

Conclusion

This study demonstrates that menstrual hygiene management is influenced by interconnected factors at the individual, household, and community levels. Awareness and affordability show significant interaction effects, with awareness emerging as a key driver of sanitary napkin use across wealth quintiles. Therefore, standalone economic or informational campaigns may be incomplete. To be actionable, policymakers should implement integrated menstrual hygiene management interventions, such as incorporating comprehensive reproductive health literacy into the national school curriculum alongside targeted subsidies or tax exemptions on raw materials for sanitary products to lower market prices. These strategies are vital for reducing sub-national disparities and ensuring sustainable, hygienic menstrual practices across diverse socio-economic contexts in Bangladesh

Declarations

Ethics approval and consent to participate

The BMICS 2019 provides de-identified data, which cannot be used to identify any study participants. Regarding the ethics approval, the BMICS 2019 protocol was approved by the technical committee of the Government of Bangladesh, led by the Bangladesh Bureau of Statistics

(BBS). As the study utilized BMICS 2019 as a secondary data source, it does not require additional ethical approval, nor does it have any information to identify study participants.

Consent for publication

Not applicable.

Availability of data and materials

The datasets analyzed during the current study are available on the MICS program website, https://microdata.worldbank.org/index.php/catalog/MICS/

Competing interests

None declared.

Funding

The authors received no specific grant funding for this research.

Authors' contributions

MHP, MTA, and MMR conceptualized the study. MHP and MMR performed data curation and conducted the formal statistical analysis. MHP, MTA, SNE, and MMR contributed to the writing of the original draft. MMR supervised the study. All authors read and approved the final version of the manuscript.

Acknowledgments

The authors would like to thank UNICEF and the Bangladesh Bureau of Statistics (BBS) for providing access to the 2019 Bangladesh Multiple Indicator Cluster Survey (MICS) dataset. icddr,b acknowledges the Government of the People’s Republic of Bangladesh and Canada for providing core/unrestricted support.

References

1. Habeeb, S., *Menstruation*, in *Encyclopedia of Sexual Psychology and Behavior*, T.K. Shackelford, Editor. 2023, Springer International Publishing: Cham. p. 1–11.
2. Critchley, H.O.D., et al., *Physiology of the Endometrium and Regulation of Menstruation.* Physiol Rev, 2020. **100**(3): p. 1149–1179.
3. Likis, K.D.S.a.F.E., *Women’s Gynecologic Health*. Vol. Second Edition. 2013.
4. Dutta, D., et al., *Supporting the rights of girls and women through Menstrual Hygiene Management (MHM) in the East Asia and Pacific Region: Realities, progress and opportunities.* Bangkok, Thailand: UNICEF East Asia and Pacific Regional Office (EAPRO), 2016.
5. Sommer, M. and M. Sahin, *Overcoming the taboo: advancing the global agenda for menstrual hygiene management for schoolgirls.* American journal of public health, 2013. **103**(9): p. 1556–1559.

6. Korir, E., F.N. Okwara, and G. Okumbe, *Menstrual hygiene management practices among primary school girls from a pastoralist community in Kenya: a cross sectional survey.* Pan African Medical Journal, 2018. **31**(1).
7. Olson, M.M., et al., *The persistent power of stigma: A critical review of policy initiatives to break the menstrual silence and advance menstrual literacy.* PLOS Global Public Health, 2022. **2**(7): p. e0000070.
8. Maulingin-Gumbaketi, E., et al., *Socio-cultural implications for women's menstrual health in the Pacific Island Countries and Territories (PICTs): a scoping review.* Reproductive health, 2022. **19**(1): p. 128.
9. UNICEF, *Menstrual hygiene | UNICEF. Avilable: https://www.unicef.org/wash/menstrual-hygiene.* 2023.
10. UNICEF. *Menstrual hygiene: Gender inequality, cultural taboos and poverty can cause menstrual health needs to go unmet.* . Available from: https://www.unicef.org/wash/menstrual-hygiene.
11. Ali, T.S., N. Sami, and A.K. Khuwaja, *Are unhygienic practices during the menstrual, partum and postpartum periods risk factors for secondary infertility?* J Health Popul Nutr, 2007. **25**(2): p. 189–94.
12. Anand, E., J. Singh, and S. Unisa, *Menstrual hygiene practices and its association with reproductive tract infections and abnormal vaginal discharge among women in India.* Sex Reprod Healthc, 2015. **6**(4): p. 249–54.
13. Das, P., et al., *Menstrual Hygiene Practices, WASH Access and the Risk of Urogenital Infection in Women from Odisha, India.* PLoS One, 2015. **10**(6): p. e0130777.
14. Torondel, B., et al., *Association between unhygienic menstrual management practices and prevalence of lower reproductive tract infections: a hospital-based cross-sectional study in Odisha, India.* BMC Infect Dis, 2018. **18**(1): p. 473.
15. Hennegan, J. and P. Montgomery, *Do Menstrual Hygiene Management Interventions Improve Education and Psychosocial Outcomes for Women and Girls in Low and Middle Income Countries? A Systematic Review.* PLoS One, 2016. **11**(2): p. e0146985.
16. Krenz, A. and H. Strulik, *The impact of menstruation hygiene management on work absenteeism of women in Burkina Faso.* Econ Hum Biol, 2021. **43**: p. 101067.
17. Tegegne, T.K. and M.M. Sisay, *Menstrual hygiene management and school absenteeism among female adolescent students in Northeast Ethiopia.* BMC Public Health, 2014. **14**: p. 1118.
18. Sommer, M., et al., *A Time for Global Action: Addressing Girls' Menstrual Hygiene Management Needs in Schools.* PLoS Med, 2016. **13**(2): p. e1001962.
19. Sommer, M., et al., *How addressing menstrual health and hygiene may enable progress across the Sustainable Development Goals.* Glob Health Action, 2021. **14**(1): p. 1920315.
20. Hennegan, J. and L. Sol, *Confidence to manage menstruation at home and at school: findings from a cross-sectional survey of schoolgirls in rural Bangladesh.* Culture, health & sexuality, 2020. **22**(2): p. 146–165.
21. Ahmed, M.S., et al., *Association between menstrual hygiene management and school performance among the school-going girls in rural Bangladesh.* Adolescents, 2021. **1**(3): p. 335–347.
22. Hasan, M., et al., *Menstrual hygiene practices and school absenteeism among adolescent girls in Bangladesh: A cross-sectional study. Popul Med 2021; 3: 8*. 2021.

23. Khan, S., et al., *Taking measures before opportunities bleed away: Current knowledge and policy gaps in menstrual hygiene management among adolescent girls in Bangladesh.* United States Agency for International Development (USAID), 2022.
24. A. Lacroix, H.G., K. Shumway, and M. Langaker. *Physiology, Starting*. Available from: https://gifsided.com/female-reproductive-system-questions-and-answers-pdf.
25. Sibli, M.S., et al., *Analysis of the average menopause age of Bangladeshi women: A public health perspective.* World Journal of Advanced Research and Reviews, 2022. **16**(3): p. 231–235.
26. Afiaz, A. and R.K. Biswas, *Awareness on menstrual hygiene management in Bangladesh and the possibilities of media interventions: using a nationwide cross-sectional survey.* BMJ Open, 2021. **11**(4): p. e042134.
27. Sumpter, C. and B. Torondel, *A systematic review of the health and social effects of menstrual hygiene management.* PLoS One, 2013. **8**(4): p. e62004.
28. Mondal, B.K., et al., *Practices and effects of menstrual hygiene management in rural Bangladesh.* 2017.
29. Jejeebhoy, S.J., *Women's education, autonomy, and reproductive behaviour: Experience from developing countries*. 1995: Oxford University Press.
30. Garg, S., et al., *Menstrual hygiene management and its determinants among adolescent girls in low-income urban areas of Delhi, India: a community-based study.* Osong Public Health Res Perspect, 2022. **13**(4): p. 273–281.
31. Wasan, Y., et al., *Practices and predictors of menstrual hygiene management material use among adolescent and young women in rural Pakistan: A cross-sectional assessment.* J Glob Health, 2022. **12**: p. 04059.
32. Bhusal, C.K., *Practice of Menstrual Hygiene and Associated Factors among Adolescent School Girls in Dang District, Nepal.* Adv Prev Med, 2020. **2020**: p. 1292070.
33. *Mahmud, J. (2022). bangladesh: Provides Easy Access to 'Bangladesh' Demographic and Health Survey Data (Version 1.0.0) [Computer software]. https://CRAN.R-project.org/package=bangladesh.*
34. Chauhan, S., et al., *Examining the predictors of use of sanitary napkins among adolescent girls: A multi-level approach.* PLOS ONE, 2021. **16**(4): p. e0250788.
35. Zhai, S., et al., *Analysis, evaluation, and reformulation of social cognitive theory: Toward parent-child shared management in sleep health.* J Pediatr Nurs, 2023. **73**: p. e65–e74.
36. Sood, S., et al., *Measuring the effectiveness of communication programming on menstrual health and hygiene management (MHM) social norms among adolescent girls in India.* Glob Public Health, 2021. **16**(4): p. 578–589.
37. Khanal, G., et al., *Menstruation hygiene management among secondary school students of Chitwan, Nepal:a cross-sectional study.* BMC Women's Health, 2023. **23**(1): p. 395.
38. Alkhawaldeh, A., et al., *Application and Use of Andersen's Behavioral Model as Theoretical Framework: A Systematic Literature Review from 2012-2021.* Iran J Public Health, 2023. **52**(7): p. 1346–1354.
39. Sommer, M., et al., *Comfortably, Safely, and Without Shame: Defining Menstrual Hygiene Management as a Public Health Issue.* Am J Public Health, 2015. **105**(7): p. 1302–11.
40. Mahon, T. and M. Fernandes, *Menstrual hygiene in South Asia: a neglected issue for WASH (water, sanitation and hygiene) programmes.* Gender & Development, 2010. **18**(1): p. 99–113.

41. Ha, M.A.T. and M.Z. Alam, *Menstrual hygiene management practice among adolescent girls: an urban-rural comparative study in Rajshahi division, Bangladesh.* BMC Womens Health, 2022. **22**(1): p. 86.
42. Dasgupta, A. and M. Sarkar, *Menstrual Hygiene: How Hygienic is the Adolescent Girl?* Indian J Community Med, 2008. **33**(2): p. 77–80.
43. *Government of Bangladesh. (2021). National Menstrual Hygiene Management (MHM) Strategy 2021. Ministry of Women and Children Affairs. Available at: https://psb.gov.bd/policies/nmhmsen.pdf.*
44. *National Curriculum and Textbook Board (NCTB). (2024). Physical Education & Health, Class 6. Dhaka: National Curriculum and Textbook Board.*
45. *National Curriculum and Textbook Board (NCTB). (2024). Physical Education & Health, Class 7. Dhaka: National Curriculum and Textbook Board.*
46. *National Curriculum and Textbook Board (NCTB). (2024). Physical Education & Health, Class 8. Dhaka: National Curriculum and Textbook Board.*
47. *Simavi, RedOrange, TNO, BNPS, & DORP. (2020). Ritu programme – Improving menstrual health of girls in Bangladesh: Final report 2016-2020.*

Click here to access/download
Supporting Information
MHM_Supplementary.pdf

Awareness or Affordability: What matters the most in ensuring menstrual hygiene ~~management~~?

Mohammad Hridoy Patwary[1]~~[1]~~; Md Tazvir Amin[1]~~[2]~~*; Sumaiya Nusrat~~ Esha~~[2]~~[1]~~; Md Mahabubur Rahman[1]~~[2]~~

~~Maternal and Child Health Division, icddr,b., GPO Box 128, Dhaka 1000, Bangladesh[1]~~

Health Systems and Population Studies Division, icddr,b, GPO Box 128, Dhaka 1212~~000~~, Bangladesh[1]~~[2]~~

Maternal and Child Health Division, icddr,b., GPO Box 128, Dhaka 1212,~~1000,~~ Bangladesh[2]~~[1]~~

Corresponding author*

E-mail: tazvir.amin@icddrb.org; tamin@isrt.ac.bd

Formatted: Highlight

Formatted: Not Highlight

Abstract

Background

Ensuring menstrual hygiene ~~management (MHM)~~ remains a critical public health challenge, particularly in resource-limited settings. Both ~~While~~ affordability and awareness are considered ~~is often cited as a primary barrier to sanitary napkin use, awareness regarding menstrual hygiene~~to ~~may also~~ play a crucial role in ensuring menstrual hygiene. However, the interactive effect of awareness and affordability remain unclear, particularly in the context of Bangladesh. To address this gap, t~~T~~his study examines the interplay between awareness and affordability in determining the use of sanitary napkins in Bangladesh.

Methods

We used nationally representative data from the 2019 Bangladesh Multiple Indicator Cluster Survey (MICS). Type of material used by women during their last menstruation was used as the outcome variable. Based on literature, ~~where~~ individual educational attainment and household wealth index were used as sociodemographic proxies for awareness regarding menstrual hygiene management and affordability to buy sanitary napkins, respectively. A generalized linear model (GLM) with a logit link function was used to estimate adjusted odds ratios (AORs). Then using the fitted model, marginal probabilities of sanitary napkin use were ~~calculated~~ estimated for subgroups defined by combinations of wealth index and educational attainment.

Findings

The national prevalence of sanitary napkin use was 23.4%. Odds of sanitary napkin use was ~~The likelihood of sanitary napkin use was significantly associated with both awareness and affordability. Women with secondary or higher education had~~ 4.5 times higher ~~more odds~~ (AOR=4.50, 95% CI: 3.28–6.16) among women with secondary or higher education ~~of using~~

~~sanitary napkins~~ compared to those with no formal education. Similarly, women from the upper three wealth quintiles had 2.3 times higher odds (AOR=2.30, 95% CI: 1.58–3.34) of using sanitary napkins compared to those in the lowest two quintiles. Women with secondary or higher education from lower wealth quintiles were more likely to use sanitary napkins than~~ their counterparts~~ with pre-primary or no education from wealthier households (marginal probability: 15.46% vs 9.25%).

Conclusion

Both awareness and affordability play a critical role in determining menstrual hygiene practices among Bangladeshi women. While financial constraints remain a key barrier, the findings suggest that improving awareness could significantly enhance hygienic menstrual practices~~MHM outcomes~~, even among lower-socioeconomic~~income~~ groups. Policies should prioritize ~~integrated~~ interventions for improving awareness of menstrual hygiene management~~that address both economic and informational barriers to ensure equitable access to sanitary products~~.

Introduction

Menstruation is a natural physiological process that generally indicates the onset of reproductive lifespan for a girl, characterized by the regular discharge of blood and tissue from the uterus through the vagina every month [1]. The first occurrence of menstruation (aka Menarche) usually happens between the ages of 12 to 15 and represents a trans~~-~~formative phase in the lives of adolescent girls, bringing along new challenges and vulnerabilities [2, 3]. Despite being a natural and essential bodily process, menstruation has been surrounded by taboos, myths, and secrecy in many cultures for a long time, leading to stigma, shame, and silence around the world, especially in low- and middle-income countries (LMICs) [4-6]. This stigma and shame can have significant negative impacts on the emotional well-being and self-esteem of menstruating individuals, particularly young girls as they transition into adolescence [7, 8].

Each month, approximately 1.8 billion girls worldwide experience menstruation, yet millions lack the resources to manage their menstrual hygiene in a dignified and healthy way due to limited affordability, lack of awareness, stigma, taboos, and silence about menstrual hygiene management (MHM) [9, 10]. Inadequate access to appropriate menstrual health resources and supportive environments has been ~~This poor management of menstrual hygiene is~~ associated with many adverse health outcomes such as lower RTIs, secondary infertility, and abnormal vaginal discharge [11-14]. It also affects the quality of life and performance in schools and workplaces, a significant barrier to women's empowerment and social inclusion [15-17].

Menstrual hygiene management (MHM) has become a global public health concern, emphasizing individuals' practices and actions to maintain cleanliness, dignity, and health during menstruation [18]. MHM is also closely linked with health, social justice, and human rights [18]. MHM has also become a vital focus within the Sustainable Development Goals (SDGs) due to its importance in enhancing women's physical and mental well-being and promoting social inclusion [19]. It involves using proper menstrual hygiene products (i.e., napkins, tampons, dry cloth~~e~~s, menstrual cups, etc), sanitation facilities (i.e. washing the cloth~~e~~s, drying them in sunlight, disposal of the menstrual product properly, etc.), and healthy behaviors (changing the napkins or cloth~~e~~s regularly at least 4 times a day) during menstruation [19].

Recognizing the impact of inadequate MHM on health, education, and gender equality, various efforts have been undertaken in LMICs to address these challenges. Governments, international organizations, and local NGOs have implemented programs to improve access to menstrual products, sanitation facilities, and menstrual health education. Yet, the pervasive stigma and silence surrounding menstruation in LMICs like Bangladesh obstruct access to rights-based information and services regarding MHM [20]. Around half of the adolescent girls in rural Bangladesh are not very confident about managing their menstrual bleeding, even in their own house, where this percentage in school is less than 10% [20]. Poor menstrual hygiene management also affects their performance in school and overall life [21, 22]. National representative data shows that 30% of school girls experienced an average monthly absence of 2.5 days due to menstruation [23]. Though the average age of menarche in Bangladesh is higher (12.8) than the global average (12.4), only a quarter of adolescent girls know about menstruation before their menarche [23-25]. Only 24.3% of women and adolescent girls in Bangladesh use proper modern absorbents, and fewer than 20% of old cloth users adhere to the recommended cleaning and drying practices [23, 26].

While traditional practices such as using old ~~cloths~~cloths are deeply rooted in cultural norms and have been passed down across generations, they often lack hygienic standards and are associated with a higher risk of infection and discomfort [27]. Introducing sanitary napkins challenges these long-standing customs and requires not only behavioral change but also sustained access to affordable and acceptable alternatives. This dual requirement of behavioral shift and financial investment highlights the importance of both awareness and affordability in adopting improved menstrual hygiene practices.

While extensive a growing body of research identifies awareness and affordability has acknowledged these two factors as critical determinants of sanitary napkin use, the literature remains fragmented. Previous Prior studies have typically examined the effects of either awareness or affordabilityfactors in isolation, suggesting that higher education and better economic status are individually associated with improved MHM [26, 28]. However, there is a notable gap in the literature regarding their combined and potentially interactive influence. Specifically, current evidence fails to explain whether high awareness can compensate for low little is known about how awareness and affordability, or if financial constraints completely nullify the benefits of health literacy intersect to influence behavior in diverse socioeconomic contexts across Bangladesh. Addressing this gap is crucial for designing effective interventions. Without understanding how these two dimensions reinforce or offset each other, policy efforts risk being incomplete or misdirected. Moreover, most existing studies provide limited insights into regional disparities, despite the fact that menstrual health behaviors and barriers can vary widely by geography, culture, and local infrastructure. Therefore, this study aims to examine how women's awareness and affordability jointly shape the use of sanitary napkins, offering evidence that can inform both national and region-specific strategies to enhance menstrual hygiene management in Bangladesh.

Moreover, most existing studies provide limited insights into regional disparities, despite the fact that menstrual health behaviors and barriers can vary widely by geography, culture, and local infrastructure. Therefore, this study aims to examine how women's awareness and affordability jointly shape the use of sanitary napkins, offering evidence that can inform both national and region-specific strategies to enhance menstrual hygiene management in Bangladesh.

Formatted: Font color: Auto

Methods

Data overview

This study utilized data from the 2019 Bangladesh Multiple Indicator Cluster Survey (MICS), conducted by the Bangladesh Bureau of Statistics (BBS) with technical support from UNICEF. The survey employed a two-stage stratified sampling design to collect nationally representative information on the health and well-being of women and children across all districts of Bangladesh, covering both urban and rural areas.

A total of 3,220 primary sampling units (PSUs) and 64,400 households were included in the survey. In the first stage, enumeration areas (EAs) were selected using probability proportional to size. In the second stage, 20 households were randomly selected from each selected EA.

The survey collected detailed information from women of reproductive age (15–49 years). Sampling weights provided in the dataset were applied in all analyses to account for the complex survey design and ensure national representativeness. The dataset used in this study is publicly available (accessed in January, 2023) and de-identified, with no information that could be used to identify individual participants.

Outcome variable

The outcome variable for this study was whether the participant used a sanitary napkin during her last menstruation. The original variable, which included various materials (e.g., cloth, tissue, cotton pads), was recoded into a binary variable: sanitary napkin vs. others. In this study, sanitary “sanitary napkin use” refers to any reported use of purpose-made sanitary pads during the last menstrual period, regardless of whether they were used exclusively or in combination with other materials. Thus, the measure captures reported use rather than sufficiency of access or adequacy of product use throughout the full menstrual cycle. Sanitary napkin use was considered a proxy for proper menstrual hygiene, as they are typically cleaner, safer, and more absorbent than traditional or improvised materials.

Explanatory variables

As individual-level indicators of health awareness and affordability were not available in the dataset, proxies were used to represent these constructs. Educational attainment was employed as a proxy for awareness (knowledge regarding menstrual hygiene management), as higher education is generally associated with better knowledge of health practices [29]. Specifically, formal schooling enhances a woman’s health literacy and her ability to access, interpret, and adopt evidence-based health information, which are core components of menstrual hygiene awareness. Similarly, the household wealth index was used as a proxy for affordability, as it reflects the economic capacity of a household to purchase menstrual hygiene products and related resources. This measure serves as an indicator of a household’s disposable income and its ability to prioritize health-related expenditures over basic subsistence needs. Other variables considered in this study were identified from relevant literature from Bangladesh [26], India, Pakistan, and Nepal (Table 1) [30-32]. Individual level factors that can affect use of sanitary napkin included participants age, educational attainment, exposure to media, and status of disability. Household level factors consists of wealth quintile, religious belief of household along with sex and educational attainment of household head. Community level prevalence of sanitary napkin usage, type of residence and division of the participants were included as community level factor. Community-level prevalence of sanitary napkin usage was calculated as the percentage of women in each survey cluster who reported using sanitary napkins during their last menstruation.

Table 1. Description of variables used in the study

Explanatory variable	Categories	Description
**Individual-level factors**		
Age	15-19	Respondent's current age is between 15 and 19 years.

	20-24	Respondent's current age is between 20 and 24 years.
	25-29	Respondent's current age is between 25 and 29 years.
	30-34	Respondent's current age is between 30 and 34 years.
	35-39	Respondent's current age is between 35 and 39 years.
	40-45	Respondent's current age is between 40 and 44 years.
	45+	Respondent's current age is above 45 years.
Educational attainment	Primary or below	No formal education
	Secondary or above	6–10 or more than 10 years of formal schooling
Media Exposure	No media exposure	Respondent does not get any exposure to mass media.
	At least once a week	Get exposure at least once a week from at least one media.
	Almost everyday	Get exposure almost every day from at least one media.
Disability	Yes	Respondent has at least one disability among difficulty seeing, hearing, walking, remembering or concentrating, self-care, and communicating.
	No	Respondent has no such disabilities.
**Household-level factors**		
Wealth Index	Bottom two	Household belongs to the bottom two wealth quantile.
	Upper three	Household belongs to the upper three wealth quantile.
Educational attainment of household head	Pre-primary or none	No formal education
	Primary	1–5 years of formal schooling
	Secondary or above	6–10 or more than 10 years of formal schooling
Sex of household head	Male	Sex of the household head is male.
	Female	Sex of the household head is female.
Religious affiliation	Muslim	Respondent belongs to a Muslim household.
	Non-Muslim	Respondent belongs to a non-Muslim household.
**Community-level factors**		
Community prevalence of sanitary napkin usage	Low prevalence	The community prevalence of sanitary napkins use is below 30%.
	Moderate prevalence	The community prevalence of sanitary napkins use is between 30 to 60%.
	High prevalence	The community prevalence of sanitary napkins use is higher than 60%.
Area of Residence	Rural	Respondent's type of place of residence is rural.
	Urban	Respondent's type of place of residence is urban.
Division	Dhaka	Respondent lives in the Dhaka division

Barisal	Respondent lives in the Barisal division
Chattogram	Respondent lives in the Chattogram division
Khulna	Respondent lives in the Khulna division
Mymensingh	Respondent lives in the Mymensingh division.
Rajhshahi	Respondent lives in the Rajshahi division
Rangpur	Respondent lives in the Rangpur division.
Sylhet	Respondent lives in the Sylhet division.

Statistical analyses

Univariate analysis was conducted to examine the sample characteristics. The chi-square (χ^2) test was used to assess associations between the outcome variable and explanatory variables. The outcome of interest, use of sanitary napkins, was then modeled using a generalized linear model (GLM) with a logit link function to estimate adjusted odds ratios (AORs) for the study factors. After fitting the model, the marginal probabilities for six groups, defined by combinations of wealth index and individual education level, were estimated using the margins command in Stata (Stata SE V.14, StataCorp).

~~Additionally, the spatial distribution of sanitary napkin usage across districts was illustrated using the R packages *sf* and *ggplot2* (V 4.3.2).~~ All analyses accounted for survey weights that were provided in the dataset~~,~~ to ensure representativeness. The spatial map was constructed using R (version 4.1.1). The district-level Shapefile of Bangladesh was accessed from the *bangladesh* package in R [33], which provides open-source geographic boundary data. Maps were generated using the *ggplot2* package (version 4.3.2) without the use of proprietary basemap services such as Google Maps, Google Earth, or Google Street View.

Formatted: Font: Italic

Formatted: Font: Italic

Results

Table 2 presents the demographic characteristics of 46,677 surveyed women aged 15-49 years. Notably, a predominant age group falls within the range of 20-39 years, representing 18.62% of the total sample. Educational attainment reveals a substantial majority, with 60.91% of respondents possessing secondary or higher qualifications. Regarding media exposure, a significant portion, accounting for 57.33% of the sample, reported being exposed to media almost daily. Approximately one-fifth (21.58%) of respondents acknowledged having a disability. Socioeconomic status demonstrates a notable disparity, with 35.41% of individuals falling within the bottom two wealth quantiles and 64.59% within the upper three. An examination of educational attainment within wealth indices reveals intriguing variations. Religious demographics indicate a

Muslim majority, comprising 90.24% of the sample, while male-headed households constitute 88.23%. Notably, 72.81% of the surveyed women reported

Table 2. Sample characteristics of the study participants.

	Distribution	
	**n (unweighted)**	**% (weighted)**
**Total**	46,677	100
**Age group**		
15-19	4,462	9.56
20-24	8,690	18.62
25-29	8,453	18.11
30-34	8,712	18.66
35-39	7,854	16.83
40-44	5,300	11.35
45-49	3,205	6.87
**Educational attainment**		
Pre-primary or none	7,209	15.44
Primary	11,039	23.65
Secondary or above	28,429	60.91
**Media exposure**		
Not at all	13,767	29.49
Less than once a week	2,233	4.78
At least once a week	3,914	8.39
Almost every day	26,762	57.33
**Disability**		
Not disable	36,593	78.4
Disable	10,075	21.58
**Wealth Index**		
Bottom two	16,530	35.41
Upper three	30,147	64.59
**Educational attainment**		
Pre-primary or none	14,503	31.07
Primary	12,728	27.27
Secondary or above	19,445	41.66
**WI & Educational attainment**		
Pre-primary or none & Bottom two	4,278	9.17
Pre-primary or none & Upper Three	2,931	6.28
Primary & Bottom two	5,444	11.66
Primary & Upper Three	5,595	11.99
Secondary & Bottom two	6,808	14.59

	Distribution	
	**n (unweighted)**	**% (weighted)**
Secondary & Upper three	21,621	46.32
**Religious affiliation**		
Other Religion	4,556	9.76
Muslim	42,121	90.24
**Sex of household head**		
Female	5,492	11.77
Male	41,184	88.23
**Community prevalence of sanitary napkin useage**		
Low prevalence	33,821	72.81
Moderate prevalence	10,188	21.93
High prevalence	2,444	5.26
**Area of residence**		
Rural	35,245	75.51
Urban	11,432	24.49
**Division**		
Barisal	2,482	5.32
Chattogram	8,835	18.93
Dhaka	12,114	25.95
Khulna	5,526	11.84
Mymensingh	3,062	6.56
Rajshahi	6,262	13.42
Rangpur	4,966	10.64
Sylhet	3,429	7.35

using sanitary napkins, with the majority residing in rural areas (75.51%). Geographically, Dhaka emerges as the most represented division (25.95%), followed by Chattogram (18.93%).

The distribution of the proportion of women using sanitary napkins across districts in Bangladesh is illustrated in Figure 1. The spatial distribution reveals a generally poor level of sanitary napkin use throughout the country. In only five out of 64 districts does nearly one in three women use sanitary napkins, —still a very low proportion. The northern region shows the lowest usage rates, with Lalmonirhat having the lowest proportion, where approximately 6% of women use sanitary

napkin.

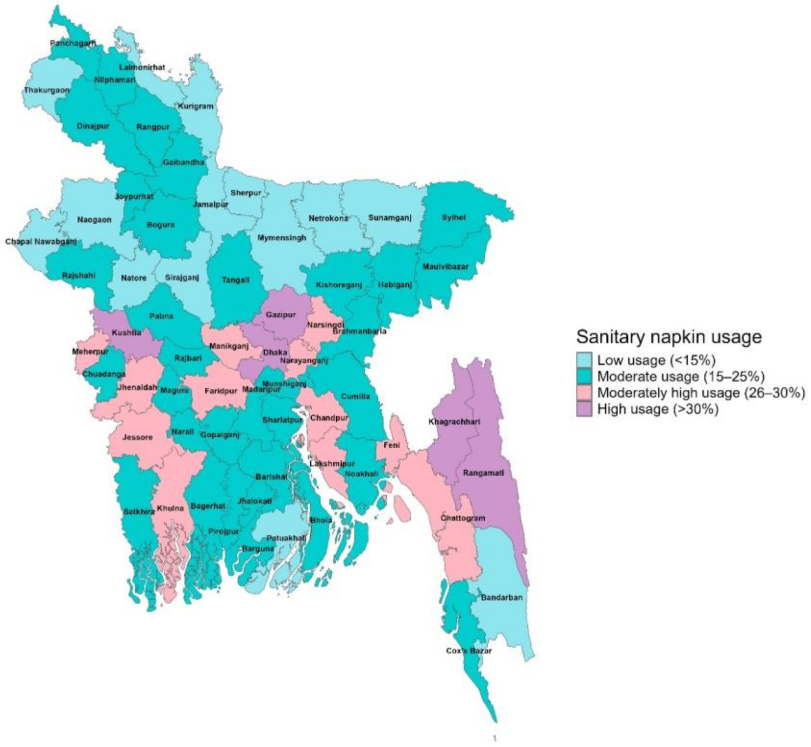

Fig 1. District-wise mapping on the distribution of sanitary napkin useage in Bangladesh. (Source of administrative boundaries: *bangladesh* R package (open-source shapefile data). Source of estimates: analysis from BMICS data 2019.)

Formatted: Font: 12 pt, Not Italic

Formatted: Font: 12 pt, Not Italic

Formatted: Font: 12 pt

Formatted: Font: 12 pt, Not Italic

Formatted: Font: 12 pt, Not Italic

Formatted: Justified, Keep with next

Sanitary napkin useage by education and wealth

The overall prevalence of sanitary napkin usage among women aged 15–49 years was 23.4% (Supplementary table 1). Women with secondary or higher education from the bottom two wealth quintiles had a higher prevalence of sanitary napkin use (11.84%) than women from the upper

three wealth quintiles who had pre-primary or no education (5.09%) or only primary education (10.82%) (Figure 2).

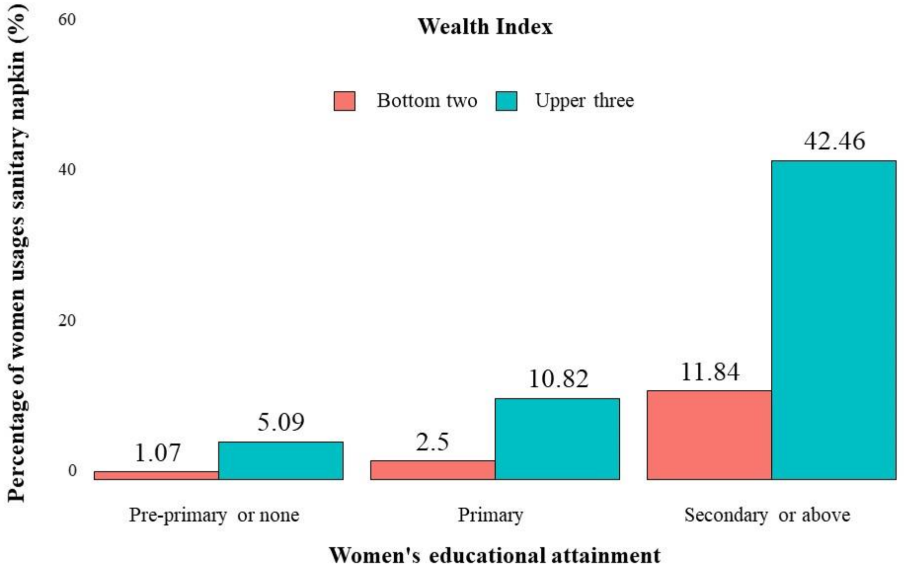
Wealth Index
Bottom two
Upper three
Percentage of women usages sanitary napkin (%)
60
40
20
0
1.07
5.09
2.5
10.82
11.84
42.46
Pre-primary or none
Primary
Secondary or above
Women's educational attainment

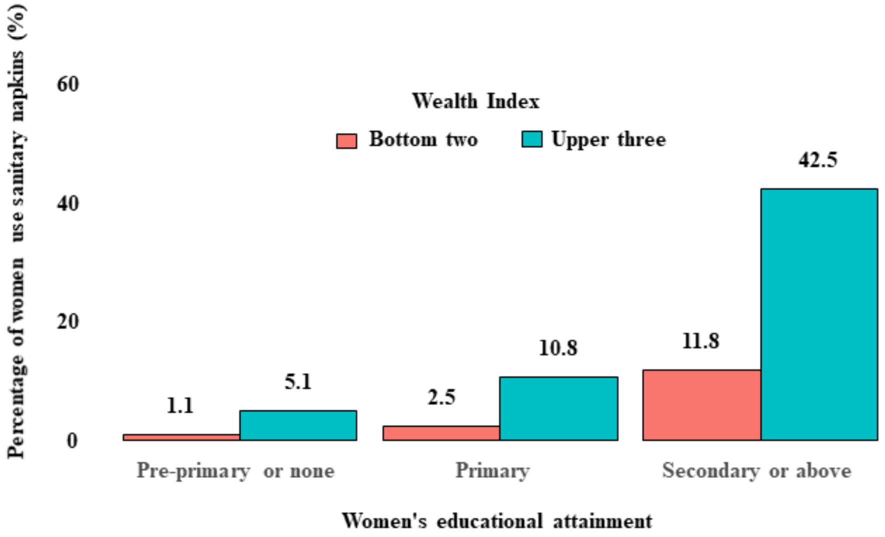
Wealth Index
Bottom two
Upper three
Percentage of women use sanitary napkins (%)
60
40
20
0
1.1
5.1
2.5
10.8
11.8
42.5
Pre-primary or none
Primary
Secondary or above
Women's educational attainment

Fig 2. Percentage of women usage sanitary napkins over educational attainment and wealth index

Formatted: Font: (Default) Times New Roman, 12 pt

Formatted: Normal, Justified

Formatted: Font: (Default) Times New Roman, 12 pt

Table 3 presents adjusted odds ratios (AOR) with associated p-values and 95% confidence intervals (CI) for the relationship between the type of material used during menstruation and various demographic factors. Notable findings include a higher likelihood of using sanitary napkins in younger age groups, with substantial increases in odds ratios from 25-29 years (AOR=2.57) to 15-19 years (AOR=6.09). Education plays a significant role, with increasing AORs for primary (AOR=1.44) and Secondary or above (AOR=4.5) compared to pre-primary or none. Media exposure shows a positive association, with higher odds for more frequent exposure. Individuals without disabilities have higher odds (AOR=0.86) of using Sanitary Napkins. The wealth index indicates significantly higher odds (AOR=2.3) in the upper three quantiles. Notable variations exist in the odds of sanitary napkin usage across different divisions and areas of residence, with urban residents, Dhaka division, and areas with high community prevalence having higher odds.

Table 3. Association of women's education and wealth with sanitary napkin usage

	**AOR**	**p-value**	**95% CI**	
			Lower	Upper
**Educational attainment**				
Pre-primary or none	1 (Ref.)			
Primary	1.44	0.048	1.0	2.05
Secondary or above	4.50	<0.01	3.28	6.16
**Wealth index**				
Bottom two	1 (Ref.)			
Upper three	2.30	<0.01	1.58	3.34
**Education & WI**				
Primary & Upper three	1.03	0.90	0.66	1.59
Secondary or above & Upper three	1.33	0.144	0.91	1.95
**Age**				
45-49	1 (Ref.)			
40-44	1.08	0.389	0.90	1.29
35-39	1.34	<0.01	1.14	1.58
30-34	1.54	<0.01	1.30	1.83
25-29	2.57	<0.01	2.18	3.03
20-24	4.17	<0.01	3.54	4.92
15-19	6.09	<0.01	5.13	7.23
**Media exposure**				
Not at all	1 (Ref.)			
Less than once a week	1.27	<0.01	1.08	1.51
At least once a week	1.42	<0.01	1.25	1.62
Almost every day	1.93	<0.01	1.76	2.11
**Disability**				
Not disable	1 (Ref.)			

	AOR	p-value	95% CI	
			Lower	Upper
Disable	0.86	<0.01	0.80	0.93
**Educational attainment of household head~~HH~~**				
P~~p~~re-primary or none	1 (Ref.)			
P~~p~~rimary	1.12	0.03	1.01	1.23
S~~s~~econdary or above	2.11	<0.01	1.92	2.30
**Religious affiliation**				
Muslim	1 (Ref.)			
Other Religion	1.04	0.454	0.93	1.17
**Sex of Household head**				
Female	1 (Ref.)			
Male	0.84	<0.01	0.76	0.93
**Community prevalence of sanitary napkin usage**				
Low Use	1 (Ref.)			
Moderate Use	1.80	<0.01	1.59	2.03
High Use	3.75	<0.01	3.06	4.59
**Area of residence**				
Rural	1 (Ref.)			
Urban	1.50	<0.01	1.38	1.64
**Division**				
Dhaka	1 (Ref.)			
Barishal	1.15	0.02	1.02	1.29
Chattogram	0.74	<0.01	0.68	0.80
Khulna	0.92	0.06	0.84	1.01
Mymensingh	0.60	<0.01	0.50	0.71
Rajshahi	0.68	<0.01	0.62	0.76
Rangpur	0.67	<0.01	0.60	0.75
Sylhet	0.85	0.051	0.73	1.00
**Intercept**	0.01	<0.01	0.00	0.01

From the fitted regression model, we have calculated the marginal probabilities of sanitary napkin usage for every subgroup created by two variables: educational attainment and wealth quintiles. The national estimates from figure 3 shows that, educational attainment significantly increases the likelihood of using sanitary napking among women aged 15-49 years in Bangladesh. Women with secondary or higher educational attainment from~~ a~~ poorer households (bottom two quintiles) had a higher chance of using~~e~~ sanitary napkin compared to women with pre-primary or no educational attainment from a rich household (upper three). This behavibiour was consistent across all ~~-~~divisions.

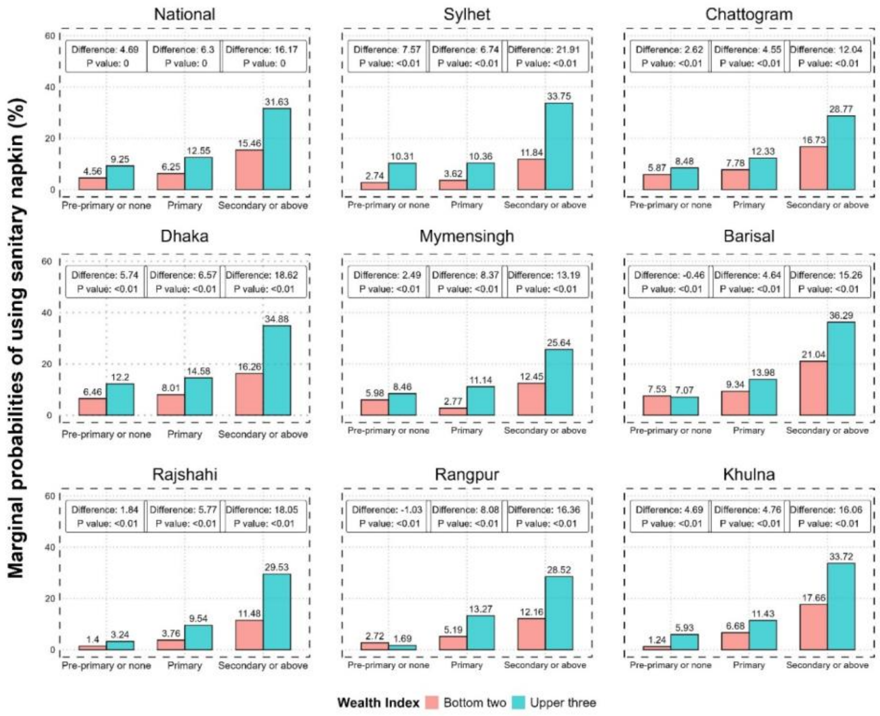

p-value from t-test

Fig 3. Marginal probabilities of sanitary napkin usage by women's education and wealth.

Discussion

This study highlights the significant roles of both awareness and affordability in determining sanitary napkin use among women in Bangladesh. The results suggest that awareness has a particularly strong association with sanitary napkin use compared to affordability. Besides less educated, younger, and disabled women, those living in male-headed or less educated households, with limited media exposure, residing in rural areas or in divisions like Mymensingh and Rangpur, and living in communities with low sanitary napkin use were less likely to use sanitary napkins.

Factors influencing sanitary napkin use~~Comparison with earlier studies~~

Consistent with prior research, this study identified significant variation in sanitary napkin use across geographic, household, and individual levels [34]. Findings indicates there are ~~The study~~

Formatted: Font: Bold

Formatted: Normal (Web)

Formatted: Font color: Auto

Formatted: Font: Bold

Formatted: Normal (Web), Line spacing: single

~~observed~~ significant differences in menstrual hygiene practices across divisions, urban and rural areas, which are in line with previous studies [17]. This difference is might be due to some unobserved factors, which vary across regions.

At ~~Another areal-level factor influencing sanitary napkin uses is~~ the community-level, ~~-~~prevalence of sanitary napkin use within a community was a significant predictor of individual use, reflecting the role of descriptive norms in shaping health behavior which consistent with Social Cognitive Theory [35]. ~~, which reflects shared norms, awareness, and accessibility.~~ In Bangladesh, where ~~cultural stigmas around~~ menstruation remain culturally stigmatized, ~~strong, women and girls in~~ communities with higher sanitary napkin use ~~are more~~ likely foster greater openness and awareness, reducing the social cost of adoption.~~to adopt proper hygiene practices. This is because such communities often foster greater awareness and openness. In contrast, lower-prevalence areas tend to be more restrictive and less informed.~~ Similar patterns have been observed elsewhere: in India, Sood et al. found that positive social norms significantly improved MHM [36]; in Nepal, Shrestha et al. reported that caste-based taboos limited awareness regarding MHM [37].

At the ~~Apart from area-level factors,~~ household-level, w~~-factors also significantly influence the menstrual practices of women and adolescent girls. W~~omen in male-headed households are less likely to use sanitary napkins, consistent with evidence that household gender dynamics and educational level of the household head influence women's health knowledge, autonomy, and access to resources ~~have adequate knowledge of menstrual hygiene management compared to those in female-headed or households with an educated head~~ [26]. From the perspective of the Andersen Behavioral Model [38], this finding indicates that household social structure may influence women's access to and use of sanitary napkins,.

Formatted: Font: Bold

At the ~~which, in turn, negatively affect menstrual hygiene practices.~~

~~Lastly,~~ individual-level ~~factors have a significant role in explaining the variation of sanitary napkin uses. M~~mass media exposure seen to have a significant role in menstrual hygiene practices which serves as a core source of information on MHM [30]. Women who are exposed to media more frequently are more likely to have better knowledge and use of sanitary products, with those accessing media almost every day showing the highest likelihood. The intersection of menstruation and disability endangers an intricate set of circumstances that can give rise to compounded discrimination and present distinct challenges for menstruating individuals with disabilities [24]. This study also found evidence supporting this phenomenon.

Interplay between awareness and affordability in napkin use

Our findings reveal that both awareness and affordability significantly influence the use of sanitary napkins, underscoring their interdependent roles in ensuring effective MHM. While higher levels of awareness are strongly associated with increased use, this effect is amplified in the presence of affordability, indicating a synergistic relationship between the two factors.

Awareness enhances knowledge of menstruation as a biological process, reduces stigma, and improves attitudes towards the use of hygienic products [39]. Due to lack of awareness, menstrual hygiene is often deprioritized and menstrual products are overlooked in basic WASH and

sanitation planning [40]. However, even with increased awareness and affordability~~Regardless of affordability~~, many girls still refrain from using them due to embarrassment and stigma. A cross-sectional survey of adolescent girls in Rajshahi revealed that, factors like embarrassment purchasing pads from male shopkeepers and stigma surrounding menstruation were major barriers to sanitary napkin use [41]. This suggests that socio-cultural taboos pose further barriers to access that persist even when informational and economic constraints are addressed.

However, knowledge alone does not always translate into practice when economic barriers remain. This is particularly relevant in low-income households where affordability and availability is the key barriers [42]. In such settings, even when girls are aware of the benefits of sanitary napkins, they may be forced to rely on unsafe alternatives due to cost constraints.

Action taken to promote sanitary napkins

In Bangladesh, the government recognizes MHM as essential for women's and girls' health and has prioritized awareness and affordability in the National MHM Strategy 2021 [43]. To reduce costs and promote local manufacturing, the government has waived VAT on raw materials for sanitary napkin production. Additionally, the National MHM Coordination Committee has been established to oversee and align MHM efforts across health, education, and social sectors.

Despite these efforts, a 15% VAT on finished sanitary napkin products remains a significant barrier to affordability, which was also reflected by our findings that women from poorer households were less likely to use sanitary napkins. To address this, the strategy calls for revisiting the VAT policy to make napkins more affordable. It also emphasizes improving distribution networks to ensure access in workplaces, schools, healthcare centers, and public spaces, alongside expanding MHM awareness sessions to marginalized and hard-to-reach communities.

Notably, the current NCTB curriculum (after the recent update in 2025) does not adequately address menstruation and menstrual hygiene management, particularly in the subject "Physical Education and Health" for classes 6, 7, and 8[44-46]. These textbooks lack essential information on menstruation, the use of sanitary napkins, and guidance on changing intervals, which are critical for maintaining good menstrual hygiene practices. As a result, many adolescent girls remain uninformed or misinformed about safe and hygienic practices during menstruation. Revising the curriculum to include these crucial topics is imperative for equipping adolescents with the accurate knowledge and practical skills they need to manage menstruation.

On the other hand, non-governmental research organizations have implemented programs to increase awareness about menstruation. One such initiative was the Ritu Programme, funded by the Embassy of the Kingdom of the Netherlands in Bangladesh and implemented from November 2015 to March 2020. The programme aimed to improve menstrual health among girls aged 10–15 in Netrokona, Bangladesh[47]. Through evidence-informed interventions, ~~—~~including school-based menstrual health education, community engagement with parents, and a national-level mass media campaign, ~~—~~the programme significantly boosted menstruation awareness and management. It notably increased girls' confidence and understanding of menstruation, improved hygiene practices (like changing materials more frequently and increased sanitary pad use).

Taken together, the findings underscore that MHM is shaped by a complex interplay of structural, social, and individual determinants. While individual awareness and household affordability are critical, they do not operate in isolation; rather, they are embedded within broader contextual factors such as community norms, media exposure, educational content, and policy frameworks. Therefore, effective menstrual health interventions must adopt a multi-level approach that simultaneously targets these interconnected domains.

Strengths and Limitations of the study

A key strength of this study is the use of nationally representative MICS 2019 data, which ensures broad generalizability. It applies a generalized linear model to estimate adjusted associations and calculates marginal probabilities to clearly quantify the combined effects of ~~education~~ awareness and ~~wealth~~ affordability on sanitary napkin use. By directly comparing subgroups (e.g., educated-poor vs. uneducated-rich), the study provides concrete evidence on the relative influence of awareness and affordability, offering actionable insights for targeted MHM interventions.

This study has several limitations. First, the cross-sectional nature of the data limits the scope of estimating the causal effects. Second, although we adjusted for major socio-demographic covariates, the potential for residual confounding remains from unmeasured factors such as cultural beliefs, familial traditions, or regional product availability. ~~Second~~Third, awareness and affordability were measured using proxy indicators, ~~—~~educational attainment and household wealth index, ~~—~~which may not fully capture the complexity of these constructs. Specifically, formal education serves as a proxy for health literacy but does not directly measure specific knowledge about menstrual physiology or hygienic practices. Similarly, the household wealth index reflects overall socio-economic status and asset ownership rather than a woman's individual financial autonomy or her direct purchasing power to buy sanitary products. ~~Third~~Forth, self-reported data on menstrual hygiene practices may be subject to social desirability or recall bias. Lastly, certain contextual factors such as cultural norms, school-based education on menstruation, and access to WASH (Water, Sanitation, and Hygiene) facilities were not available in the dataset and could not be accounted for in the analysis.

Another limitation is that the survey included only women aged 15–49 years, excluding younger adolescents who also menstruate and may have different hygiene practices.

Conclusion

This study demonstrates that menstrual hygiene management is influenced by interconnected factors at the individual, household, and community levels. Awareness and affordability show significant interaction effects, with awareness emerging as a key driver of sanitary napkin use across wealth quintiles. Therefore, standalone economic or informational campaigns may be incomplete. To be actionable, policymakers should implement integrated menstrual hygiene management interventions, such as incorporating comprehensive reproductive health literacy into the national school curriculum alongside targeted subsidies or tax exemptions on raw materials for

sanitary products to lower market prices. These strategies are vital for reducing sub-national disparities and ensuring sustainable, hygienic menstrual practices across diverse socio-economic contexts in Bangladesh

However, awareness alone cannot overcome the barriers posed by limited economic resources.

To ensure equitable and effective menstrual hygiene management, a dual approach is essential—one that combines widespread menstrual health education with access to affordable sanitary products. Only by addressing both informational and economic barriers can we promote safe, dignified, and sustainable menstrual practices for all women and girls in Bangladesh.

Declarations

Ethics approval and consent to participate

Not applicable. The BMICS 2019 provides de-identified data, which cannot be used to identify any study participants. Regarding the ethics approval, the BMICS 2019 protocol was approved by the technical committee of the Government of Bangladesh, led by the Bangladesh Bureau of Statistics (BBS). As the study utilized publicly BMICS 2019 as a secondary data source,available and anonymized secondary data, it does not require additional ethical approval, nor does it have any information to identify study participants.

Consent for publication

Not applicable.

Availability of data and materials

The datasets analyzed during the current study are available on the MICS program website, https://microdata.worldbank.org/index.php/catalog/MICS/

Competing interests

None declared.

Funding

The authors received no specific grant funding for this research.

Authors' contributions

MHP, MTA, and MMR conceptualized the study. MHP and MMR performed data curation and conducted the formal statistical analysis. MHP, MTA, SNE, and MMR contributed to the writing of the original draft. MMR supervised the study. SNE contributed to the literature review. All authors reviewed, edited, and provided critical feedback on the manuscript. All authors read and approved the final version of the manuscript.

Acknowledgments

The authors would like to thank UNICEF and the Bangladesh Bureau of Statistics (BBS) for providing access to the 2019 Bangladesh Multiple Indicator Cluster Survey (MICS) dataset. icddr,b acknowledges the Government of the People's Republic of Bangladesh and Canada for providing core/unrestricted support.

References

Formatted: Justified

1. Habeeb, S., *Menstruation*, in *Encyclopedia of Sexual Psychology and Behavior*, T.K. Shackelford, Editor. 2023, Springer International Publishing: Cham. p. 1–11.
2. Critchley, H.O.D., et al., *Physiology of the Endometrium and Regulation of Menstruation.* Physiol Rev, 2020. **100**(3): p. 1149–1179.
3. Likis, K.D.S.a.F.E., *Women's Gynecologic Health*. Vol. Second Edition. 2013.
4. Dutta, D., et al., *Supporting the rights of girls and women through Menstrual Hygiene Management (MHM) in the East Asia and Pacific Region: Realities, progress and opportunities.* Bangkok, Thailand: UNICEF East Asia and Pacific Regional Office (EAPRO), 2016.
5. Sommer, M. and M. Sahin, *Overcoming the taboo: advancing the global agenda for menstrual hygiene management for schoolgirls.* American journal of public health, 2013. **103**(9): p. 1556–1559.
6. Korir, E., F.N. Okwara, and G. Okumbe, *Menstrual hygiene management practices among primary school girls from a pastoralist community in Kenya: a cross sectional survey.* Pan African Medical Journal, 2018. **31**(1).
7. Olson, M.M., et al., *The persistent power of stigma: A critical review of policy initiatives to break the menstrual silence and advance menstrual literacy.* PLOS Global Public Health, 2022. **2**(7): p. e0000070.
8. Maulingin-Gumbaketi, E., et al., *Socio-cultural implications for women's menstrual health in the Pacific Island Countries and Territories (PICTs): a scoping review.* Reproductive health, 2022. **19**(1): p. 128.
9. UNICEF, *Menstrual hygiene | UNICEF. Avilable: https://www.unicef.org/wash/menstrual-hygiene.* 2023.
10. UNICEF. *Menstrual hygiene: Gender inequality, cultural taboos and poverty can cause menstrual health needs to go unmet.* . Available from: https://www.unicef.org/wash/menstrual-hygiene.
11. Ali, T.S., N. Sami, and A.K. Khuwaja, *Are unhygienic practices during the menstrual, partum and postpartum periods risk factors for secondary infertility?* J Health Popul Nutr, 2007. **25**(2): p. 189–94.
12. Anand, E., J. Singh, and S. Unisa, *Menstrual hygiene practices and its association with reproductive tract infections and abnormal vaginal discharge among women in India.* Sex Reprod Healthc, 2015. **6**(4): p. 249–54.
13. Das, P., et al., *Menstrual Hygiene Practices, WASH Access and the Risk of Urogenital Infection in Women from Odisha, India.* PLoS One, 2015. **10**(6): p. e0130777.
14. Torondel, B., et al., *Association between unhygienic menstrual management practices and prevalence of lower reproductive tract infections: a hospital-based cross-sectional study in Odisha, India.* BMC Infect Dis, 2018. **18**(1): p. 473.
15. Hennegan, J. and P. Montgomery, *Do Menstrual Hygiene Management Interventions Improve Education and Psychosocial Outcomes for Women and Girls in Low and Middle Income Countries? A Systematic Review.* PLoS One, 2016. **11**(2): p. e0146985.
16. Krenz, A. and H. Strulik, *The impact of menstruation hygiene management on work absenteeism of women in Burkina Faso.* Econ Hum Biol, 2021. **43**: p. 101067.

17. Tegegne, T.K. and M.M. Sisay, *Menstrual hygiene management and school absenteeism among female adolescent students in Northeast Ethiopia.* BMC Public Health, 2014. **14**: p. 1118.
18. Sommer, M., et al., *A Time for Global Action: Addressing Girls' Menstrual Hygiene Management Needs in Schools.* PLoS Med, 2016. **13**(2): p. e1001962.
19. Sommer, M., et al., *How addressing menstrual health and hygiene may enable progress across the Sustainable Development Goals.* Glob Health Action, 2021. **14**(1): p. 1920315.
20. Hennegan, J. and L. Sol, *Confidence to manage menstruation at home and at school: findings from a cross-sectional survey of schoolgirls in rural Bangladesh.* Culture, health & sexuality, 2020. **22**(2): p. 146–165.
21. Ahmed, M.S., et al., *Association between menstrual hygiene management and school performance among the school-going girls in rural Bangladesh.* Adolescents, 2021. **1**(3): p. 335–347.
22. Hasan, M., et al., *Menstrual hygiene practices and school absenteeism among adolescent girls in Bangladesh: A cross-sectional study. Popul Med 2021; 3: 8*. 2021.
23. Khan, S., et al., *Taking measures before opportunities bleed away: Current knowledge and policy gaps in menstrual hygiene management among adolescent girls in Bangladesh.* United States Agency for International Development (USAID), 2022.
24. A. Lacroix, H.G., K. Shumway, and M. Langaker. *Physiology, Starting*. Available from: https://gifsided.com/female-reproductive-system-questions-and-answers-pdf.
25. Sibli, M.S., et al., *Analysis of the average menopause age of Bangladeshi women: A public health perspective.* World Journal of Advanced Research and Reviews, 2022. **16**(3): p. 231–235.
26. Afiaz, A. and R.K. Biswas, *Awareness on menstrual hygiene management in Bangladesh and the possibilities of media interventions: using a nationwide cross-sectional survey.* BMJ Open, 2021. **11**(4): p. e042134.
27. Sumpter, C. and B. Torondel, *A systematic review of the health and social effects of menstrual hygiene management.* PLoS One, 2013. **8**(4): p. e62004.
28. Mondal, B.K., et al., *Practices and effects of menstrual hygiene management in rural Bangladesh.* 2017.
29. Jejeebhoy, S.J., *Women's education, autonomy, and reproductive behaviour: Experience from developing countries*. 1995: Oxford University Press.
30. Garg, S., et al., *Menstrual hygiene management and its determinants among adolescent girls in low-income urban areas of Delhi, India: a community-based study.* Osong Public Health Res Perspect, 2022. **13**(4): p. 273–281.
31. Wasan, Y., et al., *Practices and predictors of menstrual hygiene management material use among adolescent and young women in rural Pakistan: A cross-sectional assessment.* J Glob Health, 2022. **12**: p. 04059.
32. Bhusal, C.K., *Practice of Menstrual Hygiene and Associated Factors among Adolescent School Girls in Dang District, Nepal.* Adv Prev Med, 2020. **2020**: p. 1292070.
33. *Mahmud, J. (2022). bangladesh: Provides Easy Access to 'Bangladesh' Demographic and Health Survey Data (Version 1.0.0) [Computer software]. https://CRAN.R-project.org/package=bangladesh.*
34. Chauhan, S., et al., *Examining the predictors of use of sanitary napkins among adolescent girls: A multi-level approach.* PLOS ONE, 2021. **16**(4): p. e0250788.

35. Zhai, S., et al., *Analysis, evaluation, and reformulation of social cognitive theory: Toward parent-child shared management in sleep health.* J Pediatr Nurs, 2023. **73**: p. e65–e74.
36. Sood, S., et al., *Measuring the effectiveness of communication programming on menstrual health and hygiene management (MHM) social norms among adolescent girls in India.* Glob Public Health, 2021. **16**(4): p. 578–589.
37. Khanal, G., et al., *Menstruation hygiene management among secondary school students of Chitwan, Nepal:a cross-sectional study.* BMC Women's Health, 2023. **23**(1): p. 395.
38. Alkhawaldeh, A., et al., *Application and Use of Andersen's Behavioral Model as Theoretical Framework: A Systematic Literature Review from 2012-2021.* Iran J Public Health, 2023. **52**(7): p. 1346–1354.
39. Sommer, M., et al., *Comfortably, Safely, and Without Shame: Defining Menstrual Hygiene Management as a Public Health Issue.* Am J Public Health, 2015. **105**(7): p. 1302–11.
40. Mahon, T. and M. Fernandes, *Menstrual hygiene in South Asia: a neglected issue for WASH (water, sanitation and hygiene) programmes.* Gender & Development, 2010. **18**(1): p. 99–113.
41. Ha, M.A.T. and M.Z. Alam, *Menstrual hygiene management practice among adolescent girls: an urban-rural comparative study in Rajshahi division, Bangladesh.* BMC Womens Health, 2022. **22**(1): p. 86.
42. Dasgupta, A. and M. Sarkar, *Menstrual Hygiene: How Hygienic is the Adolescent Girl?* Indian J Community Med, 2008. **33**(2): p. 77–80.
43. *Government of Bangladesh. (2021). National Menstrual Hygiene Management (MHM) Strategy 2021. Ministry of Women and Children Affairs. Available at: https://psb.gov.bd/policies/nmhmsen.pdf.*
44. *National Curriculum and Textbook Board (NCTB). (2024). Physical Education & Health, Class 6. Dhaka: National Curriculum and Textbook Board.*
45. *National Curriculum and Textbook Board (NCTB). (2024). Physical Education & Health, Class 7. Dhaka: National Curriculum and Textbook Board.*
46. *National Curriculum and Textbook Board (NCTB). (2024). Physical Education & Health, Class 8. Dhaka: National Curriculum and Textbook Board.*
47. *Simavi, RedOrange, TNO, BNPS, & DORP. (2020). Ritu programme – Improving menstrual health of girls in Bangladesh: Final report 2016-2020.*

Formatted: Justified, Line spacing: Multiple 1.15 li

Awareness or Affordability: What matters the most in ensuring menstrual hygiene management?

Rebuttal Letter

Reviewer 03.

Comment 1 (*line 17: awareness regarding menstrual hygiene*)**:** This will require a more detailed definition in the paper -- awareness of menstrual hygiene from a health/biological standpoint? Management of menstrual hygiene/product use? Options for products? What do we mean by awareness here?

Response 1: We have now clear out what do we meant by awareness here (knowledge regarding the menstrual hygiene management) and also broaden the explanation in the explanatory variables part (lines 131).

Comment 2 (*line 23: individual educational attainment*)**:** I'd be curious to see more justifications around the selection of this proxy -- e.g. some sort of evidence/justification around school/educational facilities being the critical entry point to awareness around menstrual products.

Response 2: We thank the reviewer for this important comment. We have justified the use of this proxy in the explanatory variable part (lines 131-134).

Comment 3 (*line 31: sanitary napkins*)**:** Were only sanitary napkins assessed? Or was this meant to be inclusive of the full range of menstrual products?

Response 3: The term “sanitary napkins” refers to reported use of sanitary pads during the last menstrual period, rather than assessment of a full range of individual product types separately (please see the updated outcome variable section; lines 122-125).

Comment 4 (*line 58: This poor management of menstrual hygiene*)**:** Suggest re-framing to a way that doesn't place blame on the person who menstruates -- there has largely been a shift away from framing around 'menstrual hygiene' and towards 'menstrual health' for this reason. In this framing, it sounds like the person who menstruates is to blame for these health outcomes, rather than the system that has failed them.

Response 4: We thank the reviewer for this important suggestion. We have revised the text accordingly (lines 56-57).

Comment 5 (*line 81-82: only a quarter of adolescent girls know about menstruation before their menarche*)**:** This seems to suggest that menstrual health is not taught in schools, which feels counter to the proxy indicator of educational status to indicate awareness about menstrual health/menstrual products.

Response 5: We thank the reviewer for this sharp observation. We agree that the low rate of pre-menarche knowledge seems contradictory at first glance. This statistic reflects a well-documented reality in Bangladesh: reproductive health topics are often introduced too late in the curriculum (or skipped altogether in primary school), leaving young girls uninformed at the actual onset of menarche. However, our proxy of "educational attainment" for women aged 15–49 measures their cumulative exposure to schooling. As women progress into secondary and higher education, they gain delayed but crucial exposure to health curricula. Thus, while early school fails to prepare them before menarche, longer retention in the education system is still the primary driver of MHM awareness.

Comment 6 (*line 117: The survey collected detailed information from women of reproductive age (15–49 years)*)**:** Suggest acknowledging the gap on young adolescent data -- the average age of menarche is much younger than 15, which means that we aren't capturing the full menstruating population with these data. This is hard to address since MICS / survey data don't include this, but suggest highlighting this potential gap.

Response 6: We have added this as a limitation (lines 325-327).

Comment 7 (*line 125-127: In this study, sanitary napkin use was considered a proxy for proper menstrual hygiene, as they are typically cleaner, safer, and more absorbent than traditional or improvised materials*)**:** Better define what is meant by sanitary napkin and whether this includes both reusable and disposable options or just disposable/single use. Include context on whether cloth/non-purpose made materials were considered to 'count' as sanitary napkins in this definition.

Response 7: We thank the reviewer for this comment. Sanitary napkin use is defined as any reported use of purpose-made sanitary pads during the last menstrual period, regardless of exclusive or combined use (refer to the updated outcome variable section).

Comment 8 (*line 128*)**:** An additional variable that should be part of this analysis is actual availability of products -- availability itself is often a barrier and if sanitary napkins are not readily available in certain communities (most often, rural or vulnerable communities, areas with lower household wealth, etc), this creates a confounding variable that influences outcome. Suggest adding analysis on this that speaks to how readily available products are in these regions... beyond just actual use of products. Use of products and prevalence of community use still rely on products being available to purchase and use.

Response 8: We thank the reviewer for this point. Unfortunately, the 2019 Bangladesh MICS dataset does not contain variables on commercial market supply or physical product availability in communities.

Comment 9 (*line 135-137: Individual level factors that can affect use of sanitary napkin included participants age, educational attainment, exposure to media, and status of disability*)**:** Suggest acknowledging that you are missing a critical age group within these data, since the data begin at age 15 but average age of menarche is much lower. I would acknowledge this and contextualize a bit further.

Response 9: We have added this as a limitation (lines 325-327).

Comment 10 (*Table 1: The community prevalence of sanitary napkins use is below 30%*)**:** Contextualize what this includes. Can you also clarify how prevalence is defined here? Is it a simple yes/no on access to sanitary pads, or does it measure for *sufficient* access, e.g. sufficient product access for the full cycle, rather than supplementation with non-purpose made products (e.g. 1-4 sanitary pads per cycle, supplemented with cloth or other materials).

Response 10: We thank the reviewer for this comment. The community prevalence was built on to the binary outcome defined in the outcome variable section, where sanitary napkin use was measured as any reported use during the last menstrual period (yes/no), regardless of quantity or whether other materials were also used (lines 144-146).

Comment 11 (*lines 192-194: Notable findings include a higher likelihood of using sanitary napkins in younger age groups, with substantial increases in odds ratios from 25-29 years (AOR=2.57) to 15-19 years (AOR=6.09)*)**:** This seems counter to the findings that higher educational levels result in higher likelihood of use, as most women/girls with higher education are likely to be older than those with lower.

Response 11: We thank the reviewer for this important comment. The findings reflect two different effects estimated in the adjusted model. Age captures cohort differences in menstrual hygiene practices, where younger women are more likely to adopt sanitary napkins due to more recent exposure to menstrual health information and changing norms. Education, on the other hand, reflects individual awareness and knowledge, which increases the likelihood of sanitary napkin use across all age groups. Therefore, higher use among younger women and more educated women are not contradictory, but represent separate pathways influencing behavior.

Comment 12 (*lines 206-207: educational attainment significantly increases the likelihood of using sanitary napking among women aged 15-49 years in Bangladesh*)**:** I think that this is a more powerful finding than framing the findings around educational attainment as being a proxy for 'awareness.'

Response 12: We agree with the reviewer that formal educational attainment is a powerful determinant with broader societal implications beyond acting as a proxy for awareness. However, to keep the paper tightly aligned with our research question, our analysis and discussion focus specifically on its role in health awareness and information access.

Comment 13 (*lines 216-217: This study highlights the significant roles of both awareness and affordability in determining sanitary napkin use among women in Bangladesh*)**:** For this takeaway to be highlighted, I think that much more work is needed to do justify educational attainment as a proxy for awareness. I think that the stronger framing is that educational attainment is significant in determining sanitary napkin use -- and a potential policy takeaway/discussion lens could be unpacking how education might lead to increased awareness. I also think that we need much more contextual definition on what is meant by awareness here.

Response 13: We thank the reviewer for this observation. As detailed in our Explanatory Variables (lines 131-138) and Discussion sections (lines 256-259), we use formal education as an established proxy for health awareness because higher educational attainment is a primary structural pathway through which women access health literacy and information on menstrual hygiene.

Comment 14 (lines 225: Comparison with earlier studies)**:** Is this the right header here? This seems to be looking at other individual factors within the context of this study, rather than prior studies. Additional studies are woven in to this discussion, but suggest re-framing the header to focus more on the other individual factors as the focus of this section.

Response 14: We thank the reviewer for this helpful suggestion. We have revised the header to “Factors influencing sanitary napkin use”.

Comment 15 (lines 232: proper hygiene practices.)**:** Avoid framing like 'proper' hygiene practices -- this shifts blame to the person menstruating rather than a system that has failed her.

Response 15: We thank the reviewer for this insightful comment. We agree that the term "proper" can inadvertently imply individual blame. We have revised the manuscript to replace "proper" with "recommended" to focus on the practices themselves rather than casting judgment on the individual.

Comment 16 (lines 255-258)**:** I think that this context needs to come much earlier in the paper to help readers understand what is meant by awareness -- and why education might be a proxy for it.

Response 16: We thank the reviewer for the suggestion. In the revised manuscript, we have broadened the rationale for using these proxies (lines 131-138).

Comment 17 (lines 258-261)**:** This next part seems to focus more on stigma/shame/taboo than on awareness. I would suggest either (1) expanding on why/how awareness reduces stigma/shame and therefore overcomes this; or (2) saying that even with increased awareness, stigma/shame pose further barriers to access.

Response 17: We thank the reviewer for this helpful suggestion. We have revised the Discussion (according to option 2) to clarify that (lines 262-264).

Comment 18 (lines 263: availability)**:** Did your statistical analysis take this into account at all? E.g. was there any analysis done on whether availability itself might have been why people weren't using sanitary napkins? These products tend to be less available in rural communities, lower-income areas, or less-educated communities -- could availability itself have been a confounding factor that wasn't explored?

Response 18: We thank the reviewer for this point. Unfortunately, the 2019 Bangladesh MICS dataset does not contain variables on commercial market supply or physical product availability in communities.

Comment 19 (lines 279-281: These textbooks lack essential information on menstruation, the use of sanitary napkins, and guidance on changing intervals, which are critical for maintaining good menstrual hygiene practices.)**:** This adds to my comment throughout questioning the proxy of education to awareness -- this seems to imply that education doesn't equate to awareness. Suggest addressing more directly this tension.

Response 19: We thank the reviewer for highlighting this apparent tension. There are two key points to this:

First, there is a chronological distinction. Our study utilizes 2019 MICS data, representing a period when the national curriculum **did** contain basic components of menstrual health. The omission of these critical components occurred during the recent 2025 national curriculum update. We have now clear out this in line 281.

Second, utilizing educational attainment as a proxy for MHM awareness does not strictly assume that MHM components must be present in the textbooks. Formal schooling builds general health literacy, provides the cognitive tools to process external health information, and fosters crucial peer-to-peer knowledge sharing among adolescent girls.

Comment 20 (lines 296-297: rather, they are embedded within broader contextual factors such as community norms, media exposure, educational content, and policy frameworks.)**:** And also market dynamics -- how available are products?

Response 20: We thank the reviewer for this important point. We acknowledge that market dynamics, including product availability and supply-side factors, are also relevant contextual determinants of menstrual hygiene management. However, as our dataset did not include information on product availability, we were unable to assess this aspect in the present study.

Comment 21 (lines 304: effects of education and wealth)**:** Suggest framing like this more consistently throughout -- rather than around 'awareness.'

Response 21: We thank the reviewer for pointing this out. We have now revised the terms to make them consistent.

Comment 22 (lines 309-311: Second, awareness and affordability were measured using proxy indicators—educational attainment and household wealth index—which may not fully capture the complexity of these constructs.): Suggest expansion on this one.

Response 22: We agree. We have expanded this limitation section to clarify that individual educational attainment does not directly capture specific menstrual health literacy, and the household wealth index does not necessarily reflect an individual woman's financial autonomy or direct purchasing power. Please refer to lines 315-321 in the revised manuscript.

Reviewer 01:

Comment 01: Overall Evaluation

This manuscript addresses a highly relevant public health issue, menstrual hygiene management (MHM) by examining the relative and combined influence of awareness and affordability on sanitary napkin use in Bangladesh. The use of nationally representative MICS data and application of regression and marginal probability analyses are notable strengths. The study provides valuable insights for policy and program design in low-resource settings. However, despite its strengths, the manuscript requires major revision due to conceptual oversimplification, methodological limitations (particularly proxy measurement), insufficient critical interpretation, and language inconsistencies. Strengthening these areas will significantly enhance the rigor and contribution of the study.

Response 01: We sincerely thank the reviewer for their encouraging assessment of our study's strengths and its relevance to public health. We have carefully considered this feedback and have addressed the concerns accordingly. Please find our detailed, point-by-point responses to each specific comment below.

Comment 02: Title and Abstract

The title is clear, engaging, and directly reflects the core research question, effectively highlighting the comparison between awareness and affordability. The abstract is well-structured and summarizes the background, methods, findings, and conclusions.

However, it remains largely descriptive and lacks emphasis on novelty and theoretical contribution. The use of education and wealth index as proxies for awareness and affordability, respectively, is mentioned but not critically justified in the abstract. Additionally, the conclusion may slightly overstate the causal implication, given the cross-sectional design. The abstract would benefit from a clearer statement of the research gap and a more cautious interpretation of findings.

Response 02: We thank the reviewer for this constructive feedback. We have revised the Abstract to explicitly state the research gap (the lack of studies exploring the interactive effects of awareness and affordability) and provided a brief justification for using education and wealth index as operational proxies.

Comment 03: Introduction

The introduction provides a comprehensive overview of menstruation, MHM, and its global and national significance. It effectively contextualizes the issue within Bangladesh using relevant statistics and literature. The discussion of stigma, health outcomes, and social implications is appropriate and well-supported. Importantly, the authors identify a research gap regarding the interaction between awareness and affordability, which is a strong aspect of the manuscript.

However, the section could be improved by tightening its structure and reducing redundancy. Some paragraphs are overly descriptive and could be more concise. Additionally, while the research gap is mentioned, it could be articulated more sharply to emphasize the novelty of the interaction analysis. The conceptual distinction between awareness (education) and affordability (wealth index) should also be more critically justified, as these proxies may not fully capture the constructs.

Response 03: We thank the reviewer for the positive evaluation. We have significantly tightened this section and sharpened the description of the research gap (lines 95-102). For the proxy, we have broaden the justification of using these indicators in the method section (lines 131-138).

Comment 04: Methods

The methods section is generally well-organized and provides sufficient detail about the data source, sampling design, and analytical approach. The use of nationally representative MICS data strengthens the external validity of the findings. The application of generalized linear models and marginal probability estimation is appropriate and enhances interpretability.

However, there are several important limitations. First, the study relies on proxy variables, education for awareness and wealth index for affordability which may not accurately reflect the intended constructs. This introduces potential measurement bias and should be more critically discussed. Second, the cross-sectional design limits causal inference, yet the manuscript occasionally implies causality. Third, although survey weights are applied, there is limited discussion on handling potential confounding or multicollinearity. Finally, the binary classification of sanitary napkin use as a proxy for "proper MHM" may oversimplify menstrual practices, as other safe alternatives (e.g., menstrual cups) are not considered.

Response 04: We thank the reviewer for confirming the appropriateness of our analytical approach. We completely agree with the identified limitations. As noted, because this study is a secondary analysis of survey data, these constraints are structural. We have fully acknowledged and critically discussed each of these points in the Strengths and Limitations section of the discussion.

Comment 05: Results

The results are clearly presented, with appropriate use of tables, figures, and statistical estimates. The descriptive statistics provide a comprehensive overview of the sample characteristics. The regression analysis and adjusted odds ratios are well reported, and the inclusion of marginal probabilities is a strong addition that improves interpretability. The spatial analysis (district-level mapping) adds value by highlighting geographic disparities. Figures (e.g., Fig 1 and Fig 2) effectively illustrate differences in sanitary napkin use across regions and socioeconomic groups. The finding that education (awareness) may outweigh wealth (affordability) in certain contexts is particularly interesting.

However, the results section is somewhat descriptive and could benefit from clearer emphasis on key findings. Additionally, some interpretations are presented within the results rather than reserved for the discussion. Greater clarity in linking statistical findings to the research question would improve this section.

Response 05: We thank the reviewer for the highly positive feedback on our presentation of the results. To address the concern regarding descriptive tone and interpretation, we have carefully re-reviewed the Results section to ensure that statistical findings are tied directly to our core research questions and that any explanatory interpretation is strictly reserved for the discussion section.

Comment 06: Discussion

The discussion appropriately interprets the findings and situates them within existing literature. The emphasis on the interaction between awareness and affordability is a key strength, and the discussion of community, household, and individual-level factors provides a multi-level perspective.

However, the discussion remains somewhat descriptive and lacks deeper critical analysis. The manuscript would benefit from stronger theoretical framing, for example by linking findings to behavior change theories or health access models. While policy implications are mentioned, they are not sufficiently developed or specific. The claim that awareness has a stronger effect than affordability should be interpreted more cautiously, considering the limitations of proxy variables.

The section on policy and program interventions (e.g., VAT policy, curriculum gaps, and Ritu program) is informative but could be more directly linked to the study findings. Additionally, there is some repetition across paragraphs, and the overall structure could be more concise.

Response 06: We have revised the Discussion to provide a more critical analysis. We integrated two theoretical frameworks, the Andersen Model of Health Services Use and Social Cognitive Theory, to better explain the multi-level factors shaping MHM behaviors. Per your suggestion, we now interpret the comparison between awareness and affordability more cautiously, acknowledging the limitations of our proxy variables. Finally, we directly linked policy interventions (VAT and curriculum gaps) to our empirical findings and streamlined the section to improve conciseness.

Comment 07: Strengths and Limitations

The authors acknowledge several strengths, including the use of nationally representative data and advanced statistical methods. The discussion of limitations is appropriate, particularly regarding the cross-sectional design and use of proxy variables.

However, this section could be expanded further. For instance, the potential for residual confounding, measurement error, and reporting bias should be more explicitly discussed. The limitation regarding the binary outcome variable and exclusion of other menstrual products should also be highlighted more clearly.

Response 07: We thank the reviewer for these excellent suggestions. We have expanded our study limitations section to explicitly discuss the potential impacts of residual confounding (lines 313-315) and proxy-related measurement error (315-321).

Comment 08: Conclusion

The conclusion effectively summarizes the main findings and reinforces the importance of addressing both awareness and affordability. The recommendation for integrated interventions is appropriate and aligns with the study results.

However, the conclusion could be strengthened by providing more specific and actionable policy recommendations. Additionally, the language should be slightly moderated to avoid implying causality, given the observational nature of the study.

Response 08: We thank the reviewer for this constructive feedback. We have revised the Conclusion to provide specific, actionable policy recommendations.

Comment 09: References

The reference list is comprehensive and includes relevant and recent literature. The citations are generally appropriate and support the arguments made in the manuscript. However, minor inconsistencies in formatting should be corrected to ensure compliance with journal guidelines.

Response 09: We appreciate the reviewer's comment. We have thoroughly reviewed the reference list and corrected all formatting inconsistencies.

Comment 10: Language and Writing Quality

The manuscript is generally well-written and understandable. However, there are several grammatical errors, typographical issues, and inconsistencies in terminology. Some sentences are overly long or repetitive. Professional language editing is recommended to improve clarity and readability.

Response 10: We thank the reviewer for this note. The entire manuscript has undergone comprehensive proofreading and professional language editing to eliminate grammatical errors, fix typographical issues, and break down overly long or repetitive sentences.
