# Supplementary material for: The combined role of education and wealth in menstrual health: Evidence from Bangladesh
Source: PLoS One. 2026 Oct 5;21(10):e0346457. doi: 10.1371/journal.pone.0346457 (PMC13637891; doi:10.1371/journal.pone.0346457)
Supplement: Peer review history

# Peer review: Decision Letter 0

**DOI:** 10.1371/journal.pone.0346457.r001

**Authors:** Alison Parker

5 May 2026

PONE-D-26-13387

Awareness or Affordability: What matters the most in ensuring menstrual hygiene management?

PLOS One?

Dear Dr. Patwary,

plosone@plos.org. When you're ready to submit your revision, log on to https://www.editorialmanager.com/pone/ and select the 'Submissions Needing Revision' folder to locate your manuscript file.

- A letter that responds to each point raised by the academic editor and reviewer(s). You should upload this letter as a separate file labeled 'Response to Reviewers'.
- A marked-up copy of your manuscript that highlights changes made to the original version. You should upload this as a separate file labeled 'Revised Manuscript with Track Changes'.
- An unmarked version of your revised paper without tracked changes. You should upload this as a separate file labeled 'Manuscript'.

If you would like to make changes to your financial disclosure, please include your updated statement in your cover letter. Guidelines for resubmitting your figure files are available below the reviewer comments at the end of this letter.

If applicable, we recommend that you deposit your laboratory protocols in protocols.io to enhance the reproducibility of your results. Protocols.io assigns your protocol its own identifier (DOI) so that it can be cited independently in the future. For instructions see: https://journals.plos.org/plosone/s/submission-guidelines#loc-laboratory-protocols. Additionally, PLOS ONE offers an option for publishing peer-reviewed Lab Protocol articles, which describe protocols hosted on protocols.io. Read more information on sharing protocols at https://plos.org/protocols?utm_medium=editorial-email&utm_source=authorletters&utm_campaign=protocols.

As the corresponding author, your ORCID iD is verified in the submission system and will appear in the published article. PLOS supports the use of ORCID, and we encourage all coauthors to register for an ORCID iD and use it as well. Please encourage your coauthors to verify their ORCID iD within the submission system before final acceptance, as unverified ORCID iDs will not appear in the published article. *Only*  the individual author can complete the verification step; PLOS staff *cannot*  verify ORCID iDs on behalf of authors.

We look forward to receiving your revised manuscript.

Kind regards,

Alison Parker

Academic Editor

PLOS One

Journal Requirements:

When submitting your revision, we need you to address these additional requirements.

1. Please ensure that your manuscript meets PLOS ONE's style requirements, including those for file naming. The PLOS ONE style templates can be found at

https://journals.plos.org/plosone/s/file?id=wjVg/PLOSOne_formatting_sample_main_body.pdf and

https://journals.plos.org/plosone/s/file?id=ba62/PLOSOne_formatting_sample_title_authors_affiliations.pdf

2. Thank you for stating the following in your Competing Interests section:  [None declared].

Please complete your Competing Interests on the online submission form to state any Competing Interests. If you have no competing interests, please state ""The authors have declared that no competing interests exist."", as detailed online in our guide for authors at http://journals.plos.org/plosone/s/submit-now

This information should be included in your cover letter; we will change the online submission form on your behalf.

3. Please note that your Data Availability Statement is currently missing the DOI/accession number of each dataset OR a direct link to access each database. If your manuscript is accepted for publication, you will be asked to provide these details on a very short timeline. We therefore suggest that you provide this information now, though we will not hold up the peer review process if you are unable.

4. We note that Figure 1 in your submission contain [map/satellite] images which may be copyrighted. All PLOS content is published under the Creative Commons Attribution License (CC BY 4.0), which means that the manuscript, images, and Supporting Information files will be freely available online, and any third party is permitted to access, download, copy, distribute, and use these materials in any way, even commercially, with proper attribution. For these reasons, we cannot publish previously copyrighted maps or satellite images created using proprietary data, such as Google software (Google Maps, Street View, and Earth). For more information, see our copyright guidelines: http://journals.plos.org/plosone/s/licenses-and-copyright.

We require you to either (1) present written permission from the copyright holder to publish these figures specifically under the CC BY 4.0 license, or (2) remove the figures from your submission:

1. You may seek permission from the original copyright holder of Figure 1 to publish the content specifically under the CC BY 4.0 license.

We recommend that you contact the original copyright holder with the Content Permission Form (http://journals.plos.org/plosone/s/file?id=7c09/content-permission-form.pdf) and the following text:

“I request permission for the open-access journal PLOS ONE to publish XXX under the Creative Commons Attribution License (CCAL) CC BY 4.0 (http://creativecommons.org/licenses/by/4.0/). Please be aware that this license allows unrestricted use and distribution, even commercially, by third parties. Please reply and provide explicit written permission to publish XXX under a CC BY license and complete the attached form.”

Please upload the completed Content Permission Form or other proof of granted permissions as an ""Other"" file with your submission.

In the figure caption of the copyrighted figure, please include the following text: “Reprinted from [ref] under a CC BY license, with permission from [name of publisher], original copyright [original copyright year].”

2. If you are unable to obtain permission from the original copyright holder to publish these figures under the CC BY 4.0 license or if the copyright holder’s requirements are incompatible with the CC BY 4.0 license, please either i) remove the figure or ii) supply a replacement figure that complies with the CC BY 4.0 license. Please check copyright information on all replacement figures and update the figure caption with source information. If applicable, please specify in the figure caption text when a figure is similar but not identical to the original image and is therefore for illustrative purposes only.

The following resources for replacing copyrighted map figures may be helpful:

USGS National Map Viewer (public domain): http://viewer.nationalmap.gov/viewer/

The Gateway to Astronaut Photography of Earth (public domain): http://eol.jsc.nasa.gov/sseop/clickmap/

Maps at the CIA (public domain): https://www.cia.gov/library/publications/the-world-factbook/index.html and https://www.cia.gov/library/publications/cia-maps-publications/index.html

NASA Earth Observatory (public domain): http://earthobservatory.nasa.gov/

Landsat: http://landsat.visibleearth.nasa.gov/

USGS EROS (Earth Resources Observatory and Science (EROS) Center) (public domain): http://eros.usgs.gov/#

Natural Earth (public domain): http://www.naturalearthdata.com/

5. Please include captions for your Supporting Information files at the end of your manuscript, and update any in-text citations to match accordingly. Please see our Supporting Information guidelines for more information: http://journals.plos.org/plosone/s/supporting-information.

6. If the reviewer comments include a recommendation to cite specific previously published works, please review and evaluate these publications to determine whether they are relevant and should be cited. There is no requirement to cite these works unless the editor has indicated otherwise.

[Note: HTML markup is below. Please do not edit.]

Reviewers' comments:

Reviewer's Responses to Questions

**Comments to the Author**

1. Is the manuscript technically sound, and do the data support the conclusions?

Reviewer #1: Yes

Reviewer #2: Yes

Reviewer #3: Partly

**********

2. Has the statistical analysis been performed appropriately and rigorously?

Reviewer #1: Yes

Reviewer #2: Yes

Reviewer #3: Yes

**********

3. Have the authors made all data underlying the findings in their manuscript fully available?

The PLOS Data policy

Reviewer #1: Yes

Reviewer #2: Yes

Reviewer #3: Yes

**********

4. Is the manuscript presented in an intelligible fashion and written in standard English?

Reviewer #1: Yes

Reviewer #2: Yes

Reviewer #3: Yes

**********

Reviewer #1: Overall Evaluation

This manuscript addresses a highly relevant public health issue, menstrual hygiene management (MHM) by examining the relative and combined influence of awareness and affordability on sanitary napkin use in Bangladesh. The use of nationally representative MICS data and application of regression and marginal probability analyses are notable strengths. The study provides valuable insights for policy and program design in low-resource settings. However, despite its strengths, the manuscript requires major revision due to conceptual oversimplification, methodological limitations (particularly proxy measurement), insufficient critical interpretation, and language inconsistencies. Strengthening these areas will significantly enhance the rigor and contribution of the study.

Title and Abstract

The title is clear, engaging, and directly reflects the core research question, effectively highlighting the comparison between awareness and affordability. The abstract is well-structured and summarizes the background, methods, findings, and conclusions. However, it remains largely descriptive and lacks emphasis on novelty and theoretical contribution. The use of education and wealth index as proxies for awareness and affordability, respectively, is mentioned but not critically justified in the abstract. Additionally, the conclusion may slightly overstate the causal implication, given the cross-sectional design. The abstract would benefit from a clearer statement of the research gap and a more cautious interpretation of findings.

Introduction

The introduction provides a comprehensive overview of menstruation, MHM, and its global and national significance. It effectively contextualizes the issue within Bangladesh using relevant statistics and literature. The discussion of stigma, health outcomes, and social implications is appropriate and well-supported. Importantly, the authors identify a research gap regarding the interaction between awareness and affordability, which is a strong aspect of the manuscript.

However, the section could be improved by tightening its structure and reducing redundancy. Some paragraphs are overly descriptive and could be more concise. Additionally, while the research gap is mentioned, it could be articulated more sharply to emphasize the novelty of the interaction analysis. The conceptual distinction between awareness (education) and affordability (wealth index) should also be more critically justified, as these proxies may not fully capture the constructs.

Methods

The methods section is generally well-organized and provides sufficient detail about the data source, sampling design, and analytical approach. The use of nationally representative MICS data strengthens the external validity of the findings. The application of generalized linear models and marginal probability estimation is appropriate and enhances interpretability.

However, there are several important limitations. First, the study relies on proxy variables, education for awareness and wealth index for affordability which may not accurately reflect the intended constructs. This introduces potential measurement bias and should be more critically discussed. Second, the cross-sectional design limits causal inference, yet the manuscript occasionally implies causality. Third, although survey weights are applied, there is limited discussion on handling potential confounding or multicollinearity. Finally, the binary classification of sanitary napkin use as a proxy for “proper MHM” may oversimplify menstrual practices, as other safe alternatives (e.g., menstrual cups) are not considered.

Results

The results are clearly presented, with appropriate use of tables, figures, and statistical estimates. The descriptive statistics provide a comprehensive overview of the sample characteristics. The regression analysis and adjusted odds ratios are well reported, and the inclusion of marginal probabilities is a strong addition that improves interpretability.

The spatial analysis (district-level mapping) adds value by highlighting geographic disparities. Figures (e.g., Fig 1 and Fig 2) effectively illustrate differences in sanitary napkin use across regions and socioeconomic groups. The finding that education (awareness) may outweigh wealth (affordability) in certain contexts is particularly interesting.

However, the results section is somewhat descriptive and could benefit from clearer emphasis on key findings. Additionally, some interpretations are presented within the results rather than reserved for the discussion. Greater clarity in linking statistical findings to the research question would improve this section.

Discussion

The discussion appropriately interprets the findings and situates them within existing literature. The emphasis on the interaction between awareness and affordability is a key strength, and the discussion of community, household, and individual-level factors provides a multi-level perspective.

However, the discussion remains somewhat descriptive and lacks deeper critical analysis. The manuscript would benefit from stronger theoretical framing, for example by linking findings to behavior change theories or health access models. While policy implications are mentioned, they are not sufficiently developed or specific. The claim that awareness has a stronger effect than affordability should be interpreted more cautiously, considering the limitations of proxy variables.

The section on policy and program interventions (e.g., VAT policy, curriculum gaps, and Ritu program) is informative but could be more directly linked to the study findings. Additionally, there is some repetition across paragraphs, and the overall structure could be more concise.

Strengths and Limitations

The authors acknowledge several strengths, including the use of nationally representative data and advanced statistical methods. The discussion of limitations is appropriate, particularly regarding the cross-sectional design and use of proxy variables.

However, this section could be expanded further. For instance, the potential for residual confounding, measurement error, and reporting bias should be more explicitly discussed. The limitation regarding the binary outcome variable and exclusion of other menstrual products should also be highlighted more clearly.

Conclusion

The conclusion effectively summarizes the main findings and reinforces the importance of addressing both awareness and affordability. The recommendation for integrated interventions is appropriate and aligns with the study results.

However, the conclusion could be strengthened by providing more specific and actionable policy recommendations. Additionally, the language should be slightly moderated to avoid implying causality, given the observational nature of the study.

References

The reference list is comprehensive and includes relevant and recent literature. The citations are generally appropriate and support the arguments made in the manuscript. However, minor inconsistencies in formatting should be corrected to ensure compliance with journal guidelines.

Language and Writing Quality

The manuscript is generally well-written and understandable. However, there are several grammatical errors, typographical issues, and inconsistencies in terminology. Some sentences are overly long or repetitive. Professional language editing is recommended to improve clarity and readability.

Reviewer #2: Thank you for the opportunity to review this article. It definitely adds to the literature on this very important topic. Methodology is sound and conclusions are apt. I would work some key references into the intro and discussion, as they would add breadth and depth to this article and expand its reach. The other comments are merely grammatical.

Abstract-

Methods- “We used a nationally representative data from the 2019 Bangladesh Multiple Indicator Cluster Survey (MICS).”

Same comment for Methods, p1, line 21

P2, line 45-46: “Menstruation is a natural physiological process that generally indicates the onset of the reproductive 46 lifespan for a girl,…”

P2, line 47: Menarche does not need to be capitalized

P2, line 48- 12 to 15 years

P21, line 288, “aged 10-15 years”

I would work these papers into the discussion:

Mittermeier, T ∙ Hawkey, A ∙ Bush, D et al. Menstrual health symptoms and literacy among young women in Aotearoa New Zealand: a nationwide cross-sectional survey. J Pediatr Adolesc Gynecol. 2025; 38:328-335

Bhoda, K ∙ Burgoa, S ∙ Zerrouki, Y ...Examining Menstrual Health and Hygiene Educational Initiatives for American Middle and High School Menstruating Students: A Scoping Review.

J Pediatr Adolesc Gynec. 2024; 37(5):465-476

Harrison, ME ∙ Davies, S ∙ Tyson, N ...NASPAG Position Statement: Eliminating Period Poverty in Adolescents and Young Adults Living in North America. J Pediatr Adolesc Gynecol. 2022; 35(6):609-611

Davies, S ∙ Darien, K ∙ Bazyar, F ...A Multimodal Qualitative Approach to Understanding Menstrual Health Equity among Adolescents and Young Adults. Journal of Pediatric and Adolescent Gynecology. 2023; 36(6):511-517

Armour M, Hyman MK et al. Menstrual Health Literacy and Management Strategies in Young Women in Australia: A National Online Survey of Young Women Aged 13-25 Years. Journal of Pediatric and Adolescent GynecologyVol. 34Issue 2p135–143Published online: November 11, 2020

Could also add this article, although it is a review:

Rome ES, Tyson N. Menstrual Equity. Obstet Gynecol Clin North Am. 2024 Dec;51(4):731-744. doi: 10.1016/j.ogc.2024.08.008. Epub 2024 Sep 21.

Reviewer #3: Thank you for this study analysis and for this manuscript. The authors are addressing a critical question about the relationship between awareness and affordability in driving use of menstrual products. In the attachment, I'm including a more detailed review with comments, but want to suggest a few overarching revisions to strengthen this manuscript that would strengthen the analysis/discussion:

(1) I would love to see a stronger discussion of sanitary napkin use. How are we defining sanitary napkins -- is this both reusable and single use products? Only those that are purpose made? Are we referring only to sanitary napkins or to all menstrual products -- and if only sanitary napkins, why not the full range of products? Finally, when asking about use are we asking about *sufficient* use for the full menstrual period (e.g. enough product to cover all days of menstruation with appropriate washing/disposal) or does this also capture people who menstruate who are using perhaps 1-4 pads and then resorting to cloth or fabric because they do not have sufficient product to cover the cycle?

(2) I think that the manuscript needs a much stronger discussion of why education is a proxy for awareness. This also requires better defining awareness. Are you talking about awareness of menstruation as a biological function? Awareness of safe methods of menstrual health management? Awareness of products and how to access and use them? All of the above? Then, within this context, I think that you could either (1) expand on why educational attainment translates into better awareness of menstrual health/menstrual products. This would require addressing some of the contradictions within the manuscript that seem to suggest that schools do not adequately teach about menstrual health. Or, (2) I think that you could re-work the paper to focus on educational attainment and affordability as intertwined factors, but then layer in how educational attainment translates to awareness within the discussion section. As-is, I am left questioning the education as a proxy for awareness, particularly since the paper highlights many barriers to menstrual health education within schools. I'm also curious in the individual factors/discussion as to why media exposure might not also translate into product usage. Perhaps a third alternative approach here would be to develop multiple proxy factors for awareness -- might educational attainment, community prevalence, and media exposure translate more holistically into a better articulation of awareness? This would require re-working the statistical analysis and model, but could be a valuable, more holistic way of looking at awareness.

(3) I think that a more rigorous analysis/discussion of the individual factors would be valuable. In particular, I think that you need to explore product availability as a potential confounding factor. If products themselves are not available in particular communities, either due to geography (rural v urban), religious beliefs, etc. this creates a barrier to product use that education and affordability cannot solve. This requires much more discussion and explanation for how this is controlled for. I also think that better articulating how the statistical analysis controlled for factors like age, religious belief, rural vs urban, and other individual factors would be valuable. This is particularly important because age and educational attainment intersect so deeply (young girls are much less likely to have high educational attainment). To make sure that it's clear to the reader that you have accounted for these intricacies, additional discussion would be valuable.

(4) I think that it would be helpful to further explore the stigma/taboo elements. More discussion is needed as to whether the authors think that stigma/taboo are influenced by awareness, and if so, how? Or whether stigma/taboo serve as an additional added individual/community factor that needs to be overcome in addition to awareness and affordability.

(5) I think that the language throughout the manuscript should be updated to avoid placing blame on people who menstruate. There are a few references to poor menstrual hygiene management or improper menstrual hygiene management. I suggest re-framing these to better articulate systemic failures/barriers to menstrual health, rather than individual failures to use products.

(6) Finally, I think that a title modification might be valuable. Since your key takeaway is essentially that both awareness and affordability matter for menstrual health, positing them as potentially conflicting doesn't resonate as much to me. But, perhaps the intention is to be evocative, since most readers will assume they both matter!

**********

what does this mean?). If published, this will include your full peer review and any attached files.

If you choose “no”, your identity will remain anonymous but your review may still be made public.

**Do you want your identity to be public for this peer review?**  For information about this choice, including consent withdrawal, please see our Privacy Policy

Reviewer #1: **Yes:**  Dr. Md Nahid Uz Zaman, PhD, RN

Reviewer #2: No

Reviewer #3: No

**********

[NOTE: If reviewer comments were submitted as an attachment file, they will be attached to this email and accessible via the submission site. Please log into your account, locate the manuscript record, and check for the action link "View Attachments". If this link does not appear, there are no attachment files.]

To ensure your figures meet our technical requirements, please review our figure guidelines: https://journals.plos.org/plosone/s/figures

You may also use PLOS’s free figure tool, NAAS, to help you prepare publication quality figures: https://journals.plos.org/plosone/s/figures#loc-tools-for-figure-preparation.

NAAS will assess whether your figures meet our technical requirements by comparing each figure against our figure specifications.

Attachment Submitted filename: Review Report_Plos one.pdf

Attachment Submitted filename: PONE-D-26-13387_Peer Reviewer Comments.pdf

# Author comment: Author response to Decision Letter 1

**DOI:** 10.1371/journal.pone.0346457.r002

11 Jun 2026

Awareness or Affordability: What matters the most in ensuring menstrual hygiene management?

Rebuttal Letter

Reviewer 03.

Comment 1 (line 17: awareness regarding menstrual hygiene): This will require a more detailed definition in the paper -- awareness of menstrual hygiene from a health/biological standpoint? Management of menstrual hygiene/product use? Options for products? What do we mean by awareness here?

Response 1: We have now clear out what do we meant by awareness here (knowledge regarding the menstrual hygiene management) and also broaden the explanation in the explanatory variables part (lines 131).

Comment 2 (line 23: individual educational attainment): I'd be curious to see more justifications around the selection of this proxy -- e.g. some sort of evidence/justification around school/educational facilities being the critical entry point to awareness around menstrual products.

Response 2: We thank the reviewer for this important comment. We have justified the use of this proxy in the explanatory variable part (lines 131-134).

Comment 3 (line 31: sanitary napkins): Were only sanitary napkins assessed? Or was this meant to be inclusive of the full range of menstrual products?

Response 3: The term “sanitary napkins” refers to reported use of sanitary pads during the last menstrual period, rather than assessment of a full range of individual product types separately (please see the updated outcome variable section; lines 122-125).

Comment 4 (line 58: This poor management of menstrual hygiene): Suggest re-framing to a way that doesn't place blame on the person who menstruates -- there has largely been a shift away from framing around 'menstrual hygiene' and towards 'menstrual health' for this reason. In this framing, it sounds like the person who menstruates is to blame for these health outcomes, rather than the system that has failed them.

Response 4: We thank the reviewer for this important suggestion. We have revised the text accordingly (lines 56-57).

Comment 5 (line 81-82: only a quarter of adolescent girls know about menstruation before their menarche): This seems to suggest that menstrual health is not taught in schools, which feels counter to the proxy indicator of educational status to indicate awareness about menstrual health/menstrual products.

Response 5: We thank the reviewer for this sharp observation. We agree that the low rate of pre-menarche knowledge seems contradictory at first glance. This statistic reflects a well-documented reality in Bangladesh: reproductive health topics are often introduced too late in the curriculum (or skipped altogether in primary school), leaving young girls uninformed at the actual onset of menarche. However, our proxy of "educational attainment" for women aged 15–49 measures their cumulative exposure to schooling. As women progress into secondary and higher education, they gain delayed but crucial exposure to health curricula. Thus, while early school fails to prepare them before menarche, longer retention in the education system is still the primary driver of MHM awareness.

Comment 6 (line 117: The survey collected detailed information from women of reproductive age (15–49 years)): Suggest acknowledging the gap on young adolescent data -- the average age of menarche is much younger than 15, which means that we aren't capturing the full menstruating population with these data. This is hard to address since MICS / survey data don't include this, but suggest highlighting this potential gap.

Response 6: We have added this as a limitation (lines 325-327).

Comment 7 (line 125-127: In this study, sanitary napkin use was considered a proxy for proper menstrual hygiene, as they are typically cleaner, safer, and more absorbent than traditional or improvised materials): Better define what is meant by sanitary napkin and whether this includes both reusable and disposable options or just disposable/single use. Include context on whether cloth/non-purpose made materials were considered to 'count' as sanitary napkins in this definition.

Response 7: We thank the reviewer for this comment. Sanitary napkin use is defined as any reported use of purpose-made sanitary pads during the last menstrual period, regardless of exclusive or combined use (refer to the updated outcome variable section).

Comment 8 (line 128): An additional variable that should be part of this analysis is actual availability of products -- availability itself is often a barrier and if sanitary napkins are not readily available in certain communities (most often, rural or vulnerable communities, areas with lower household wealth, etc), this creates a confounding variable that influences outcome. Suggest adding analysis on this that speaks to how readily available products are in these regions... beyond just actual use of products. Use of products and prevalence of community use still rely on products being available to purchase and use.

Response 8: We thank the reviewer for this point. Unfortunately, the 2019 Bangladesh MICS dataset does not contain variables on commercial market supply or physical product availability in communities.

Comment 9 (line 135-137: Individual level factors that can affect use of sanitary napkin included participants age, educational attainment, exposure to media, and status of disability): Suggest acknowledging that you are missing a critical age group within these data, since the data begin at age 15 but average age of menarche is much lower. I would acknowledge this and contextualize a bit further.

Response 9: We have added this as a limitation (lines 325-327).

Comment 10 (Table 1: The community prevalence of sanitary napkins use is below 30%): Contextualize what this includes. Can you also clarify how prevalence is defined here? Is it a simple yes/no on access to sanitary pads, or does it measure for *sufficient* access, e.g. sufficient product access for the full cycle, rather than supplementation with non-purpose made products (e.g. 1-4 sanitary pads per cycle, supplemented with cloth or other materials).

Response 10: We thank the reviewer for this comment. The community prevalence was built on to the binary outcome defined in the outcome variable section, where sanitary napkin use was measured as any reported use during the last menstrual period (yes/no), regardless of quantity or whether other materials were also used (lines 144-146).

Comment 11 (lines 192-194: Notable findings include a higher likelihood of using sanitary napkins in younger age groups, with substantial increases in odds ratios from 25-29 years (AOR=2.57) to 15-19 years (AOR=6.09)): This seems counter to the findings that higher educational levels result in higher likelihood of use, as most women/girls with higher education are likely to be older than those with lower.

Response 11: We thank the reviewer for this important comment. The findings reflect two different effects estimated in the adjusted model. Age captures cohort differences in menstrual hygiene practices, where younger women are more likely to adopt sanitary napkins due to more recent exposure to menstrual health information and changing norms. Education, on the other hand, reflects individual awareness and knowledge, which increases the likelihood of sanitary napkin use across all age groups. Therefore, higher use among younger women and more educated women are not contradictory, but represent separate pathways influencing behavior.

Comment 12 (lines 206-207: educational attainment significantly increases the likelihood of using sanitary napking among women aged 15-49 years in Bangladesh): I think that this is a more powerful finding than framing the findings around educational attainment as being a proxy for 'awareness.'

Response 12: We agree with the reviewer that formal educational attainment is a powerful determinant with broader societal implications beyond acting as a proxy for awareness. However, to keep the paper tightly aligned with our research question, our analysis and discussion focus specifically on its role in health awareness and information access.

Comment 13 (lines 216-217: This study highlights the significant roles of both awareness and affordability in determining sanitary napkin use among women in Bangladesh): For this takeaway to be highlighted, I think that much more work is needed to do justify educational attainment as a proxy for awareness. I think that the stronger framing is that educational attainment is significant in determining sanitary napkin use -- and a potential policy takeaway/discussion lens could be unpacking how education might lead to increased awareness. I also think that we need much more contextual definition on what is meant by awareness here.

Response 13: We thank the reviewer for this observation. As detailed in our Explanatory Variables (lines 131-138) and Discussion sections (lines 256-259), we use formal education as an established proxy for health awareness because higher educational attainment is a primary structural pathway through which women access health literacy and information on menstrual hygiene.

Comment 14 (lines 225: Comparison with earlier studies): Is this the right header here? This seems to be looking at other individual factors within the context of this study, rather than prior studies. Additional studies are woven in to this discussion, but suggest re-framing the header to focus more on the other individual factors as the focus of this section.

Response 14: We thank the reviewer for this helpful suggestion. We have revised the header to “Factors influencing sanitary napkin use”.

Comment 15 (lines 232: proper hygiene practices.): Avoid framing like 'proper' hygiene practices -- this shifts blame to the person menstruating rather than a system that has failed her.

Response 15: We thank the reviewer for this insightful comment. We agree that the term "proper" can inadvertently imply individual blame. We have revised the manuscript to replace "proper" with "recommended" to focus on the practices themselves rather than casting judgment on the individual.

Comment 16 (lines 255-258): I think that this context needs to come much earlier in the paper to help readers understand what is meant by awareness -- and why education might be a proxy for it.

Response 16: We thank the reviewer for the suggestion. In the revised manuscript, we have broadened the rationale for using these proxies (lines 131-138).

Comment 17 (lines 258-261): This next part seems to focus more on stigma/shame/taboo than on awareness. I would suggest either (1) expanding on why/how awareness reduces stigma/shame and therefore overcomes this; or (2) saying that even with increased awareness, stigma/shame pose further barriers to access.

Response 17: We thank the reviewer for this helpful suggestion. We have revised the Discussion (according to option 2) to clarify that (lines 262-264).

Comment 18 (lines 263: availability): Did your statistical analysis take this into account at all? E.g. was there any analysis done on whether availability itself might have been why people weren't using sanitary napkins? These products tend to be less available in rural communities, lower-income areas, or less-educated communities -- could availability itself have been a confounding factor that wasn't explored?

Response 18: We thank the reviewer for this point. Unfortunately, the 2019 Bangladesh MICS dataset does not contain variables on commercial market supply or physical product availability in communities.

Comment 19 (lines 279-281: These textbooks lack essential information on menstruation, the use of sanitary napkins, and guidance on changing intervals, which are critical for maintaining good menstrual hygiene practices.): This adds to my comment throughout questioning the proxy of education to awareness -- this seems to imply that education doesn't equate to awareness. Suggest addressing more directly this tension.

Response 19: We thank the reviewer for highlighting this apparent tension. There are two key points to this:

First, there is a chronological distinction. Our study utilizes 2019 MICS data, representing a period when the national curriculum did contain basic components of menstrual health. The omission of these critical components occurred during the recent 2025 national curriculum update. We have now clear out this in line 281.

Second, utilizing educational attainment as a proxy for MHM awareness does not strictly assume that MHM components must be present in the textbooks. Formal schooling builds general health literacy, provides the cognitive tools to process external health information, and fosters crucial peer-to-peer knowledge sharing among adolescent girls.

Comment 20 (lines 296-297: rather, they are embedded within broader contextual factors such as community norms, media exposure, educational content, and policy frameworks.): And also market dynamics -- how available are products?

Response 20: We thank the reviewer for this important point. We acknowledge that market dynamics, including product availability and supply-side factors, are also relevant contextual determinants of menstrual hygiene management. However, as our dataset did not include information on product availability, we were unable to assess this aspect in the present study.

Comment 21 (lines 304: effects of education and wealth): Suggest framing like this more consistently throughout -- rather than around 'awareness.'

Response 21: We thank the reviewer for pointing this out. We have now revised the terms to make them consistent.

Comment 22 (lines 309-311: Second, awareness and affordability were measured using proxy indicators—educational attainment and household wealth index—which may not fully capture the complexity of these constructs.): Suggest expansion on this one.

Response 22: We agree. We have expanded this limitation section to clarify that individual educational attainment does not directly capture specific menstrual health literacy, and the household wealth index does not necessarily reflect an individual woman's financial autonomy or direct purchasing power. Please refer to lines 315-321 in the revised manuscript.

Reviewer 01:

Comment 01: Overall Evaluation

This manuscript addresses a highly relevant public health issue, menstrual hygiene management (MHM) by examining the relative and combined influence of awareness and affordability on sanitary napkin use in Bangladesh. The use of nationally representative MICS data and application of regression and marginal probability analyses are notable strengths. The study provides valuable insights for policy and program design in low-resource settings. However, despite its strengths, the manuscript requires major revision due to conceptual oversimplification, methodological limitations (particularly proxy measurement), insufficient critical interpretation, and language inconsistencies. Strengthening these areas will significantly enhance the rigor and contribution of the study.

Response 01: We sincerely thank the reviewer for their encouraging assessment of our study's strengths and its relevance to public health. We have carefully considered this feedback and have addressed the concerns accordingly. Please find our detailed, point-by-point responses to each specific comment below.

Comment 02: Title and Abstract

The title is clear, engaging, and directly reflects the core research question, effectively highlighting the comparison between awareness and affordability. The abstract is well-structured and summarizes the background, methods, findings, and conclusions.

However, it remains largely descriptive and lacks emphasis on novelty and theoretical contribution. The use of education and wealth index as proxies for awareness and affordability, respectively, is mentioned but not critically justified in the abstract. Additionally, the conclusion may slightly overstate the causal implication, given the cross-sectional design. The abstract would benefit from a clearer statement of the research gap and a more cautious interpretation of findings.

Response 02: We thank the reviewer for this constructive feedback. We have revised the Abstract to explicitly state the research gap (the lack of studies exploring the interactive effects of awareness and affordability) and provided a brief justification for using education and wealth index as operational proxies.

Comment 03: Introduction

The introduction provides a comprehensive overview of menstruation, MHM, and its global and national significance. It effectively contextualizes the issue within Bangladesh using relevant statistics and literature. The discussion of stigma, health outcomes, and social implications is appropriate and well-supported. Importantly, the authors identify a research gap regarding the interaction between awareness and affordability, which is a strong aspect of the manuscript.

However, the section could be improved by tightening its structure and reducing redundancy. Some paragraphs are overly descriptive and could be more concise. Additionally, while the research gap is mentioned, it could be articulated more sharply to emphasize the novelty of the interaction analysis. The conceptual distinction between awareness (education) and affordability (wealth index) should also be more critically justified, as these proxies may not fully capture the constructs.

Response 03: We thank the reviewer for the positive evaluation. We have significantly tightened this section and sharpened the description of the research gap (lines 95-102). For the proxy, we have broaden the justification of using these indicators in the method section (lines 131-138).

Comment 04: Methods

The methods section is generally well-organized and provides sufficient detail about the data source, sampling design, and analytical approach. The use of nationally representative MICS data strengthens the external validity of the findings. The application of generalized linear models and marginal probability estimation is appropriate and enhances interpretability.

However, there are several important limitations. First, the study relies on proxy variables, education for awareness and wealth index for affordability which may not accurately reflect the intended constructs. This introduces potential measurement bias and should be more critically discussed. Second, the cross-sectional design limits causal inference, yet the manuscript occasionally implies causality. Third, although survey weights are applied, there is limited discussion on handling potential confounding or multicollinearity. Finally, the binary classification of sanitary napkin use as a proxy for “proper MHM” may oversimplify menstrual practices, as other safe alternatives (e.g., menstrual cups) are not considered.

Response 04: We thank the reviewer for confirming the appropriateness of our analytical approach. We completely agree with the identified limitations. As noted, because this study is a secondary analysis of survey data, these constraints are structural. We have fully acknowledged and critically discussed each of these points in the Strengths and Limitations section of the discussion.

Comment 05: Results

The results are clearly presented, with appropriate use of tables, figures, and statistical estimates. The descriptive statistics provide a comprehensive overview of the sample characteristics. The regression analysis and adjusted odds ratios are well reported, and the inclusion of marginal probabilities is a strong addition that improves interpretability. The spatial analysis (district-level mapping) adds value by highlighting geographic disparities. Figures (e.g., Fig 1 and Fig 2) effectively illustrate differences in sanitary napkin use across regions and socioeconomic groups. The finding that education (awareness) may outweigh wealth (affordability) in certain contexts is particularly interesting.

However, the results section is somewhat descriptive and could benefit from clearer emphasis on key findings. Additionally, some interpretations are presented within the results rather than reserved for the discussion. Greater clarity in linking statistical findings to the research question would improve this section.

Response 05: We thank the reviewer for the highly positive feedback on our presentation of the results. To address the concern regarding descriptive tone and interpretation, we have carefully re-reviewed the Results section to ensure that statistical findings are tied directly to our core research questions and that any explanatory interpretation is strictly reserved for the discussion section.

Comment 06: Discussion

The discussion appropriately interprets the findings and situates them within existing literature. The emphasis on the interaction between awareness and affordability is a key strength, and the discussion of community, household, and individual-level factors provides a multi-level perspective.

However, the discussion remains somewhat descriptive and lacks deeper critical analysis. The manuscript would benefit from stronger theoretical framing, for example by linking findings to behavior change theories or health access models. While policy implications are mentioned, they are not sufficiently developed or specific. The claim that awareness has a stronger effect than affordability should be interpreted more cautiously, considering the limitations of proxy variables.

The section on policy and program interventions (e.g., VAT policy, curriculum gaps, and Ritu program) is informative but could be more directly linked to the study findings. Additionally, there is some repetition across paragraphs, and the overall structure could be more concise.

Response 06: We have revised the Discussion to provide a more critical analysis. We integrated two theoretical frameworks, the Andersen Model of Health Services Use and Social Cognitive Theory, to better explain the multi-level factors shaping MHM behaviors. Per your suggestion, we now interpret the comparison between awareness and affordability more cautiously, acknowledging the limitations of our proxy variables. Finally, we directly linked policy interventions (VAT and curriculum gaps) to our empirical findings and streamlined the section to improve conciseness.

Comment 07: Strengths and Limitations

The authors acknowledge several strengths, including the use of nationally representative data and advanced statistical methods. The discussion of limitations is appropriate, particularly regarding the cross-sectional design and use of proxy variables.

However, this section could be expanded further. For instance, the potential for residual confounding, measurement error, and reporting bias should be more explicitly discussed. The limitation regarding the binary outcome variable and exclusion of other menstrual products should also be highlighted more clearly.

Response 07: We thank the reviewer for these excellent suggestions. We have expanded our study limitations section to explicitly discuss the potential impacts of residual confounding (lines 313-315) and proxy-related measurement error (315-321).

Comment 08: Conclusion

The conclusion effectively summarizes the main findings and reinforces the importance of addressing both awareness and affordability. The recommendation for integrated interventions is appropriate and aligns with the study results.

However, the conclusion could be strengthened by providing more specific and actionable policy recommendations. Additionally, the language should be slightly moderated to avoid implying causality, given the observational nature of the study.

Response 08: We thank the reviewer for this constructive feedback. We have revised the Conclusion to provide specific, actionable policy recommendations.

Comment 09: References

The reference list is comprehensive and includes relevant and recent literature. The citations are generally appropriate and support the arguments made in the manuscript. However, minor inconsistencies in formatting should be corrected to ensure compliance with journal guidelines.

Response 09: We appreciate the reviewer's comment. We have thoroughly reviewed the reference list and corrected all formatting inconsistencies.

Comment 10: Language and Writing Quality

The manuscript is generally well-written and understandable. However, there are several grammatical errors, typographical issues, and inconsistencies in terminology. Some sentences are overly long or repetitive. Professional language editing is recommended to improve clarity and readability.

Response 10: We thank the reviewer for this note. The entire manuscript has undergone comprehensive proofreading and professional language editing to eliminate grammatical errors, fix typographical issues, and break down overly long or repetitive sentences.

Attachment Submitted filename: Rebuttal Letter_MHM_08May2026.pdf

# Peer review: Decision Letter 1

**DOI:** 10.1371/journal.pone.0346457.r003

**Authors:** Alison Parker

20 Jul 2026

PONE-D-26-13387R1

Awareness or Affordability: What matters the most in ensuring menstrual hygiene?

PLOS One?

Dear Dr. Amin,

Thank you for submitting your manuscript to PLOS ONE. After careful consideration, we feel that it has merit but does not fully meet PLOS ONE’s publication criteria as it currently stands. Therefore, we invite you to submit a revised version of the manuscript that addresses the points raised during the review process.

Please submit your revised manuscript by Sep 03 2026 11:59PM. If you will need more time than this to complete your revisions, please reply to this message or contact the journal office at plosone@plos.org. When you're ready to submit your revision, log on to https://www.editorialmanager.com/pone/ and select the 'Submissions Needing Revision' folder to locate your manuscript file.

- A letter that responds to each point raised by the academic editor and reviewer(s). You should upload this letter as a separate file labeled 'Response to Reviewers'.
- A marked-up copy of your manuscript that highlights changes made to the original version. You should upload this as a separate file labeled 'Revised Manuscript with Track Changes'.
- An unmarked version of your revised paper without tracked changes. You should upload this as a separate file labeled 'Manuscript'.

If you would like to make changes to your financial disclosure, please include your updated statement in your cover letter. Guidelines for resubmitting your figure files are available below the reviewer comments at the end of this letter.

If applicable, we recommend that you deposit your laboratory protocols in protocols.io to enhance the reproducibility of your results. Protocols.io assigns your protocol its own identifier (DOI) so that it can be cited independently in the future. For instructions see: https://journals.plos.org/plosone/s/submission-guidelines#loc-laboratory-protocols. Additionally, PLOS ONE offers an option for publishing peer-reviewed Lab Protocol articles, which describe protocols hosted on protocols.io. Read more information on sharing protocols at https://plos.org/protocols?utm_medium=editorial-email&utm_source=authorletters&utm_campaign=protocols.

As the corresponding author, your ORCID iD is verified in the submission system and will appear in the published article. PLOS supports the use of ORCID, and we encourage all coauthors to register for an ORCID iD and use it as well. Please encourage your coauthors to verify their ORCID iD within the submission system before final acceptance, as unverified ORCID iDs will not appear in the published article. *Only*  the individual author can complete the verification step; PLOS staff *cannot*  verify ORCID iDs on behalf of authors.

We look forward to receiving your revised manuscript.

Kind regards,

Alison Parker

Academic Editor

PLOS One

Journal Requirements:

If the reviewer comments include a recommendation to cite specific previously published works, please review and evaluate these publications to determine whether they are relevant and should be cited. There is no requirement to cite these works unless the editor has indicated otherwise.

[Note: HTML markup is below. Please do not edit.]

Reviewers' comments:

Reviewer's Responses to Questions

**Comments to the Author**

Reviewer #1: All comments have been addressed

Reviewer #3: (No Response)

**********

2. Is the manuscript technically sound, and do the data support the conclusions?

Reviewer #1: Yes

Reviewer #3: Partly

**********

3. Has the statistical analysis been performed appropriately and rigorously?

Reviewer #1: Yes

Reviewer #3: Yes

**********

4. Have the authors made all data underlying the findings in their manuscript fully available?

The PLOS Data policy

Reviewer #1: Yes

Reviewer #3: Yes

**********

5. Is the manuscript presented in an intelligible fashion and written in standard English?

Reviewer #1: Yes

Reviewer #3: Yes

**********

Reviewer #1: The authors have adequately addressed all comments and suggestions raised during the previous round of review. The revisions have improved the clarity and quality of the manuscript. The study is technically sound, the statistical analyses are appropriate, and the conclusions are supported by the data presented. The manuscript is well written, and the data underlying the findings are available in accordance with the journal's requirements.

I have no further comments and recommend the manuscript for publication.

Reviewer #3: Thank you for this study analysis and for this revised manuscript. As I noted in my original review, the authors address a critical question about the relationship between awareness and affordability in driving the use of menstrual products. In the attachment, I’m including a more detailed review with additional comments, but as I did last time, wanted to pull up a few overarching suggestions to strengthen the manuscript’s analysis, discussion, and interpretation/application to menstrual health programs:

(1) In my initial review, I suggested that there was a need for a much stronger discussion on why education is a proxy for awareness – as well as a stronger explanation of what awareness refers to. While this has been significantly strengthened in the second iteration of the paper, I am still not left feeling sold on this approach. Both proxy indicators – educational attainment and household income – lack nuance in how they are cross-walked to awareness and affordability, respectively. My suggestion would be to either: re-focus the paper on educational attainment and household income, and the impacts of these variables on sanitary napkin use OR further develop the proxy analysis.

I suggest doing the first option. There is not a compelling reason to me that the paper needs to focus on awareness rather than educational attainment, or on household income rather than affordability. I think that re-focusing the paper would make a much clearer, much more compelling analysis on the factors that influence sanitary napkin use. You could then, in the discussion/conclusion sections, layer in how these variables contribute to overall awareness or affordability. You would be in a position of having fewer limitations/restrictions to the analysis that you offer.

If you go with the second option, I would suggest: (1) acknowledging that several variables you discuss contribute to awareness, for example media exposure in addition to educational attainment. It would be helpful to pull each of these together and use them as different proxies for awareness, but rolled up into a more cohesive analysis; (2) acknowledging that educational attainment is flawed as a proxy variable. Much of your paper focuses on how educational curriculum doesn’t include menstrual health or how young adolescents are unaware of menstrual hygiene at the age of menarche. This is coupled with the reality that the most vulnerable populations are often the ones not in school. To use this proxy, I suggest being upfront that you recognize these contradictions and limitations, and posing why the analysis still holds. Then, I would suggest strengthening the affordability analysis. In order for household income to be a proxy for affordability, you need some analysis on how product prices are affordable within the context of these different income amounts. How does household income relate to disposable income, for example? And what is the relationship to product prices. Again, I think going with option #1 will feel much more doable and succinct in the context of this analysis – and then you can bring these types of issues into the limitations and discussion.

(2) I would still love to see a stronger discussion of sanitary napkin use. The authors did a great job of better defining ‘sanitary napkins’ in this revision, but still don’t include any discussion on why the analysis is specific to sanitary napkins and not a broader suite of menstrual products (e.g. inclusion of cups, reusable sanitary napkins, etc). The focus on sanitary napkins is fine, but should include a justification for why other products aren’t included. Is this a limitation of the data available? Is it that sanitary napkins are a high enough percent of the products used in Bangladesh that there is not a reason to ask about other products? More discussion here would be valuable.

(3) Finally, I still think that a title modification might be valuable. Since your key takeaway is essentially that both awareness and affordability matter for menstrual health, positing them as potentially conflicting doesn't resonate as much to me. Given my comments under point #2, I also think that focusing more on educational attainment/household income makes the most sense in the title.

**********

what does this mean?). If published, this will include your full peer review and any attached files.

If you choose “no”, your identity will remain anonymous but your review may still be made public.

**Do you want your identity to be public for this peer review?**  For information about this choice, including consent withdrawal, please see our Privacy Policy

Reviewer #1: No

Reviewer #3: No

**********

[NOTE: If reviewer comments were submitted as an attachment file, they will be attached to this email and accessible via the submission site. Please log into your account, locate the manuscript record, and check for the action link "View Attachments". If this link does not appear, there are no attachment files.]

To ensure your figures meet our technical requirements, please review our figure guidelines: https://journals.plos.org/plosone/s/figures

You may also use PLOS’s free figure tool, NAAS, to help you prepare publication quality figures: https://journals.plos.org/plosone/s/figures#loc-tools-for-figure-preparation.

NAAS will assess whether your figures meet our technical requirements by comparing each figure against our figure specifications.

Attachment Submitted filename: PONE-D-26-13387_R1_Peer Reviewer Comments.pdf

# Author comment: Author response to Decision Letter 2

**DOI:** 10.1371/journal.pone.0346457.r004

29 Jul 2026

Reviewer 03:

Comment 1: In my initial review, I suggested that there was a need for a much stronger discussion on why education is a proxy for awareness – as well as a stronger explanation of what awareness refers to. While this has been significantly strengthened in the second iteration of the paper, I am still not left feeling sold on this approach. Both proxy indicators – educational attainment and household income – lack nuance in how they are cross-walked to awareness and affordability, respectively. My suggestion would be to either: re-focus the paper on educational attainment and household income, and the impacts of these variables on sanitary napkin use OR further develop the proxy analysis.

I suggest doing the first option. There is not a compelling reason to me that the paper needs to focus on awareness rather than educational attainment, or on household income rather than affordability. I think that re-focusing the paper would make a much clearer, much more compelling analysis on the factors that influence sanitary napkin use. You could then, in the discussion/conclusion sections, layer in how these variables contribute to overall awareness or affordability. You would be in a position of having fewer limitations/restrictions to the analysis that you offer.

If you go with the second option, I would suggest: (1) acknowledging that several variables you discuss contribute to awareness, for example media exposure in addition to educational attainment. It would be helpful to pull each of these together and use them as different proxies for awareness, but rolled up into a more cohesive analysis; (2) acknowledging that educational attainment is flawed as a proxy variable. Much of your paper focuses on how educational curriculum doesn’t include menstrual health or how young adolescents are unaware of menstrual hygiene at the age of menarche. This is coupled with the reality that the most vulnerable populations are often the ones not in school. To use this proxy, I suggest being upfront that you recognize these contradictions and limitations, and posing why the analysis still holds. Then, I would suggest strengthening the affordability analysis. In order for household income to be a proxy for affordability, you need some analysis on how product prices are affordable within the context of these different income amounts. How does household income relate to disposable income, for example? And what is the relationship to product prices. Again, I think going with option #1 will feel much more doable and succinct in the context of this analysis – and then you can bring these types of issues into the limitations and discussion.

Response 01: Thank you for this thoughtful and constructive suggestion. We agree that educational attainment and household wealth are more appropriately interpreted as socioeconomic determinants rather than direct proxies for awareness and affordability. Accordingly, we have revised the manuscript to adopt your first suggestion by refocusing the analysis on the combined roles of educational attainment and household wealth in sanitary napkin use. References to awareness and affordability as proxy measures have been removed from the main analysis and are now discussed only as potential mechanisms underlying the observed associations.

Comment 02: I would still love to see a stronger discussion of sanitary napkin use. The authors did a great job of better defining ‘sanitary napkins’ in this revision, but still don’t include any discussion on why the analysis is specific to sanitary napkins and not a broader suite of menstrual products (e.g. inclusion of cups, reusable sanitary napkins, etc). The focus on sanitary napkins is fine, but should include a justification for why other products aren’t included. Is this a limitation of the data available? Is it that sanitary napkins are a high enough percent of the products used in Bangladesh that there is not a reason to ask about other products? More discussion here would be valuable.

Response 02: Thank you for this suggestion. We have addressed this point in the Introduction (Lines 83–92) by explaining that, although other modern menstrual products are available, their use remains limited in Bangladesh. As sanitary napkins are the most widely accepted and readily available modern menstrual product, we justify their use as the study outcome.

Comment 03: Finally, I still think that a title modification might be valuable. Since your key takeaway is essentially that both awareness and affordability matter for menstrual health, positing them as potentially conflicting doesn't resonate as much to me. Given my comments under point #2, I also think that focusing more on educational attainment/household income makes the most sense in the title.

Response 03: Thank you for the helpful suggestion. We have revised the title to better reflect the study's main focus to “The Combined Role of Education and Wealth in Menstrual Hygiene: Evidence from Bangladesh”.

Attachment Submitted filename: Rebuttal letter.pdf

# Peer review: Decision Letter 2

**DOI:** 10.1371/journal.pone.0346457.r005

**Authors:** Alison Parker

7 Sep 2026

Dear Dr. Amin,

Thank you for submitting your manuscript to PLOS One. After careful consideration, we feel that it has merit but does not fully meet PLOS One’s publication criteria as it currently stands. Therefore, we invite you to submit a revised version of the manuscript that addresses the points raised during the review process.

I appreciate you've already revised the manuscript twice but reviewer 2 has some more important comments to address.   I hope you feel that these will improve the manuscript and they don't take too much time.

Please submit your revised manuscript by Oct 22 2026 11:59PM. If you will need more time than this to complete your revisions, please reply to this message or contact the journal office at plosone@plos.org. When you're ready to submit your revision, log on to https://www.editorialmanager.com/pone/ and select the 'Submissions Needing Revision' folder to locate your manuscript file.

- A letter that responds to each point raised by the academic editor and reviewer(s). You should upload this letter as a separate file labeled 'Response to Reviewers'.
- A marked-up copy of your manuscript that highlights changes made to the original version. You should upload this as a separate file labeled 'Revised Manuscript with Track Changes'.
- An unmarked version of your revised paper without tracked changes. You should upload this as a separate file labeled 'Manuscript'.

If you would like to make changes to your financial disclosure, please include your updated statement in your cover letter. Guidelines for resubmitting your figure files are available below the reviewer comments at the end of this letter.

If applicable, we recommend that you deposit your laboratory protocols in protocols.io to enhance the reproducibility of your results. Protocols.io assigns your protocol its own identifier (DOI) so that it can be cited independently in the future. For instructions see: https://journals.plos.org/plosone/s/submission-guidelines#loc-laboratory-protocols. Additionally, PLOS One offers an option for publishing peer-reviewed Lab Protocol articles, which describe protocols hosted on protocols.io. Read more information on sharing protocols at https://plos.org/protocols?utm_medium=editorial-email&utm_source=authorletters&utm_campaign=protocols.

As the corresponding author, your ORCID iD is verified in the submission system and will appear in the published article. PLOS supports the use of ORCID, and we encourage all coauthors to register for an ORCID iD and use it as well. Please encourage your coauthors to verify their ORCID iD within the submission system before final acceptance, as unverified ORCID iDs will not appear in the published article. *Only*  the individual author can complete the verification step; PLOS staff *cannot*  verify ORCID iDs on behalf of authors.

We look forward to receiving your revised manuscript.

Kind regards,

Alison Parker

Academic Editor

PLOS One

Journal Requirements:

If the reviewer comments include a recommendation to cite specific previously published works, please review and evaluate these publications to determine whether they are relevant and should be cited. There is no requirement to cite these works unless the editor has indicated otherwise.

Please review your reference list to ensure that it is complete and correct. If you have cited papers that have been retracted, please include the rationale for doing so in the manuscript text, or remove these references and replace them with relevant current references. Any changes to the reference list should be mentioned in the rebuttal letter that accompanies your revised manuscript. If you need to cite a retracted article, indicate the article’s retracted status in the References list and also include a citation and full reference for the retraction notice.

[Note: HTML markup is below. Please do not edit.]

Reviewers' comments:

Reviewer's Responses to Questions

**Comments to the Author**

Reviewer #2: (No Response)

Reviewer #3: All comments have been addressed

**********

2. Is the manuscript technically sound, and do the data support the conclusions?

Reviewer #2: Partly

Reviewer #3: Yes

**********

3. Has the statistical analysis been performed appropriately and rigorously?

Reviewer #2: Yes

Reviewer #3: Yes

**********

4. Have the authors made all data underlying the findings in their manuscript fully available?

The PLOS Data policy

Reviewer #2: Yes

Reviewer #3: Yes

**********

5. Is the manuscript presented in an intelligible fashion and written in standard English?

Reviewer #2: Yes

Reviewer #3: Yes

**********

Reviewer #2: I appreciate the title change, which is more consistent with the article’s content.

I agree with a prior reviewer’s use of “menstrual health” rather than “menstrual hygiene”, with the latter being more stigmatizing and sending an implicit message of judgement or blame on the person menstruating. Please go through the document and change “menstrual hygiene” to “menstrual health.” In the opening paragraph, I might make this more explicit: “To address this gap, this study examines the interplay 18 between education and wealth in determining the use of sanitary napkins in Bangladesh, with sanitary napkin use as a proxy for menstrual health. This choice reflects the predominant usage of sanitary napkins for menstrual management in Bangladesh.”

Making it more clear that use of sanitary napkins may represent a measure of wealth rather than a measure of menstrual health literacy would be useful as an opening hypothesis worthy of analysis.

Figure 1 would be better if it also had markings (eg diagonal lines in a printed fashion) in areas of wealth, so that we can see at a glance if the high usage areas corresponded with top tier wealth.

P9 line 42- would not capitalize menarche: “(aka Menarche)”

P9 line 45- “between the ages of 12 to 15 years”

P9 line 56: Spell out RTI and then put initials in parentheses

P11 line 114. It would be useful to include a power analysis to ascertain that 20 households per group would represent potential significance.

P17 line 193-195: need p values here.

P 20 line 220: correct “This behavibiour”- spellcheck please

p 21 lines 246-250 in the discussion paragraph: Did this vary by income level? Did patriarchal high income households still have less sanitary napkin use than matriarchal high income households? How did educational level impact this finding?

P 21 line 251- did you mean “seem to have a significant role” rather than “seen to have a significant role”?

P 21 paragraph 251-257- the jump from mass media exposure to menstruation and disability is abrupt with no transition. Are women who are exposed to more mass media more or less likely to be of higher educational background? How does that relate to income? The sentences on disability should be in their own paragraph and have more discussion around menstrual health findings for those with disabilities.

P22- line 273-273. Should be are, not is: “This is particularly relevant in low-income households where affordability and availability are the key barriers”

Line 279 and following paragraph. “To reduce costs and promote local manufacturing, the government has waived VAT on raw materials for sanitary napkin production. This move has decreased production costs but has not translated into lowered costs at the consumer level; a 15% VAT on finished sanitary napkin products still impedes uptake, especially in lower income households. The National MHM Coordination Committee has been established to oversee and align MHM efforts across health, education, and social sectors. Advocacy efforts can include eliminating the “pink tax” or VAT at the consumer level, improving distribution networks to ensure access in workplaces, schools, healthcare centers, and public spaces, alongside expanding MHM awareness sessions to marginalized and hard-to-reach communities. In other words, the first priority is reducing the cost across all populations, and second, enhancing acceptability and accessibility.”

P330- Conlusion: should be far more clear on the need to eliminate the “pink tax” on consumer products for menstrual health, specifically sanitary napkins in India but also other menstrual products. Education alone will not suffice; affordability appears to be a significant barrier. Reducing VAT on raw materials clearly does not do enough to lower consumer costs.

It also appears that the authors did not incorporate any of this reviewer’s prior recommendations, including addition of any of these articles to the discussion:

Mittermeier, T ∙ Hawkey, A ∙ Bush, D et al. Menstrual health symptoms and literacy among young women in Aotearoa New Zealand: a nationwide cross-sectional survey. J Pediatr Adolesc Gynecol. 2025; 38:328-335

Bhoda, K ∙ Burgoa, S ∙ Zerrouki, Y ...Examining Menstrual Health and Hygiene Educational Initiatives for American Middle and High School Menstruating Students: A Scoping Review.

J Pediatr Adolesc Gynec. 2024; 37(5):465-476

Harrison, ME ∙ Davies, S ∙ Tyson, N ...NASPAG Position Statement: Eliminating Period Poverty in Adolescents and Young Adults Living in North America. J Pediatr Adolesc Gynecol. 2022; 35(6):609-611

Davies, S ∙ Darien, K ∙ Bazyar, F ...A Multimodal Qualitative Approach to Understanding Menstrual Health Equity among Adolescents and Young Adults. Journal of Pediatric and Adolescent Gynecology. 2023; 36(6):511-517

Armour M, Hyman MK et al. Menstrual Health Literacy and Management Strategies in Young Women in Australia: A National Online Survey of Young Women Aged 13-25 Years. Journal of Pediatric and Adolescent GynecologyVol. 34Issue 2p135–143Published online: November 11, 2020

Rome ES, Tyson N. Menstrual Equity. Obstet Gynecol Clin North Am. 2024 Dec;51(4):731-744. doi: 10.1016/j.ogc.2024.08.008. Epub 2024 Sep 21

This reviewer believes these articles would add strength to both the intro and discussion sections.

Reviewer #3: Thank you for the opportunity to review this revision again! The changes made to this version make the paper much stronger and you present a strong, compelling analysis. Well done!

**********

what does this mean?). If published, this will include your full peer review and any attached files.

If you choose “no”, your identity will remain anonymous but your review may still be made public.

**Do you want your identity to be public for this peer review?**  For information about this choice, including consent withdrawal, please see our Privacy Policy

Reviewer #2: No

Reviewer #3: **Yes:**  Sarah Webb

**********

[NOTE: If reviewer comments were submitted as an attachment file, they will be attached to this email and accessible via the submission site. Please log into your account, locate the manuscript record, and check for the action link "View Attachments". If this link does not appear, there are no attachment files.]

To ensure your figures meet our technical requirements, please review our figure guidelines: https://journals.plos.org/plosone/s/figures

You may also use PLOS’s free figure tool, NAAS, to help you prepare publication quality figures: https://journals.plos.org/plosone/s/figures#loc-tools-for-figure-preparation.

NAAS will assess whether your figures meet our technical requirements by comparing each figure against our figure specifications.

# Author comment: Author response to Decision Letter 3

**DOI:** 10.1371/journal.pone.0346457.r006

9 Sep 2026

Rebuttal letter

Reviewer #2: I appreciate the title change, which is more consistent with the article’s content.

I agree with a prior reviewer’s use of “menstrual health” rather than “menstrual hygiene”, with the latter being more stigmatizing and sending an implicit message of judgement or blame on the person menstruating.

Comment 1: Please go through the document and change “menstrual hygiene” to “menstrual health.”

Response 1: Thank you for this suggestion. We respectfully retained the term “menstrual hygiene” because our outcome variable specifically measures sanitary napkin use, which reflects a menstrual hygiene practice rather than menstrual health itself. We therefore consider “menstrual hygiene” to be the more appropriate term for describing the outcome and its interpretation in this study.

Comment 2: In the opening paragraph, I might make this more explicit: “To address this gap, this study examines the interplay between education and wealth in determining the use of sanitary napkins in Bangladesh, with sanitary napkin use as a proxy for menstrual health. This choice reflects the predominant usage of sanitary napkins for menstrual management in Bangladesh.”

Making it more clear that use of sanitary napkins may represent a measure of wealth rather than a measure of menstrual health literacy would be useful as an opening hypothesis worthy of analysis.

Response 2: We have added the suggested text to the opening paragraph (lines 18-20).

Comment 3: Figure 1 would be better if it also had markings (eg diagonal lines in a printed fashion) in areas of wealth, so that we can see at a glance if the high usage areas corresponded with top tier wealth.

Response 3: We respectfully chose not to add wealth overlays to Figure 1, as including additional layers could create visual clutter and reduce the clarity of the map. The relationship between wealth quintiles and sanitary napkin use is presented in greater detail in Figures 2 and 3.

Comment 4:

P9 line 42- would not capitalize menarche: “(aka Menarche)”

P9 line 45- “between the ages of 12 to 15 years”

P9 line 56: Spell out RTI and then put initials in parentheses

Response 4: All three minor corrections have been made as suggested.

Comment 5: P11 line 114. It would be useful to include a power analysis to ascertain that 20 households per group would represent potential significance.

Response 5: Thank you for the feedback. As this study used secondary data from the nationally representative 2019 Bangladesh MICS, the sample size was determined by the survey design. Also, the detail power analysis is presented in the 2019 Bangladesh MICS report.

Comment 6: P17 line 193-195: need p values here.

Response 6: We included p-values from a binary logistic regression model evaluating the combined wealth–education subgroups, treating the "Secondary or above & Bottom two wealth" group as the reference category.

Comment 7: P 20 line 220: correct “This behavibiour”- spellcheck please

Response 7: Thank you for pointing this out. We have corrected the spelling.

Comment 8: p 21 lines 246-250 in the discussion paragraph: Did this vary by income level? Did patriarchal high income households still have less sanitary napkin use than matriarchal high income households? How did educational level impact this finding?

Response 8: While an important query, evaluating multi-way interaction effects between household headship, wealth, and education was beyond the defined scope of this study. We have noted this as a key area for future research.

Comment 9: P 21 line 251- did you mean “seem to have a significant role” rather than “seen to have a significant role”?

Response 9: Thank you for pointing this out. We have corrected the spelling.

Comment 10: P 21 paragraph 251-257- the jump from mass media exposure to menstruation and disability is abrupt with no transition. Are women who are exposed to more mass media more or less likely to be of higher educational background? How does that relate to income? The sentences on disability should be in their own paragraph and have more discussion around menstrual health findings for those with disabilities.

Response 10: We have fixed the transition. Regarding the effect of mass media, our analysis was not focused on assessing its interaction effect with education, as a result current analysis can not answer that particular question.

Additionally, we have deepened the argument regarding the disability as suggest by the reviewer.

Comment 11: P22- line 273-273. Should be are, not is: “This is particularly relevant in low-income households where affordability and availability are the key barriers”

Response 11: Thank you for pointing this out. We have corrected it.

Comment 12: Line 279 and following paragraph. “To reduce costs and promote local manufacturing, the government has waived VAT on raw materials for sanitary napkin production. This move has decreased production costs but has not translated into lowered costs at the consumer level; a 15% VAT on finished sanitary napkin products still impedes uptake, especially in lower income households. The National MHM Coordination Committee has been established to oversee and align MHM efforts across health, education, and social sectors. Advocacy efforts can include eliminating the “pink tax” or VAT at the consumer level, improving distribution networks to ensure access in workplaces, schools, healthcare centers, and public spaces, alongside expanding MHM awareness sessions to marginalized and hard-to-reach communities. In other words, the first priority is reducing the cost across all populations, and second, enhancing acceptability and accessibility.”

Response 12: We have replaced the paragraph with the provided text.

Comment 13: P330- Conlusion: should be far more clear on the need to eliminate the “pink tax” on consumer products for menstrual health, specifically sanitary napkins in India but also other menstrual products. Education alone will not suffice; affordability appears to be a significant barrier. Reducing VAT on raw materials clearly does not do enough to lower consumer costs.

Response 13: We updated the conclusion to highlight that education alone is insufficient and that eliminating consumer-level taxes on finished menstrual products is essential for overcoming affordability barriers in Bangladesh.

Comment 14: It also appears that the authors did not incorporate any of this reviewer’s prior recommendations, including addition of any of these articles to the discussion:

Mittermeier, T ∙ Hawkey, A ∙ Bush, D et al. Menstrual health symptoms and literacy among young women in Aotearoa New Zealand: a nationwide cross-sectional survey. J Pediatr Adolesc Gynecol. 2025; 38:328-335

Bhoda, K ∙ Burgoa, S ∙ Zerrouki, Y ...Examining Menstrual Health and Hygiene Educational Initiatives for American Middle and High School Menstruating Students: A Scoping Review.

J Pediatr Adolesc Gynec. 2024; 37(5):465-476

Harrison, ME ∙ Davies, S ∙ Tyson, N ...NASPAG Position Statement: Eliminating Period Poverty in Adolescents and Young Adults Living in North America. J Pediatr Adolesc Gynecol. 2022; 35(6):609-611

Davies, S ∙ Darien, K ∙ Bazyar, F ...A Multimodal Qualitative Approach to Understanding Menstrual Health Equity among Adolescents and Young Adults. Journal of Pediatric and Adolescent Gynecology. 2023; 36(6):511-517

Armour M, Hyman MK et al. Menstrual Health Literacy and Management Strategies in Young Women in Australia: A National Online Survey of Young Women Aged 13-25 Years. Journal of Pediatric and Adolescent GynecologyVol. 34Issue 2p135–143Published online: November 11, 2020

Rome ES, Tyson N. Menstrual Equity. Obstet Gynecol Clin North Am. 2024 Dec;51(4):731-744. doi: 10.1016/j.ogc.2024.08.008. Epub 2024 Sep 21

Response 14: We thank the reviewer for suggesting these important materials. We have carefully read and added these as reference where appropriate.

Attachment Submitted filename: Rebuttal_Letter_auresp_3.pdf

# Editor report: Decision Letter 3

**DOI:** 10.1371/journal.pone.0346457.r007

**Authors:** Alison Parker

13 Sep 2026

Dear Dr. Amin,

plosone@plos.org. When you're ready to submit your revision, log on to https://www.editorialmanager.com/pone/ and select the 'Submissions Needing Revision' folder to locate your manuscript file.

- A letter that responds to each point raised by the academic editor and reviewer(s). You should upload this letter as a separate file labeled 'Response to Reviewers'.
- A marked-up copy of your manuscript that highlights changes made to the original version. You should upload this as a separate file labeled 'Revised Manuscript with Track Changes'.
- An unmarked version of your revised paper without tracked changes. You should upload this as a separate file labeled 'Manuscript'.

If you would like to make changes to your financial disclosure, please include your updated statement in your cover letter. Guidelines for resubmitting your figure files are available below the reviewer comments at the end of this letter.

If applicable, we recommend that you deposit your laboratory protocols in protocols.io to enhance the reproducibility of your results. Protocols.io assigns your protocol its own identifier (DOI) so that it can be cited independently in the future. For instructions see: https://journals.plos.org/plosone/s/submission-guidelines#loc-laboratory-protocols. Additionally, PLOS One offers an option for publishing peer-reviewed Lab Protocol articles, which describe protocols hosted on protocols.io. Read more information on sharing protocols at https://plos.org/protocols?utm_medium=editorial-email&utm_source=authorletters&utm_campaign=protocols.

As the corresponding author, your ORCID iD is verified in the submission system and will appear in the published article. PLOS supports the use of ORCID, and we encourage all coauthors to register for an ORCID iD and use it as well. Please encourage your coauthors to verify their ORCID iD within the submission system before final acceptance, as unverified ORCID iDs will not appear in the published article. *Only*  the individual author can complete the verification step; PLOS staff *cannot*  verify ORCID iDs on behalf of authors.

We look forward to receiving your revised manuscript.

Kind regards,

Alison Parker

Academic Editor

PLOS One

Journal Requirements:

If the reviewer comments include a recommendation to cite specific previously published works, please review and evaluate these publications to determine whether they are relevant and should be cited. There is no requirement to cite these works unless the editor has indicated otherwise.

[Note: HTML markup is below. Please do not edit.]

[NOTE: If reviewer comments were submitted as an attachment file, they will be attached to this email and accessible via the submission site. Please log into your account, locate the manuscript record, and check for the action link "View Attachments". If this link does not appear, there are no attachment files.]

To ensure your figures meet our technical requirements, please review our figure guidelines: https://journals.plos.org/plosone/s/figures

You may also use PLOS’s free figure tool, NAAS, to help you prepare publication quality figures: https://journals.plos.org/plosone/s/figures#loc-tools-for-figure-preparation.

NAAS will assess whether your figures meet our technical requirements by comparing each figure against our figure specifications.

# Author comment: Author response to Decision Letter 4

**DOI:** 10.1371/journal.pone.0346457.r008

15 Sep 2026

Rebuttal letter

Comment 1: Please go through the document and change “menstrual hygiene” to “menstrual health.”

Response 1: We thank the reviewer for this suggestion. The term "menstrual health" has been updated throughout the manuscript accordingly.

Comment 3: Figure 1 would be better if it also had markings (eg diagonal lines in a printed fashion) in areas of wealth, so that we can see at a glance if the high usage areas corresponded with top tier wealth.

Response 3: We thank the reviewer for this helpful suggestion. Figure 1 has been updated to include diagonal hatching across high-wealth districts (>60% upper wealth quintiles) to allow for immediate visual comparison with usage levels. The figure caption and Methods section have been updated accordingly.

Comment 5: P11 line 114. It would be useful to include a power analysis to ascertain that 20 households per group would represent potential significance.

Response 5: We thank the reviewer for this comment. We have now added the power analysis in lines 118-122 of the Methods section.

Attachment Submitted filename: Rebuttal_Letter_auresp_4.pdf

# Editor report: Decision Letter 4

**DOI:** 10.1371/journal.pone.0346457.r009

**Authors:** Alison Parker

17 Sep 2026

The Combined Role of Education and Wealth in Menstrual Health: Evidence from Bangladesh

PONE-D-26-13387R4

Dear Dr. Amin,

We’re pleased to inform you that your manuscript has been judged scientifically suitable for publication and will be formally accepted for publication once it meets all outstanding technical requirements.

Within one week, you’ll receive an e-mail detailing the required amendments. When these have been addressed, you’ll receive a formal acceptance letter and your manuscript will be scheduled for publication.

An invoice will be generated when your article is formally accepted. Please note, if your institution has a publishing partnership with PLOS and your article meets the relevant criteria, all or part of your publication costs will be covered. Please make sure your user information is up-to-date by logging into Editorial Manager at Editorial Manager® and clicking the ‘Update My Information' link at the top of the page. For questions related to billing, please contact billing support.

If your institution or institutions have a press office, please notify them about your upcoming paper to help maximize its impact. If they’ll be preparing press materials, please inform our press team as soon as possible -- no later than 48 hours after receiving the formal acceptance. Your manuscript will remain under strict press embargo until 2 pm Eastern Time on the date of publication. For more information, please contact onepress@plos.org.

Kind regards,

Alison Parker

Academic Editor

PLOS One

Additional Editor Comments (optional):

Reviewers' comments:

# Editor report: Acceptance letter

**DOI:** 10.1371/journal.pone.0346457.r010

**Authors:** Alison Parker

PONE-D-26-13387R4

PLOS One

Dear Dr. Amin,

I'm pleased to inform you that your manuscript has been deemed suitable for publication in PLOS One. Congratulations! Your manuscript is now being handed over to our production team.

At this stage, our production department will prepare your paper for publication. This includes ensuring the following:

* All references, tables, and figures are properly cited

* All relevant supporting information is included in the manuscript submission,

* There are no issues that prevent the paper from being properly typeset

You will receive further instructions from the production team, including instructions on how to review your proof when it is ready. Please keep in mind that we are working through a large volume of accepted articles, so please give us a few days to review your paper and let you know the next and final steps.

Lastly, if your institution or institutions have a press office, please let them know about your upcoming paper now to help maximize its impact. If they'll be preparing press materials, please inform our press team within the next 48 hours. Your manuscript will remain under strict press embargo until 2 pm Eastern Time on the date of publication. For more information, please contact onepress@plos.org.

You will receive an invoice from PLOS for your publication fee after your manuscript has reached the completed accept phase. If you receive an email requesting payment before acceptance or for any other service, this may be a phishing scheme. Learn how to identify phishing emails and protect your accounts at https://explore.plos.org/phishing.

If we can help with anything else, please email us at customercare@plos.org.

Thank you for submitting your work to PLOS One and supporting open access.

Kind regards,

PLOS One Editorial Office Staff

on behalf of

Dr. Alison Parker

Academic Editor

PLOS One
